# The high potential of methyl laurate as a recyclable competitor to conventional toxic solvents in [3 + 2] cycloaddition reactions

**DOI:** 10.3762/bjoc.21.184

**Published:** 2025-11-05

**Authors:** Ayhan Yıldırım, Mustafa Göker

**Affiliations:** 1 Department of Chemistry, Bursa Uludağ University, Bursa 16059, Turkey

**Keywords:** cycloaddition, fused isoxazolidines, renewable solvent, reusable solvent, *trans*-diastereoselectivity

## Abstract

In the present study, 21 fused isoxazolidines were synthesized in yields ranging from good to excellent. Methyl laurate was identified as the easily accessible optimal solvent medium for the reaction, and the related compounds were obtained through straightforward isolation techniques in a relatively short time frame (5–80 minutes). A comprehensive investigation was conducted utilizing various web platforms, encompassing ecological and environmental risk assessments, toxicity, pesticide similarity, and biodegradability of methyl laurate in comparison with a series of conventional organic solvents, water, some fatty acids and their derivatives. The findings of this investigation revealed that methyl laurate exhibited better green solvent properties when evaluated against other solvents.

## Introduction

It is an established fact that a significant number of conventional organic solvents, which are widely utilized in both industrial and academic contexts, have deleterious effects on human and environmental health [1–3]. There is an ongoing and intensive research effort to identify new biocompatible alternatives to replace these existing solvents and many of the criteria that a solvent must meet to be considered green have been well defined in different sources [4–5]. In order to fulfil this requirement, a considerable number of green solvents of various classes have been developed for a range of applications, including the extraction of natural compounds [6–9], food analysis [10–12], pharmacology [13–15], and organic synthesis [16–19]. Despite the advent of environmentally friendly green solvents that have been discovered to be applicable in numerous modern organic chemical transformations, difficulties often arise in the recovery and reuse of these solvents following the completion of the reaction. Moreover, some of these solvents have been observed to degrade under conditions that are particularly severe [20]. For instance, although ionic liquids are well-known green solvent alternatives with superior properties compared to conventional organic solvents, their recovery from the reaction medium can be quite troublesome [21–22]. It is evident that this class of solvents may be accompanied by a number of drawbacks. For instance, they are frequently expensive, exhibit negligible or non-existent biodegradability, and there is a lack of data concerning their potential toxicity. Conversely, while water is regarded as a promising biocompatible solvent alternative in numerous chemical transformations, there are several challenges associated with product isolation that must be addressed [23]. Consequently, it is evident that the endeavor to identify an optimal green solvent for both industrial and academic applications must persist.

**Figure 1:**
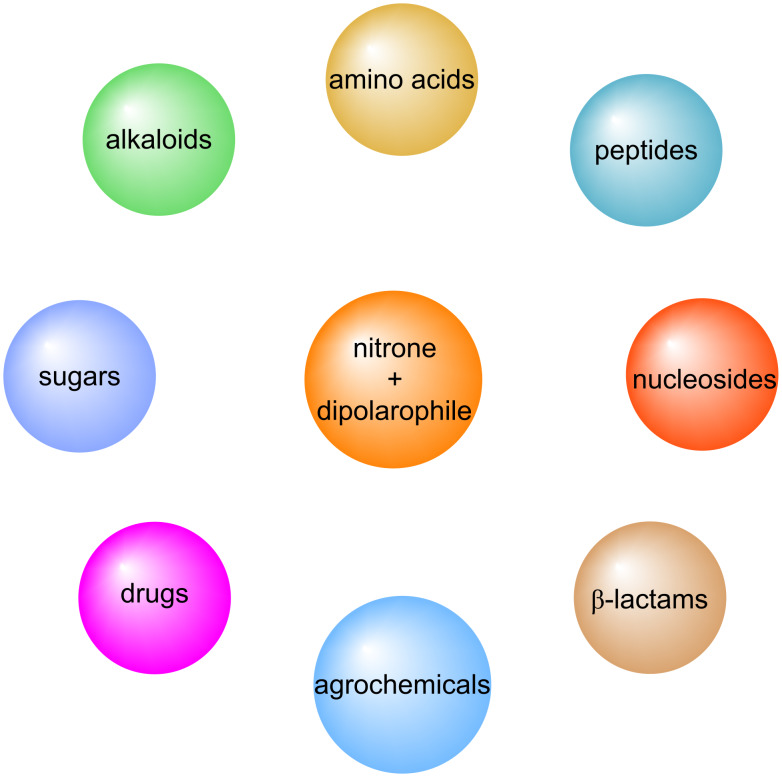
Versatile compounds via cycloaddition reactions.

In the modern world, one of the main goals of an increasing number of chemists interested in the design and synthesis of versatile organic molecules is to develop atom-efficient, multicomponent, low-cost, and environmentally benign synthetic strategies for these molecules. In the context of these strategies, it would be judicious to consider cycloaddition reactions of the [3 + 2] type, a field in which Smith and Huisgen are recognized as pioneers [24–25]. In the field of heterocyclic chemistry, the [3 + 2] type of cycloaddition reaction is a widely employed method for the formation of the five-membered isoxazolidine ring motif, which displays a range of biologically active properties [26–30]. It is notable that the fused pyrrolo-isoxazolidines represent a particularly versatile class of heterocyclic compounds and intermediates, which have been demonstrated to exhibit a wide range of biological activities [26,28,31–35]. The validity of the atom-efficient methodology under discussion has been demonstrated in experimental research; its efficacy in facilitating the rapid formation of multifunctional complex molecules being in contradistinction to the time- and labor-intensive nature of traditional multistep synthesis strategies. For instance, this methodology has been demonstrated to be highly advantageous in the synthesis of numerous pharmaceutical compounds, biological probes, insecticides, alkaloids, and other intricate natural compounds consisting of a combination of isoxazolidine rings [36–41]. It has been demonstrated that such cycloaddition reactions are also employed in the efficient preparation of biologically active molecules, including nucleosides, β-lactam class antibiotics, peptides, and amino acids, as well as sugars (Figure 1) [42–48].

More specifically cycloaddition reactions of nitrones (1,3-dipoles) with *N*-aryl-substituted maleimides (electron-poor dipolarophiles) are a highly popular and versatile method for the formation of regio- and stereoselective pyrrolo-isoxazolidine-fused ring scaffolds. From the standpoint of organic chemistry, it is evident that these compounds can be efficiently converted into a variety of versatile organic intermediates through the application of ring-opening reactions [49–52]. In addition to the aforementioned useful properties, the current methods for the preparation of such cyclic compounds and/or their fused cyclic systems typically necessitate the use of toxic solvents, including chloroform, benzene, toluene etc. [32,53–59]. Indeed, the selection of conventional organic solvents, including benzene, toluene and chloroform, for the [3 + 2] cycloaddition reactions between nitrones and diverse dipolarophiles has not been predicated on any criteria other than their inertness [60]. Conversely, certain studies have reported that the effects of organic solvents in such reactions are negligible, and that an increase in solvent polarity results in a decrease in rate constants. This phenomenon is attributed to the lower polarity of the transition state in comparison to that of the initial compounds [61]. Furthermore, these methods often require harsh reaction conditions, prolonged reaction times, and laborious purification techniques [62–64]. Synthesis of pyrrolo-isoxazolidines utilizing nitrosoarenes via the multicomponent strategy is indeed feasible [65–66]. In a similar manner, Chakraborty obtained diastereomer products, primarily in cis configuration, from the cycloaddition reaction of a fluoro or a furyl-based nitrone with some maleimides via a mechanochemical route in a solvent-free medium [67–68]. The development of environmentally friendly green methodologies for the preparation of heterocyclic compounds via such cycloaddition reactions is a significant and ongoing research area that should not be overlooked [69]. However, these methods are accompanied by the inevitability of long reaction times or the use of toxic organic solvents. Such negative aspects encountered during the synthesis of these compounds prompted us to seek alternative, environmentally benign, renewable, and reusable solvents [70]. It is therefore a basic tenet of *Green Chemistry* to replace the conventional organic solvents, which are flammable and generate toxic volatile components and are costly, with readily accessible and biocompatible alternatives [71]. Indeed, the utilization of eco-friendly bio-based solvents in a multitude of organic chemical transformations [72–74], industrial processes [5,75–76], and pharmaceutical applications [13,77] has recently gained significant prevalence. In view of the aforementioned considerations, the focus of our current research is on more environmentally friendly 1,3-dipoar cycloaddition reactions of nitrones with *N*-arylmaleimides to synthesize a series of pyrrolo-isoxazolidines.

In this context, an investigation was conducted into the potential of vegetable oils and certain derivatives to serve as biocompatible solvents in cycloaddition reaction contexts. This investigation involved a comparative analysis of these vegetable oils and derivatives with water and a range of commonly employed organic solvents in organic transformations.

## Results and Discussion

**Scheme 1:**
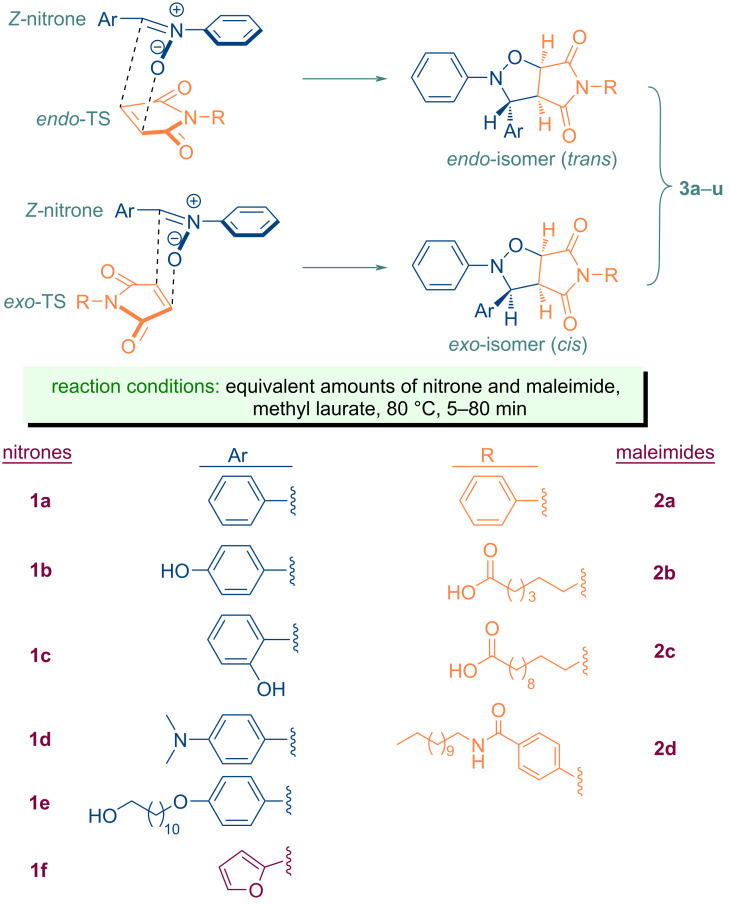
Molecular structures of parent compounds **1a**–**f**, **2a**–**d** and cycloadducts **3a**–**u**.

The requisite starting compounds, nitrones **1a**–**f** and a selection of *N*-substituted maleimide derivatives **2b**–**d** were successfully synthesized in accordance with procedures previously documented in the literature (Scheme 1) [28,78–85]. In this study, the reaction between simple starting compounds *C*,*N*-diphenylnitrone and *N*-phenylmaleimide was selected as the model cycloaddition reaction. A series of bio-based materials were subsequently employed to ascertain the most appropriate green solvent for the specified cycloaddition reaction, with the findings presented in Table 1. As evidenced by the data presented in the table, the majority of the selected vegetable oils and their two derivatives are viable candidates for use as solvents in the proposed reaction. Concurrently, the reaction was carried out in a solvent-free environment, and the product yields obtained in two distinct time periods are presented in Table 1 (entries 11 and 12). As can be deduced from these results, the reaction is found to be more sluggish in a solvent-free environment. Therefore, as evidenced in Table 1 (entries 8 and 9), methyl laurate is the most suitable solvent candidate for the reaction between nitrones and maleimides. Accordingly, methyl laurate, which facilitates the completion of this reaction in a relatively short time frame (approximately five minutes), was selected as the primary solvent for the isoxazolidine derivatives that are planned to be synthesized in this study.

**Table 1:** Optimization of the [3 + 2] cycloaddition conditions for the synthesis of pyrrolo[3,4-*d*]isoxazolidine **3a** (*cis* + *trans* isomers).

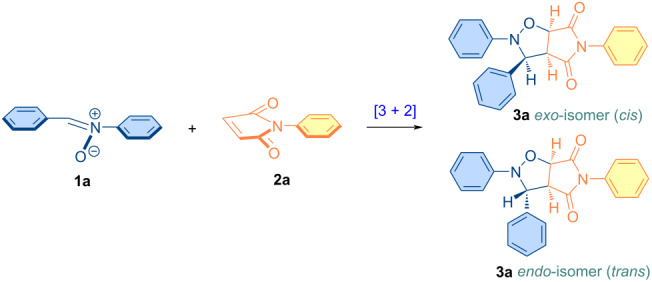				

Entry	Solvent	Time (min)	Temperature (°C)	Yield (%)^a^

1	sunflower oil	10	80	89
2	coconut oil	10	80	91
3	olive oil	10	80	75
4	hazelnut oil	10	80	92
5	walnut oil	10	80	87
6	castor oil	10	80	53
7	oleic acid	10	80	87
8	methyl laurate	10	80	100
9	methyl laurate	5	80	100 (*cis*/*trans* 28:72)
10	water^b^	10	80	51
11	solvent free	10	80	42
12	solvent free	60	80	53

^a^The yields were obtained by precipitation of the relevant product from the reaction medium with a suitable solvent. ^b^The yield was obtained by means of extraction with EtOAc and subsequent precipitation with hexane.

**Figure 2:**
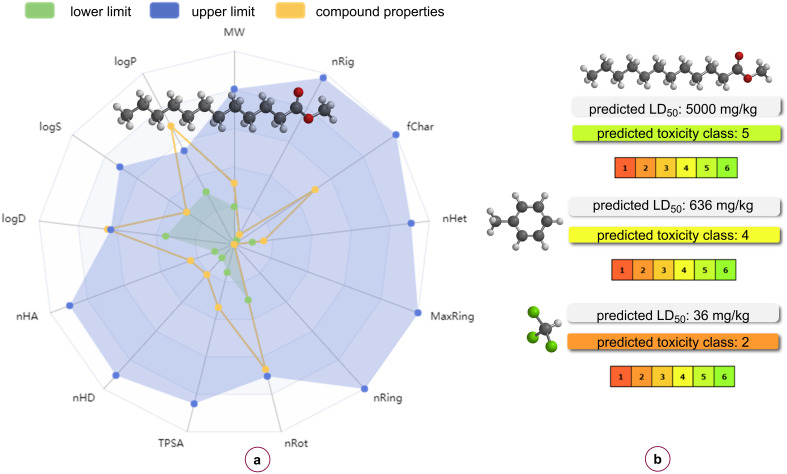
a) Radar view of the physical properties of methyl laurate. b) Oral toxicity values of methyl laurate, toluene, and chloroform, respectively.

**Figure 3:**
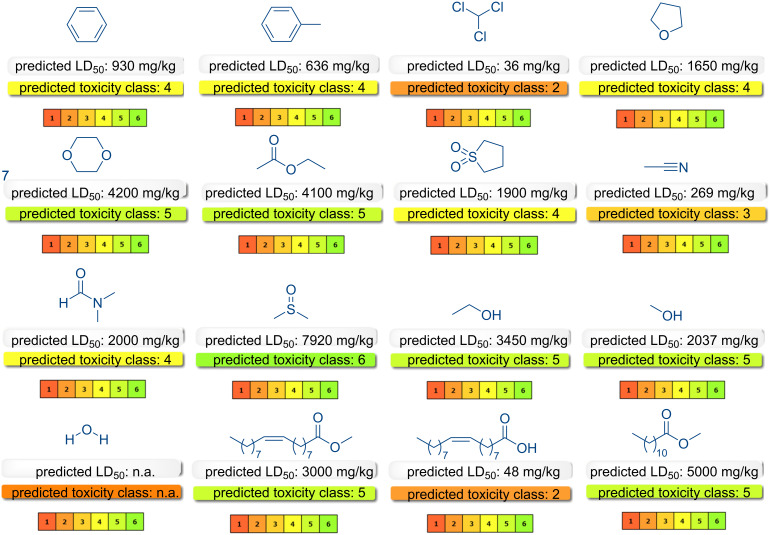
The oral toxicity values of all the solvents utilized in the present study obtained with *ProTox 3.0*.

In recent times, research into the evaluation of ecological and environmental risks posed by various organic compounds and solvents has gained increased significance. In silico models have emerged as a valuable tool, offering rapid and cost-effective solutions when experimental data is not readily available, particularly those that are web-integrated [86]. The predicted physical properties of methyl laurate, as determined by the *ADMETLab 3.0* platform [87] are illustrated in Figure 2a. Conversely, the oral toxicity values of methyl laurate, in conjunction with toluene and chloroform – two conventional solvents that are commonly employed in [3 + 2] cycloaddition reactions were calculated with *ProTox 3.0* (a webserver for the prediction of toxicity of chemicals) [88], as shown in Figure 2b. As illustrated in the figure, the LD_50_ value, which serves as a measure of the toxicity of these solvents, exhibits the highest value for methyl laurate and can be characterized as the solvent with the lowest toxicity. Furthermore, the results for the organic solvents many of which are not environmentally friendly and widely used in other synthesis reactions have been presented in Figure 3. The solvent potential of these solvents in the [3 + 2] cycloaddition reaction was investigated in the context of this study. As demonstrated in Figure 3, the toxicity class estimated for methyl laurate categorizes it as a potential environmentally friendly solvent. The *OSIRIS PropertyExplorer* program was employed to conduct toxicity risk assessments of methyl laurate, the results of which demonstrated that this solvent does not possess mutagenic, tumorigenic, irritant, or reproductive properties (Table 2). According to the toxicity risk assessments for solvents in this table, determined using the *OSIRIS PropertyExplorer* software, sulfolane appears to be a safe solvent and its use is recommended for certain purposes in some studies [89]. However, the toxicity of sulfolane has been mentioned in some other studies [90–91]. Moreover, as illustrated in the Table 2, only methyl oleate and methyl laurate, in conjunction with sulfolane, were categorized as entirely risk-free based on the four pertinent toxicity parameters. In the context of cycloadditions, the conventional solvents that are typically employed as reaction media encompass a range of options, including tetrahydrofuran (THF) (a problematic solvent, *p*), dioxane (considered hazardous, *h*), benzene (designated as highly hazardous, *hh*), toluene (*p*), chloroform (*hh*), acetonitrile (*p*), sulfolane (*h*), and DMSO (*p*) according to the guidelines outlined in the CHEM21 solvent selection guide [92].

**Table 2:** Toxicity risk assessments of the solvents (with *OSIRIS PropertyExplorer*).

Solvent	Mutagenic	Tumorigenic	Irritant	Reproductive effective

benzene	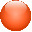	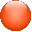	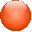	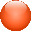
toluene	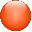	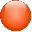	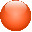	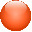
chloroform	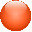	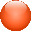	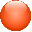	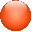
THF	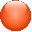	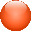	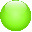	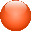
dioxane	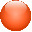	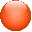	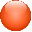	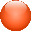
EtOAc	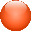	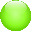	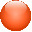	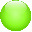
sulfolane	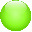	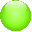	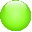	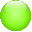
MeCN	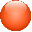	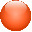	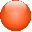	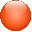
DMF	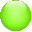	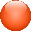	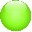	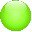
DMSO	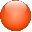	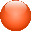	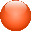	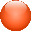
EtOH	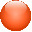	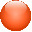	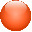	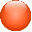
MeOH	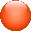	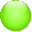	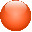	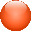
water	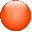	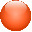	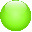	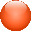
methyl oleate	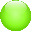	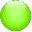	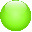	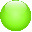
oleic acid	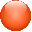	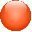	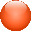	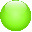
methyl laurate	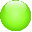	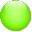	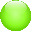	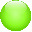

**Figure 4:**
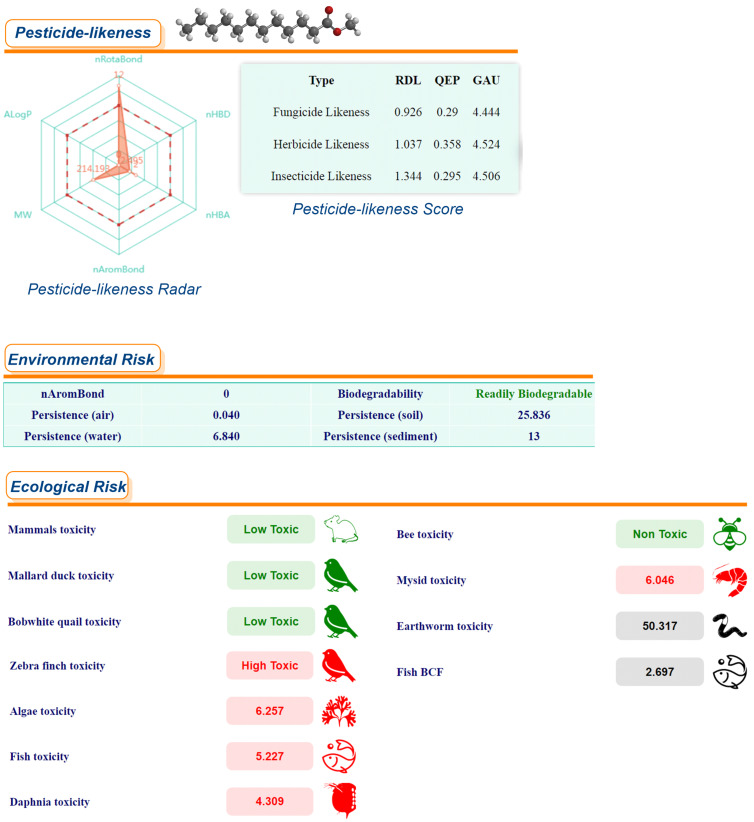
Ecological, environmental risk assessments, pesticide similarity and biodegradability assessments of methyl laurate.

**Figure 5:**
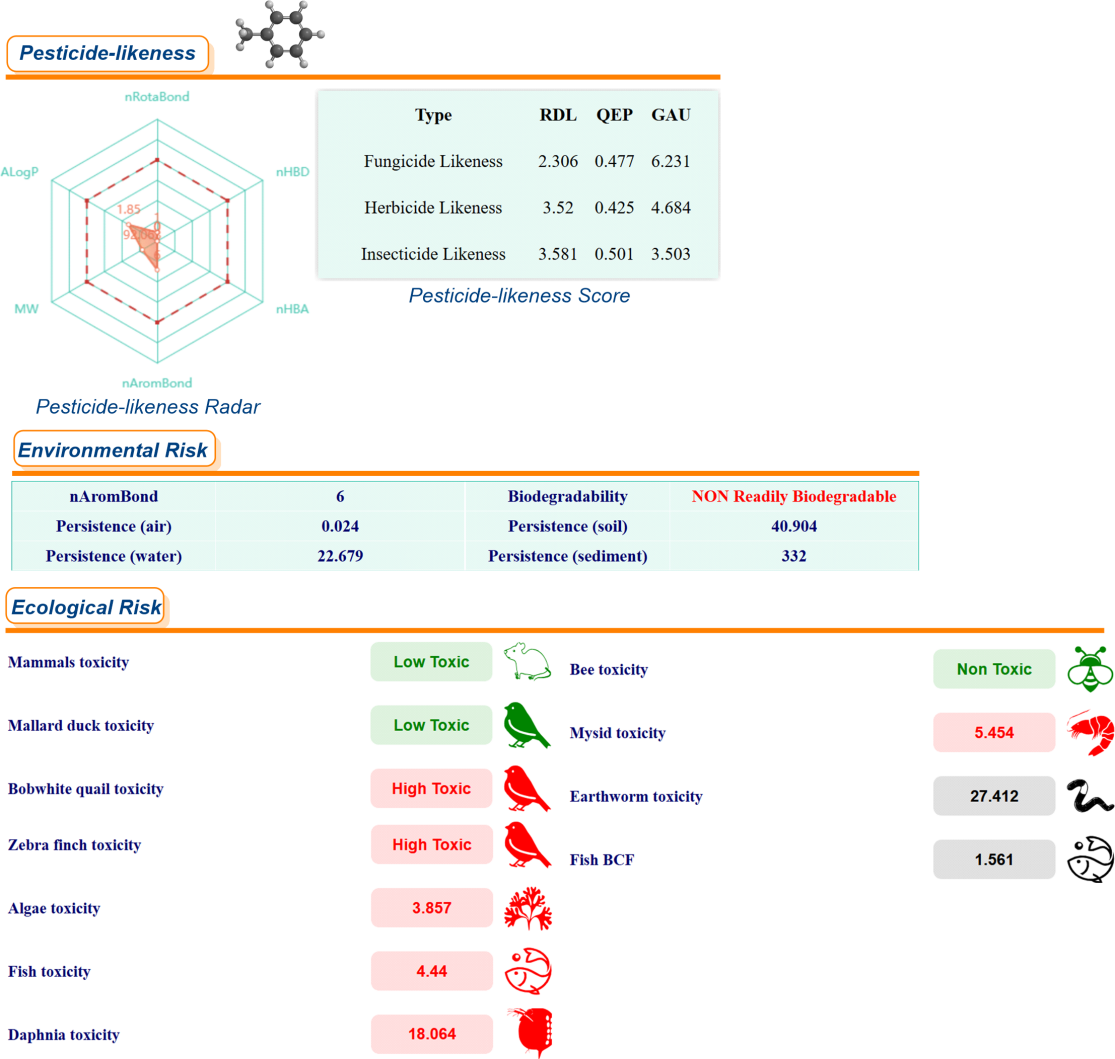
Ecological, environmental risk assessments, pesticide similarity and biodegradability assessments of toluene.

**Figure 6:**
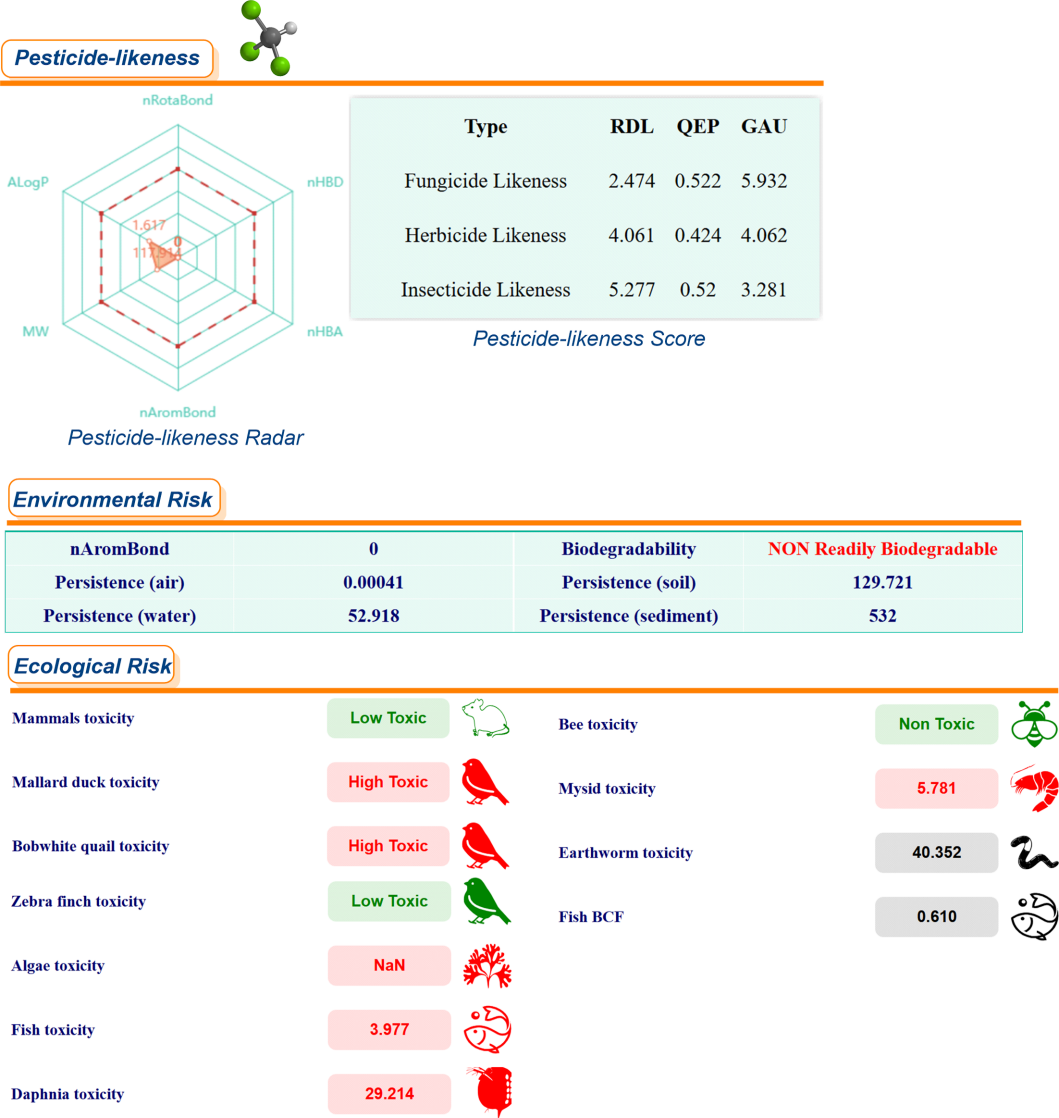
Ecological, environmental risk assessments, pesticide similarity and biodegradability assessments of chloroform.

A series of risk assessments was conducted, including ecological and environmental risk assessments, as well as pesticide similarity and biodegradability assessments of methyl laurate in comparison with toluene and chloroform, respectively (Figure 4, Figure 5, and Figure 6). These assessments were performed using the *ChemFREE* web platform [86].

**Figure 7:**
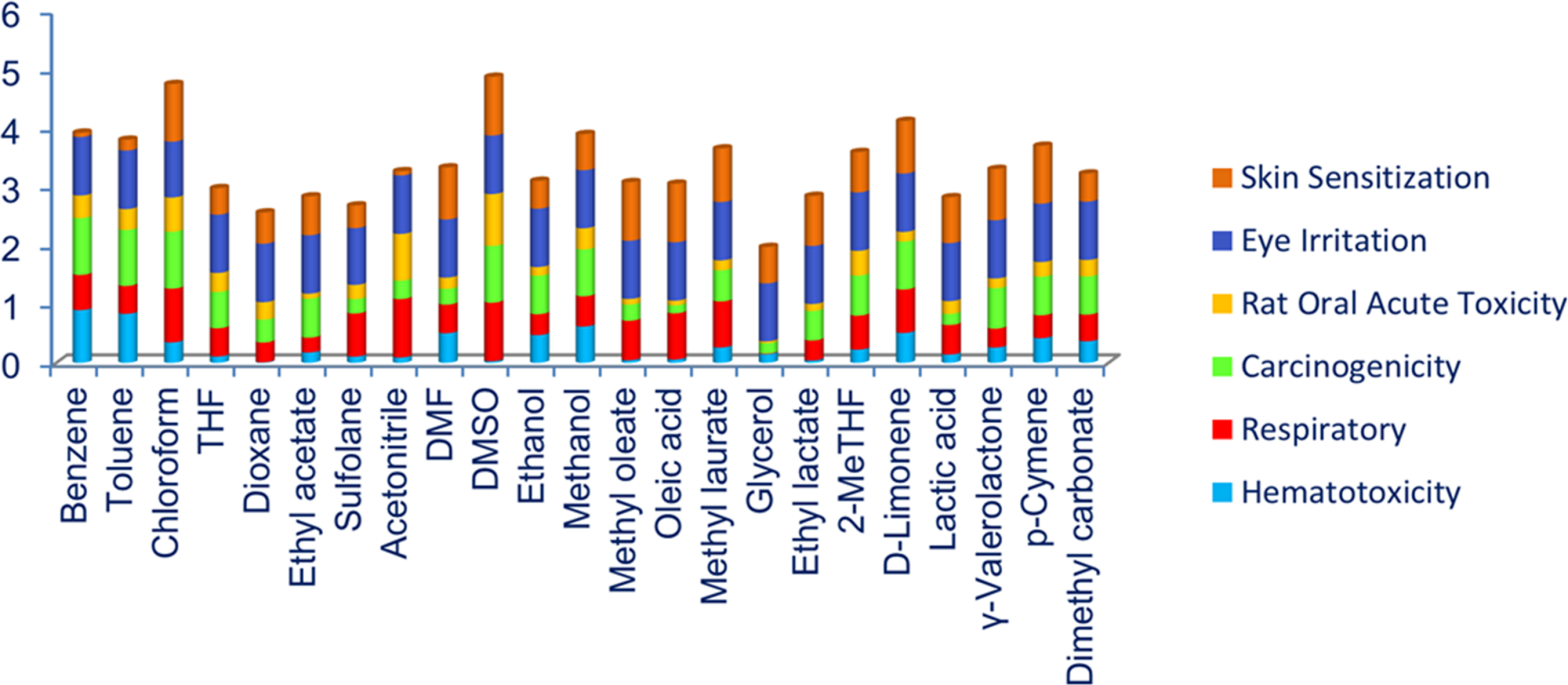
Various toxicity parameters of methyl laurate and a series of other solvents calculated by *ADMETLab 3.0*.

According to the ecological risk assessments for some species based on the data shown in these figures, methyl laurate does not pose a risk to 4 species, while chloroform and toluene do not pose a risk to 3 species (please see the green colored icons). In terms of environmental risk factors, the persistence of methyl laurate in soil, sediment and water, with the exception of air, is significantly lower than that of the other two solvents (values are calculated in days). In addition, it can be seen that out of these three solvents, only methyl laurate is readily biodegradable on the basis of environmental risk factors (in the OECD 301C modified MITI (I) test, a substance can be considered ready biodegradable if 60% of the substance is mineralized in 28 days (in terms of ThOD)). On the other hand, pesticide-likeness radar, "According to Hao's pesticide-likeness rule“ [93], the radar plot gives an integrated measure of six properties of chemicals, which performs more comprehensive qualitative analysis to exclude chemicals with properties most probably incompatible with an acceptable bioavailable profile. As the molecules of methyl laurate contain more rotatable bonds than the molecules of the other two solvents, this value was slightly higher in the pesticide-likeness radar. Moreover, molecular complexities, pesticide-likeness scores, RDL, GAU, QEX scores are quantitative assessment methods, which rely on some of the physicochemical properties are relevant, accessible, and easy to compute. A high score indicates a higher potential for chemicals to become new pesticides (the data obtained with *ChemFREE* for the other 13 solvents used in the study are given in Supporting Information File 1, Figure S1). In addition to the calculations already outlined, a series of toxicity properties of methyl laurate were calculated using *ADMETLab 3.0* in comparison with common organic solvents and some green solvents. The obtained results were visualized with a cylinder chart in Figure 7. Indeed, a significant proportion of the experimentally measured *Green Chemistry* parameters for methyl laurate are consistent with the predictions of various software or web platforms, and in some cases, even demonstrate that certain toxicity parameters of methyl laurate are more environmentally friendly. As demonstrated in the relevant literature, methyl laurate has been shown to be non-toxic to the acute oral route and to not induce mutagenic effects in the *S. typhimurium* reversion assay [94]. In the course of the experiments conducted with methyl laurate, no irritant effects on the skin of human subjects were detected. However, evidence of ocular irritation at very low levels was obtained. Furthermore, it has been determined that 100–69% of methyl laurate is readily biodegraded aerobically following a 30-day period. The utilization of certain fatty acid methyl esters, including but not limited to methyl laurate, has found application in a variety of direct and indirect food additive applications [94].

**Figure 8:**
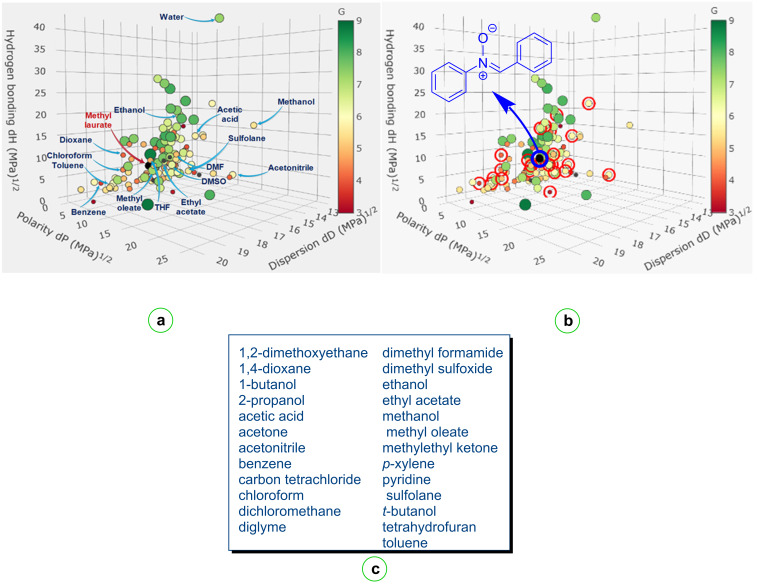
a) Visualization of the localization of conventional organic and bio-based solvents in the Hansen space. b) Position of the nitrone in the Hansen space. c) Organic solvents that can dissolve *C*,*N*-diphenylnitrone (experimentally determined).

The Hansen solubility parameter is a numerical expression that quantifies a molecule's cohesive energy density from non-polar, polar, and hydrogen-bonding interactions. It is a widely utilized tool in the field of molecular science for predicting the miscibility of solutes with solvents, as well as the miscibility of solutes within themselves, based on the fundamental principle of "*like dissolves like*" [95]. The location of the selected common organic solvents in the 3D Hansen space is shown in Figure 8a. The distribution of these selected solvents with other organic solvents in the 3D Hansen solubility space is determined by three Hansen solubility parameters (HSPs): dispersion (δ*_D_*), polarity (δ*_P_*), and hydrogen bonding (δ*_H_*). As is well established, information about their similarity in solubility properties can be obtained from the distance between solvents. The identification of problematic and non-problematic solvents is facilitated by the size and color of the specific sphere representing a particular solvent. As demonstrated by the figure, the position of methyl laurate (with values of δ*_D_* = 16, δ*_P_* = 2.1 and δ*_H_* = 5.3) can be found in the middle of the model spheres belonging to nonpolar and polar protic or polar aprotic solvents, which had previously been widely favored in cycloaddition reactions. These values reveal the similarity in solubility properties of the solvents. Furthermore, the evidence suggests that the majority of these solvents is hazardous and their utilization is to be avoided. As demonstrated in Figure 8a, the color of the spheres of the customary proportional solvents employed in [3 + 2] cycloadditions is red or yellow, thereby substantiating the potential hazard associated with their utilization as a solvent in the aforementioned reactions. For the solute component *C*,*N*-diphenylnitrone, the dispersion, polarity, and hydrogen bonding values for nitrone were determined as δ*_D_* = 16.6, δ*_P_* = 7.6 and δ*_H_* = 8.7. Figure 8b clearly shows the position of the solute in the Hansen space. The “Green Solvent Selection Tool“ was utilized to ascertain these three parameters [95]. For the purpose of this study, a series of solvents in which *C*,*N*-diphenylnitrone dissolves was determined through experimental means (at 22 °C) in our laboratory (Figure 8c). An experiment was conducted in which a series of organic solvents and water were subjected to solubility tests. For the purposes of this experiment, approximately 15 mg of nitrone was mixed with 1 mL of solvent in a 10 mL test tube at room temperature (22 °C). As demonstrated in Figure 8c, the solvents under consideration are all effective in facilitating the dissolution of nitrone. However, under identical conditions, the nitrone was not fully soluble in solvents such as water, diethyl ether, petroleum ether, triethylene glycol, diethanolamine, and glycerol. Then, these solvents were selected with the known functional solvent(s) of our solute icon in this tool and following the update, the parameter values given above for the relevant nitrone were readily obtained. As can be seen from the figure, methyl laurate is a good alternative to competing toxic organic solvents that are good solvents for this compound.

As well-known the Hildebrand solubility parameter (HSP, δ*_T_*) has been defined as a measure of the cohesive energy density of a material, thus facilitating prediction of the solubility of a solute in a solvent [96].


[1] $$\delta_{T}=\sqrt{\delta_{D}^{2}+\delta_{P}^{2}+\delta_{H}^{2}}$$

Methyl laurate has been found to have HSPs that are in close proximity to those that have been predicted for nitrone, with a δ*_T_* (the Hildebrand solubility parameter or the total Hildebrand parameter) of approximately 17. This finding indicates that methyl laurate is a suitable solvent for nitrone, which has a δ*_T_* of around 20. On the other hand, the radius of interaction (*R_a_*) is a critical factor in the assessment of solute solubility in a solvent, particularly in the context of HSPs [97–98]. It facilitates estimation of the extent of interaction between a solute and solvent, as determined by their respective solubility parameters. Consequently, the difference between the HSPs values can be utilized to calculate the *R_a_* value between the nitrone and methyl laurate. In order to calculate the *R_a_* value between the nitrone and methyl laurate, it is recommended to employ the following equation (*n =* nitrone, *ml* = methyl laurate):


[2] $$R_{a}=\sqrt{4\cdot {\left(\delta_{D}^{n}-\delta_{D}^{ml}\right)}^{2}+{\left(\delta_{P}^{n}-\delta_{P}^{ml}\right)}^{2}+{\left(\delta_{H}^{n}-\delta_{H}^{ml}\right)}^{2}}$$

According to Equation 2, *R_a_* was calculated to be approximately ≈ 6.57 MPa^1/2^. The radius of interaction (*R_o_*) for a typical small organic molecule, such as the nitrone, is estimated to be approximately 7.5 MPa^1/2^ which value is usually determined for solute molecules [97,99]. The relationship between *R_a_* and *R_o_* is denoted by the term relative energy difference (RED).


[3] $$\text{RED}=\frac{R_{a}}{R_{o}}$$

In the absence of any energy difference, the RED number is equivalent to 0. RED numbers that are less than 1 indicate high affinity, whilst RED numbers that are equal to or close to 1 represent a boundary circumstance. RED numbers that are progressively higher demonstrate increasingly lower affinities. Accordingly, when the values are substituted,


[4] $$\text{RED}=\frac{6.57}{7.5}\approx 0.876$$

It is evident that, given the RED value of approximately 0.876, which is less than 1, the nitrone is likely to dissolve in a methyl laurate solvent. Methyl laurate is a medium-sized molecule that exhibits a combination of polar and non-polar characteristics, arising from the ester group and the non-polar hydrocarbon chain, respectively. The nitrone contains a polar functional group (N–O) and two aromatic rings, which serve to generate both polar and dispersion forces. Consequently, these findings indicate that fatty acid methyl esters may possess considerable promise as environmentally friendly solvents for cycloaddition reactions. Moreover, as pointed out by Gil et al., the δ*_P_* and δ*_H_* values for methyl esters of saturated fatty acids decrease with increasing chain length [100]. Consequently, the capacity of methyl laurate molecules to direct electric charge and hydrogen bonding is greater than that of methyl esters with longer chains.

Despite the proposal of a number of laborious green production processes for certain of the organic solvents selected for consideration in this study, they are still commercially produced using fossil reserves through conventional processes [101–102]. It is imperative to ascertain not only the cost-effective production processes of green solvents but also their static permeability, polarizability, solubility, viscosity, diffusivity, thermal behaviour and volumetric, surface or critical physicochemical properties that determine their interaction with other molecules in their environment [103]. It is evident that the dipole moment, a physical parameter which is macroscopic in nature, can be evaluated in order to characterize solvent polarity. The value calculated for methyl laurate is shown in Table S1 in Supporting Informaiton File 1 and is in good agreement with the values of other organic solvents commonly used in cycloaddition reactions. It may thus be considered an ideal solvent for nitrones and *N*-substituted maleimides, which possess varying degrees of polarity. The observation that methyl laurate exhibits no propensity for hydrogen bonding between its own molecules, yet functions as an H-bonding acceptor for other molecules, has the potential to facilitate free volume in the cycloaddition reaction medium for suitable substrates. This may, in turn, result in a favourable enhancement of the reaction rate. As demonstrated in Supporting Information File 1, Table S1, entries 14–16, the polarizability of fatty acid derivatives is greater than that of other solvents, resulting in their enhanced dispersing power. Consequently, the attraction to dissolved molecules is also stronger.

**Figure 9:**
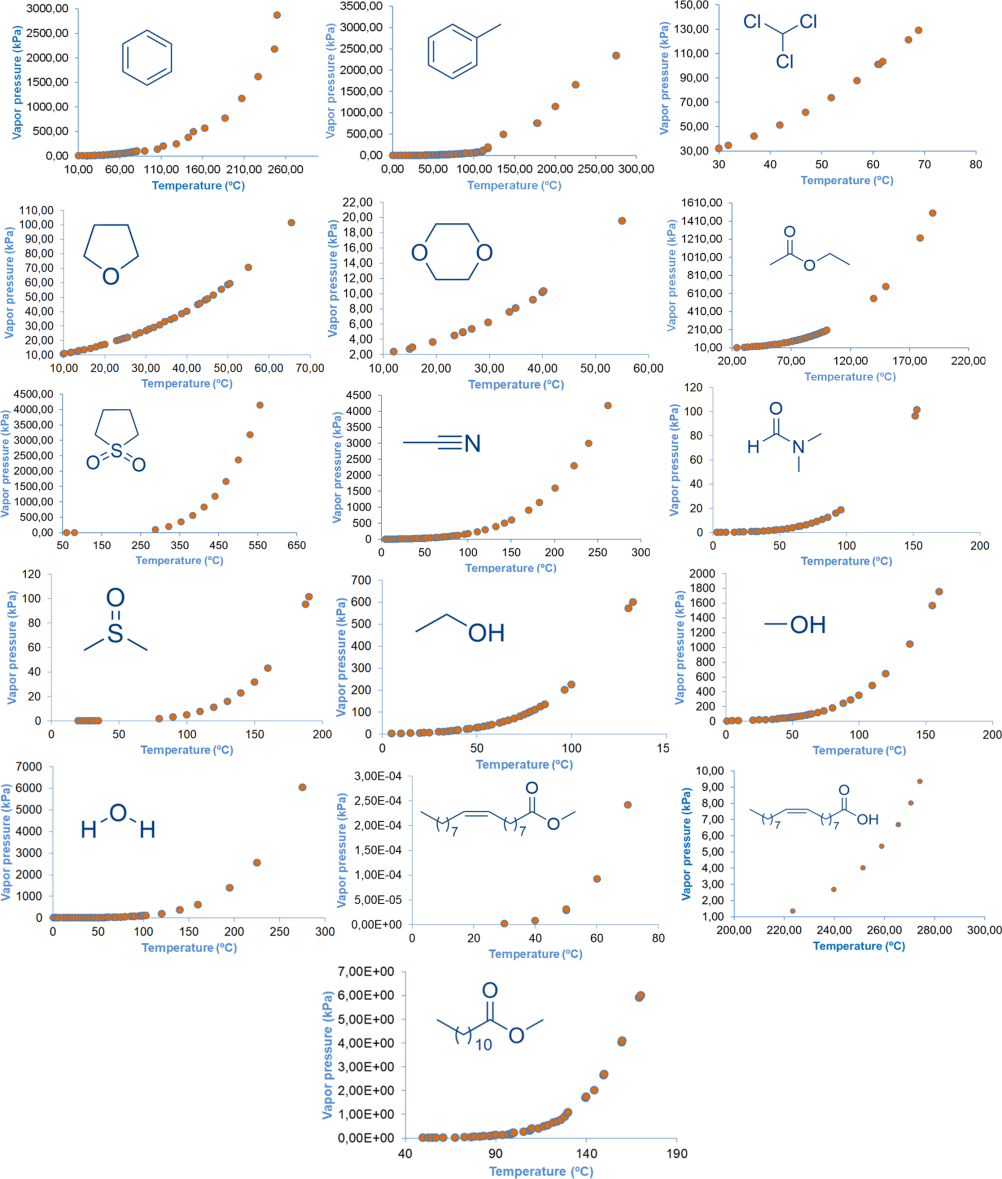
Vapour pressures of the solvents used (values retrieved from the *Chemeo* molecular database).

Gu and Jérôme proposed a set of 12 criteria (*availability*, *price*, *recyclability*, *grade*, *synthesis*, *toxicity*, *biodegradability*, *performance*, *stability*, *flammability*, *storage*, *renewability*) with a view to defining the concept of green solvents [71]. Methyl laurate, predicted as the main potential green solvent in this study, fulfils almost all of the above criteria. For example, it can be synthesized easily and with high efficiency through the biodiesel production process, it can be reused, it is stable under storage conditions, it can be obtained from renewable resources, it is biodegradable, non-flammable etc. It is widely acknowledged that conventional organic solvents present significant challenges, primarily due to the fact that many of these solvents are volatile organic compounds, due to their high vapour pressures. Consequently, the examination of the vapour pressures of the solvents to be selected for utilization in organic reactions provides significant insights into their volatility. As demonstrated in Figure 9, the vapour pressures of fatty acids and their derivatives are comparatively low when compared to other solvents. Table 3 presents a summary of the physicochemical parameters, biodegradabilities, and percentage yields of compound **3a** in each of the solvents that were considered in the current study. Furthermore, as indicated in Table 3, the vapour pressures of the solvents at a reaction temperature of 80 °C suggest their suitability to be used in cycloaddition processes, superseding the utilization of other volatile solvents. A subsequent examination of the yields obtained for the cycloaddition product **3a** in Table 3 demonstrates that, in accordance with the expectations, the reaction rate is slower in polar solvents. It is hypothesized that the slightly higher yields in water are probably due to hydrophobic effects [61]. Conversely, while the yields of cycloaddition reactions conducted in toluene and chloroform are satisfactory (Table 3), both solvents are classified as highly toxic. The European regulation regarding the 'Registration, Evaluation, Authorization and Restriction of Chemicals' (REACH) has led to limitations on the use of chlorinated solvents, toluene, DMF etc., with the implementation of particular prerequisites [89]. The double bond present in oleic acid and methyl oleate may be disadvantageous for these solvents in comparison to methyl laurate. The potential isomerization and reactivity of this double bond can pose significant challenges, particularly in cycloadditions or other organic transformations that necessitate extended cycloaddition processes and elevated temperatures.

**Table 3:** The physicochemical parameters, toxicities, and yields of **3a** obtained in the used solvents.

Solvent	Vapour pressure (kPa)^a^	Boiling point (°C)^a^	Flash point (°C)^a^	Hazard statement	Dipol moment (D)^b^	Atom economy (ref. [104]) (%)^c^	Biodegrad- ability^d^	Yield of **3a** (%)^e^

benzene	101.33	80	−12	highly flammable	0.00	–	non readily	54
toluene	38.87	111	4.4	highly flammable	0.27	–	non readily	84
chloroform	190.00	61	9.7	non flammable	1.35	–	non readily	74
THF	134.90	65	−21	highly flammable	1.71	80	readily	21
dioxane	64.54	101	12	highly flammable	0.45	–	readily	16
EtOAc	111.76	77	−4	highly flammable	1.89	96	readily	26
sulfolane	0.04	287	176	non flammable	4.50	–	readily	37
MeCN	96.34	82	5.56	highly flammable	3.22	–	readily	42
DMF	9.90	153	58	flammable	3.57	–	non readily	14
DMSO	2.00	189	87	non flammable	4.22	72	readily	34
EtOH	108.42	78	12	highly flammable	1.59	51	non readily	68
MeOH	182.61	64	12	highly flammable	1.54	100	non readily	34
water	47.37	100	–	non flammable	1.77	–	non readily	51
methyl oleate	0.0005	351	180	non flammable	1.74	–	readily	88
oleic acid	0.08	360	189	non flammable	1.69	–	readily	87
methyl laurate	0.06	262	134	non flammable	1.64	88	readily	100

^a^Values retrieved from the *Chemeo* molecular database (vapour pressure at 80 °C). ^b^Values calculated with Gaussian. ^c^Calculated value for the production of solvent. ^d^The relevant results were obtained with *ChemFREE* web platform. ^e^Yield obtained at the end of 10 minutes reaction time.

**Scheme 2:**
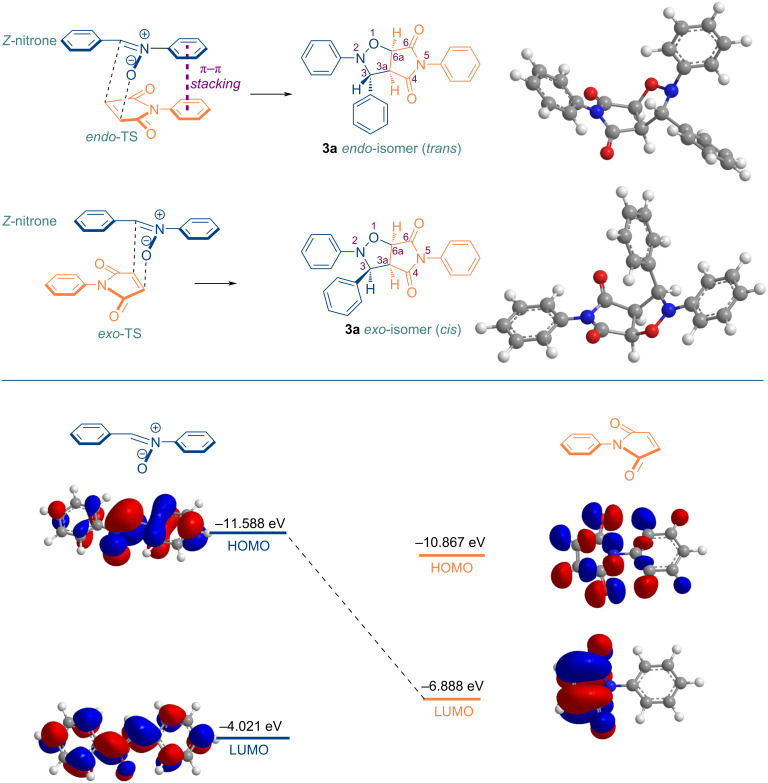
*Endo* and *exo* stereoisomeric approaches of nitrone **1a** and maleimide **2a** in [3 + 2] cycloaddition reaction.

**Figure 10:**
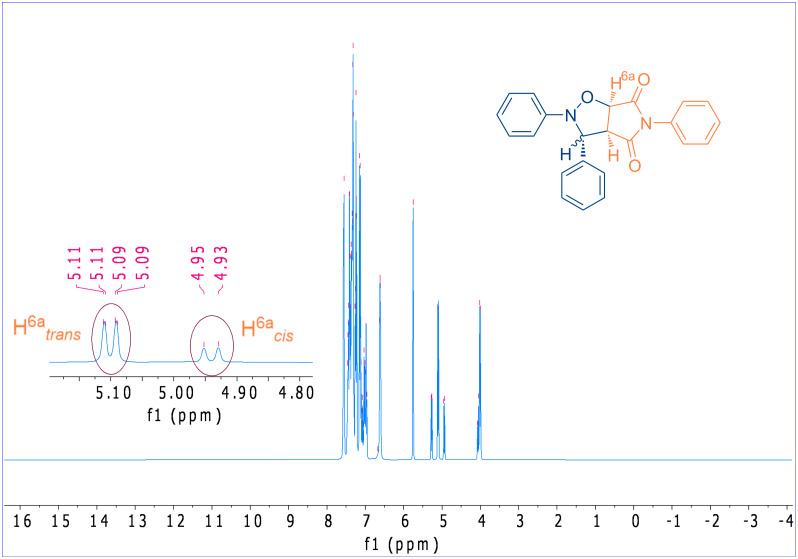
Signals of protons used in the calculation of the diastereomeric ratios (*cis*/*trans*) of cycloaddition products **3a**–**u**.

With a boiling point in excess of 260 °C, methyl laurate has the potential to function as a green solvent in a wide range of organic reactions that necessitate elevated temperatures. Furthermore, the oxidative stability index of methyl laurate at temperatures of 80 °C and 110 °C is greater than 40 (h), and the oxidation onset temperature is 198.5 °C which is better than those of methyl oleate [105]. In the case of castor oil, although all of the starting material was converted into the respective cycloadduct, the yield of the product was found to be lower than that observed in other solvents (Table 1, entry 6). This was primarily attributable to the utilization of a hexane/diethyl ether mixture in the isolation of the cycloadduct through precipitation, given that castor oil is insoluble in hexane. The solvent dissolves the product together with castor oil, causing it to pass to the filtrate phase and thereby reducing the amount present. In order to remain faithful to the principles of *Green Chemistry*, this study did not employ column chromatography for the isolation and purification of the products of interest from the reaction mixture. Otherwise, the yield of the reaction conducted in a castor oil medium would be similar to that of the other reactions. Furthermore, the rapid realization of the cycloaddition reaction in a non-polar solvent, such as methyl laurate, indicates that it proceeds via a non-polar mechanism. The lower polarity of the activated complex in comparison to the starting compounds supports the higher reaction rate in an non-polar solvent such as methyl laurate [61,106]. The 28:72 *exo/endo* (*cis/trans*) isomer ratio (Table 1, entry 9), obtained in this cycloaddition reaction indicates that the *endo* transition state is more stable, and that the thermodynamically more stable *trans* isomer is the major product in the reaction proceeding diastereoselectively through this transition state (Scheme 2). Meanwhile, the diastereomeric ratio of interest can be determined by integrating the ^1^H NMR spectra, in particular by selecting the appropriate signal pairs (one from each diastereomer) belonging to the respective cycloaddition product. An example of these selected protons (*trans*-H6a and *cis*-H6a) is shown in Figure 10 for the compound **3a**.

Conversely, when these two reagents were heated in toluene, the ratio of *cis/trans* diastereoisomers was observed to be 1:1 [107], indicating that a remarkable diastereoselectivity can be achieved when methyl laurate is used as a solvent. The predominance of the *trans* isomer can be attributed to the stabilizing of secondary orbital interactions in the *endo* transition state, which results in the formation of this product, in contrast to the *exo* transition state [53,108]. In this case, the most significant factor is the π–π interaction (π–π stacking) between the phenyl rings substituted on the nitrogen atoms of both maleimide and nitrone [109]. The most significant evidence confirming the interactions that determine the diastereoselectivity observed here is that the *cis* diastereomer is the major addition product in the dipolar cycloaddition reaction using *N*-methyl,*C*-phenylnitrone and *N*-methylmaleimide [110]. Furthermore, although mono- and bifunctional *N*-methylnitrones exhibit higher cycloaddition reaction rates than their *N*-phenyl analogues, their impact on selectivity is diminished [111]. It is well established that nitrones prepared from aromatic aldehydes exhibit a *Z*-configuration [112–113] and it appears that *endo*-coupling of the *Z*-configured *C*,*N*-diphenylnitrone with maleimide is more favorable. Indeed, the distribution of the *cis*/*trans* product is also found to be significantly influenced by the nature of the substituents present in the 1,3-dipole and/or dipolarophile and therefore, in some cases, *cis*-diastereoselectivity arises [85,114–117]. For instance, in the [3 + 2] cycloaddition reactions examined in earlier studies, intramolecular H-bonding, facilitated by the amide or alcohol functionality present in the nitrone structure, results in elevated *cis* or *trans* diastereoselectivity [85,111]. Furthermore, the [3 + 2] cycloaddition reactions of nitrones with electron-poor dipolarophiles, such as *N*-phenylmaleimide, is controlled by the HOMO FMO of the nitrone. Consequently, it can be deduced that HOMO_nitrone_–LUMO*_N_*_-phenylmaleimide_ interactions will be more pronounced in a non-polar methyl laurate environment (Scheme 2) [118].

In contrast, a limited number of studies have documented the occurrence of analogous cycloaddition reactions in water [62,108,113–121]. However, it should be noted that the isolation procedures employed in these studies to obtain the product from the aqueous reaction medium are time-consuming and inefficient. In this study, when water was used as a solvent, a similar problem was encountered to the one mentioned below, and the yield of the product was significantly reduced. The compounds employed in these studies are typically devoid of elongated hydrocarbon chains and in certain instances, bis-nitrone compounds have been employed as 1,3-dipoles. Furthermore, the reactions are known to be completed within approximately 3–4 or 20–32 hours at ambient temperature. Argyropoulou et al., obtained the cycloadduct products in relatively low yields (69%) from the reaction of hydrophobic nitrones with methyl acrylate over a 24-hour period in an aqueous suspension medium, in which hydrophobic effects are also involved [122]. In a further study conducted within an aqueous environment, it was possible to obtain the associated products resulting from the cycloaddition reactions of non-hydrophobic nitrones with ethyl cinnamate derivatives. These products were achieved with a yield of 78–85% within 12 hours, operating at 100 °C under conditions catalyzed by γ-cyclodextrin [123]. An attempt was made to synthesize compound **3a** in water under the reaction conditions specified in Table 1, entry 10. TLC analysis after 10 minutes revealed the presence of the starting nitrone in the reaction medium. The isolation of the product was achieved through extraction with EtOAc, followed by drying over anhydrous Na_2_SO_4_. The solvent was then evaporated, and the residue was triturated with hexane to precipitate the targeted product. However, at this stage, the product became excessively adhesive to the walls of the reaction vessel, thereby making it difficult to precipitate. Following the completion of these steps, the desired addition product was obtained in a yield of 51%. In the event of water being utilized as the solvent, the primary factor contributing to the low reaction yield over the specified timeframe is the low solubility of both reactants in water. Furthermore, it is acknowledged that the well-documented hydrogen bonding and hydrophobic effects do not exert a favorable influence on the reaction. This result suggests that methyl laurate may be a superior green solvent candidate for such reactions compared to water. Furthermore, it is evident that the initial compounds with a high degree of hydrophobic character may result in significant solubility issues within an aqueous environment.

**Figure 11:**
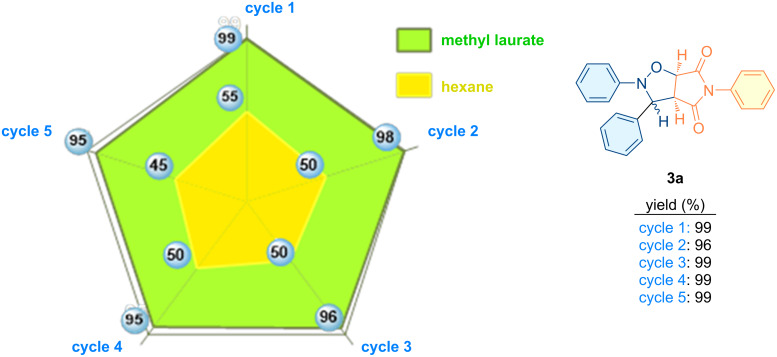
Results of studies on the recovery of solvents used in the reaction.

**Figure 12:**
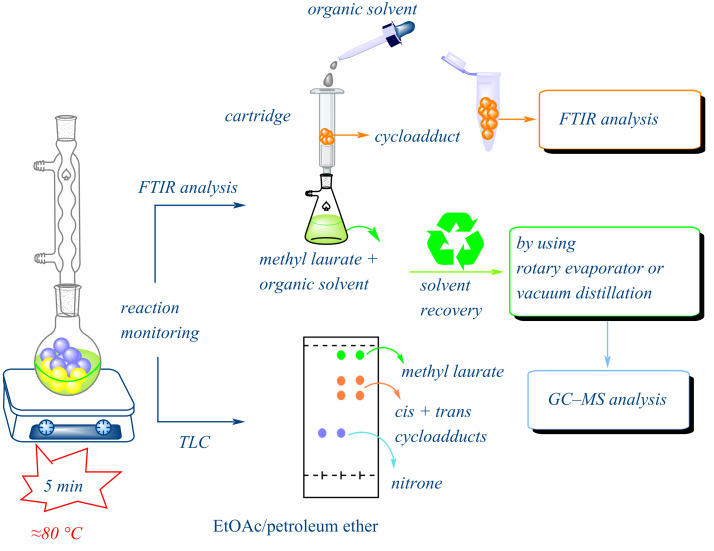
Simplified scheme describing the reaction monitoring and solvent recovery.

A series of studies were conducted with the objective of recovering and reusing the solvents utilized in the reaction and product isolation (Figure 11). To this end, a specific volume of hexane was introduced into the medium at the conclusion of the reaction, after which the product was precipitated and filtered under vacuum. The residue was then subjected to a subsequent wash with a volume of hexane, and the filtrate was transferred into a flask and concentrated at 45 °C using a rotary evaporator. The experimental conditions were such that 55% of the hexane was recovered, along with 99% of the methyl laurate. The methyl laurate that was recovered was then used as the reaction medium on four more occasions. The following report summarizes the findings of studies conducted on the recovery of solvents utilized in the aforementioned reaction (Figure 11). In addition, the completion of the cycloaddition reaction can be readily monitored through the utilization of Fourier-transform infrared (FTIR) spectroscopy and/or thin-layer chromatography (TLC) analysis (Figure 12). In order to verify the purity of the recovered methyl laurate at the conclusion of the reaction, a number of chromatographic (TLC), spectroscopic (FTIR) and GC–MS analyses were performed (Figures S2, S3, and S4 in Supporting Information File 1). Although a trace amount of impurity was observed on the TLC plate, the FTIR spectrum of the methyl laurate recovered after the reaction revealed that it had almost the same purity level as the methyl laurate before the reaction. This degree of purity is also clearly confirmed by the GC–MS spectrum of the recovered methyl laurate. However, if desired, the methyl laurate can be readily purified by vacuum distillation at the end of the reaction and safely reused.

The synthesis of the corresponding cycloaddition products **3a**–**u** was achieved by heating equimolar amounts of nitrone and maleimide in methyl laurate at 80 °C for the times indicated in Table 4. The corresponding products were readily isolated as a mixture of diastereoisomers by precipitation from the reaction medium with the addition of solvents such as hexane, octane or a diethyl ether/hexane mixture. As Welton asserts, the environmental friendliness of a chemical process is contingent upon the properties of the solvent, which must facilitate practical isolation of the product at the end of the process [124].

**Table 4:** Synthesized pyrrolo[3,4-*d*]isoxazolidines **3a**–**u** (*cis* + *trans* isomers).

Compound	Structure	Time (min)	*cis*/*trans* ratio	Yield (%)^a^

**3a**	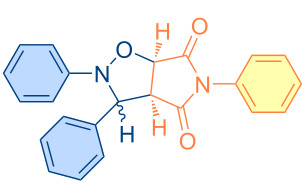	5	28:72	100
**3b**	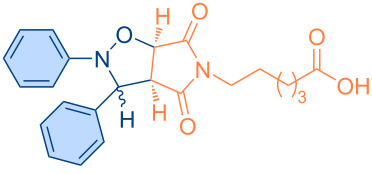	10	29:71	100
**3c**	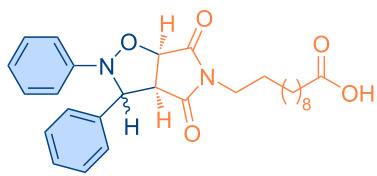	10	29:71	100
**3d**	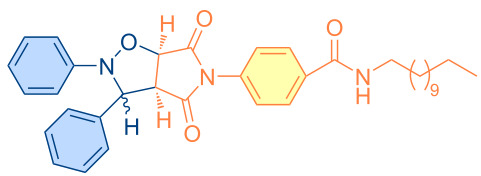	10	29:71	100
**3e**	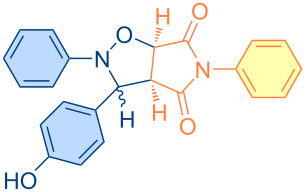	60	47:53	90
**3f**	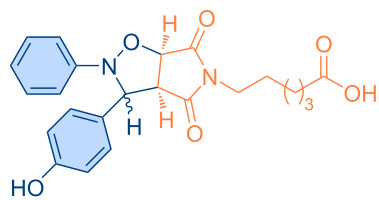	60	9:91	86
**3g**	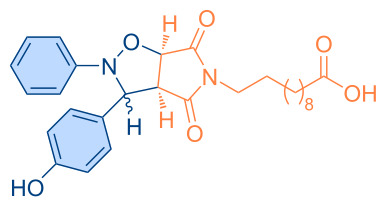	60	37:63	100
**3h**	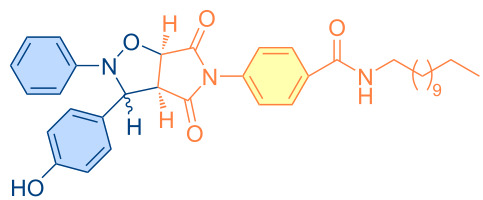	40	33:67	89
**3i**	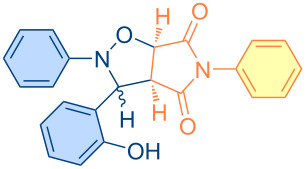	60	34:66	83
**3j**	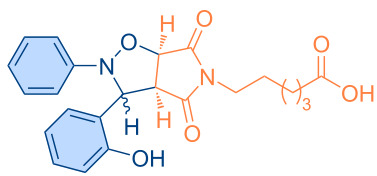	80	16:84	86
**3k**	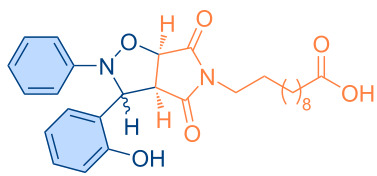	80	20:80	89
**3l**	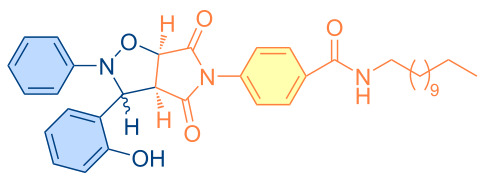	80	33:67	92
**3m**	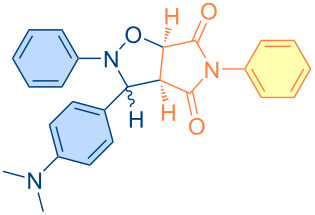	20	40:60	94
**3n**	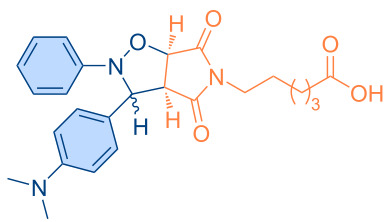	30	43:57	100
**3o**	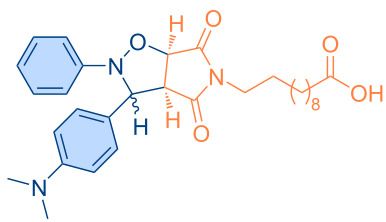	10	31:69	88
**3p**	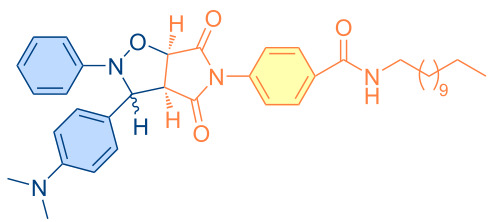	30	29:71	89
**3q**	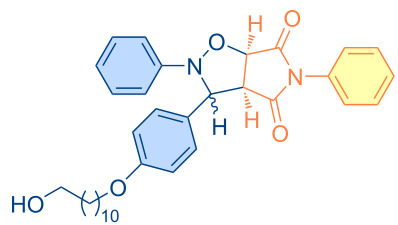	20	40:60	96
**3r**	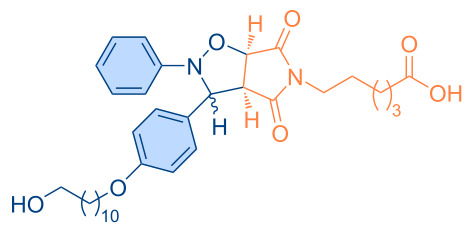	10	42:58	96
**3s**	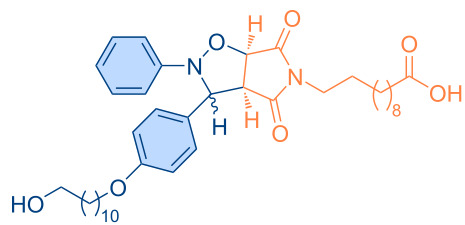	10	37:63	100
**3t**	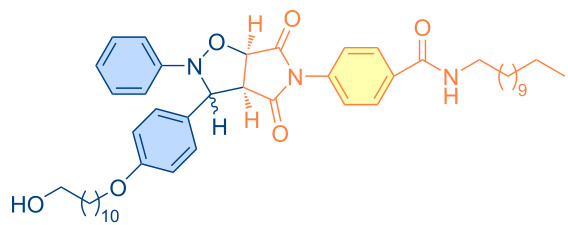	10	44:56	97
**3u**	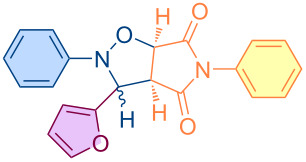	60	33:67	99

^a^Isolated yields.

**Figure 13:**
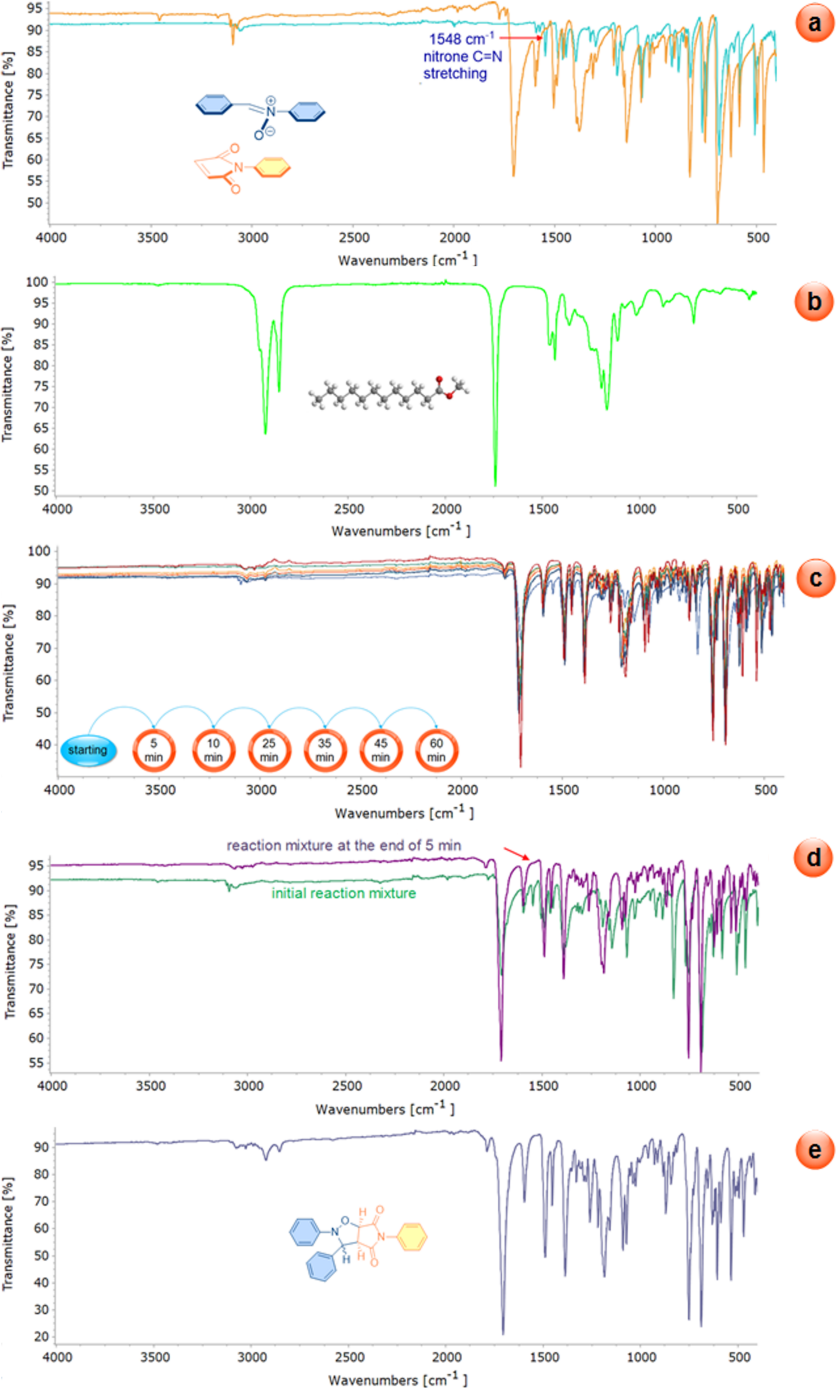
a) The superimposed spectra of *C*,*N*-diphenylnitrone and *N*-phenylmaleimide. b) The spectrum of methyl laurate. c) The spectrum of the reaction mixture, collected from the reaction medium after one hour had elapsed since the beginning of the reaction. d) The spectrum of the initial reaction mixture and the mixture collected from the reaction medium after five minutes. e) The spectrum of the isolated product **3a**.

As previously stated, the process of following the cycloaddition reactions within the scope of this study by means of FTIR spectroscopy is a relatively simple and practical task. The data obtained from the FTIR monitoring of the cycloaddition reaction between *C*,*N*-diphenylnitrone (**1a**) and *N*-phenylmaleimide (**2a**) carried out as a model reaction are given in Figure 13. As illustrated in Figure 13a, the superimposed spectra of *C*,*N*-diphenylnitrone and *N*-phenylmaleimide are presented. Figure 13b shows the spectrum of methyl laurate, which was utilized as a solvent. Figure 13c presents the spectrum of the reaction mixture, collected from the reaction medium after one hour had elapsed since the beginning of the reaction. Figure 13d shows the spectrum of the initial reaction mixture and the mixture collected from the reaction medium after five minutes. Figure 13e presents the spectrum of the isolated product **3a**. As demonstrated in Figure 13c and 13d, the signal attributed to the C=N stretching of starting nitrone **1a**, observed at 1548 cm^−1^, disappears. This finding suggests that the nitrone is fully consumed by the end of the five-minute reaction period.

In academic and industrial communities alike, there is a high level of interest in catalytic methods of *Green Chemistry* that allow stereoselective transformations [125–128]. It is imperative to develop stereoselective synthetic methods for cycloaddition reactions. In this study, a series of natural organic compounds from diverse classes and an acetonide [129] derivative were selected for investigation, with a focus on their potential catalytic effects in relation to the alteration of *cis/trans* product distribution under optimized [3 + 2] cycloaddition conditions. The primary strategy for the selection of these compounds as catalysts is predicated on their capacity to function as H-bond acceptors and/or H-bond donors. As has been documented in previous research, imine-based templates or [2]rotaxane that possess an amide functionality have been observed to exhibit notable *trans* diastereoselectivity in the context of cycloaddition reactions [130–135]. For this purpose, equivalent amounts of nitrone **1a** and maleimide **2a** were reacted with 5 mol % catalyst (Table 5) under the cycloaddition conditions determined in Table 1, entry 9. As illustrated in Table 5, the product yields obtained from the reactions are presented alongside the observed *cis/trans* diastereoisomer ratios in the presence of the respective catalysts. However, upon examination of the ^1^H NMR spectra of compound **3a** synthesized in the presence of the relevant catalysts, it was found that these catalyst molecules had no significant effect on the *cis/trans* diastereoisomer distribution.

**Table 5:** Catalyst survey for the synthesis of **3a** (*cis* + *trans* isomers).

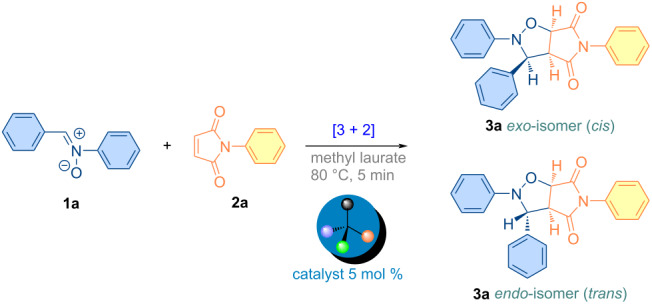			

Entry	Catalyst (5 mol %)	*cis*/*trans* ratio	Yield (%)^a^

1	*without catalyst*	28:72	100
2	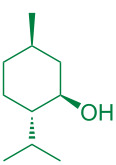 ᴅʟ-menthol	29:71	96
3	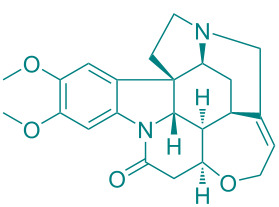 brucine	28:72	92
4	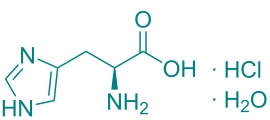 ʟ-histidine hydrochloride monohydrate	30:70	94
5	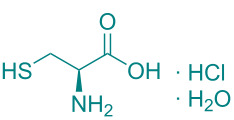 ʟ-cysteine hydrochloride monohydrate	29:71	96
6	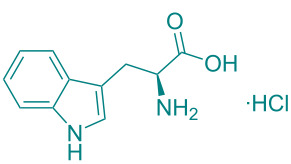 ʟ-tryptophan methyl ester hydrochloride	25:75	93
7	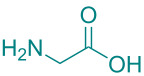 glycine	31:69	94
8	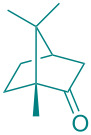 ᴅʟ-camphor	31:69	99
9	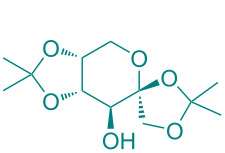 1,2;4,5-di-*O*-isopropylidene-β-ᴅ-fructopyranose	31:69	92
10	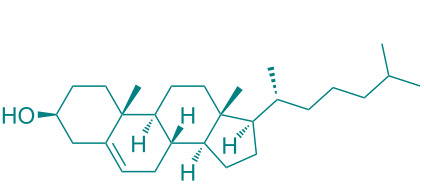 cholesterol	31:69	92

^a^Isolated yields.

## Conclusion

In conclusion, the present study reveals that methyl laurate is an excellent biocompatible solvent candidate for [3 + 2] cycloaddition reactions between nitrones and *N*-aryl-substituted maleimides. Methyl laurate meets most of the criteria for a readily available green solvent compared to the other 15 potential solvents compared. It is demonstrated that the toxicity prediction tools employed in the present study also indicate that methyl laurate is a more appropriate and eco-friendly solvent. Furthermore, a multitude of alternative vegetable oils could also be considered as green reaction media for such reactions. The pyrrolo-isoxazolidines targeted for synthesis in this *Green Chemistry* methodology were obtained as *cis/trans* diastereoisomer pairs in short reaction times and in very good yields. Despite the acknowledged fact that cycloaddition reactions remain unaffected by the solvent, this study demonstrates that the solvent can exert a substantial influence on the diastereomeric product distribution in such organic transformations. A series of studies were conducted on natural compounds selected for use as catalysts within the scope of this work. These studies revealed that there was no significant alteration to the ratio of diastereoisomers when the respective catalysts were utilized. In summary, methyl laurate is a solvent that has attracted attention as a potential alternative to conventional organic solvents on account of its environmentally friendly properties. The material is biodegradable, non-toxic and derived from renewable resources, thus making it a sustainable choice for a variety of applications. The primary benefit of this solvent lies in its reduced volatility and toxicity, characteristics that surpass many of the organic solvents evaluated in this study. Consequently, it presents itself as a promising reaction medium for [3 + 2] cycloaddition reactions, a process that conventional solvents may hinder due to their potential environmental or health implications.

## Experimental

All reagents and solvents were purchased from Merck (Merck, Darmstadt, Germany), Sigma-Aldrich (St. Louis, MO), or Acros Organics (Thermo Fisher Scientific, Geel, Belgium) and used without further purification. Thin-layer chromatography was performed using silica gel (60 F_254_, Merck, Darmstadt, Germany) plates. Melting points were recorded using a Büchi melting point B-540 apparatus (Büchi Labortechnik AG in Flawil, Switzerland). The IR spectra were measured by Spectrum Two FT-IR spectrometer (PerkinElmer, Massachusetts, USA). The NMR spectra were measured using Bruker ultrashield plus biospin 400 MHz NMR spectrometer and chloroform-*d* (CDCl_3_) or hexadeuterodimethyl sulfoxide (DMSO-*d*_6_) as a solvent. Chemical shifts (δ) are reported in ppm and *J* values in hertz. The elemental analyses were performed using a Leco CHNS-932 elemental analyzer (Saint Joseph, MI, USA). An Agilent 7890A gas chromatograph coupled with a 5975C mass spectrometer was used to analyze recovered methyl laurate. Chromatographic analysis of the methyl laurate dissolved in MeOH was performed in an HP-5MS fused silica capillary column (0.25 µm, 0.25 mm × 30 m). Helium (99.999%) was used as carrier gas with a constant flow rate of 1.0 mL min^−1^.

### Representative procedure for cycloaddition reactions

To a 50 mL single-necked flask, (0.1 g, 0.51 mmol) of *C*,*N*-diphenylnitrone, (0.09 g, 0.52 mmol) of *N*-phenylmaleimide and 1 mL of methyl laurate were added. The flask was attached to a reflux cooler and heated in an oil bath with stirring for a period of five minutes, until the internal temperature reached 80 °C. The reaction mixture rapidly precipitates to form a white solid product. The results of the FTIR and TLC analysis indicate that the starting compounds have been completely consumed. Subsequently, the reaction mixture was cooled to room temperature and hexane was added with rapid stirring, allowing the cycloaddition product to precipitate as a mixture of *cis* and *trans* diastereoisomers in the form of a white solid. The precipitate was then washed with a small quantity of hexane and dried in open air. The resulting mixture of isomers was subjected to direct NMR analysis, without any additional purification or separation steps.

## Supporting Information

File 1 Characterization data, copies of NMR spectra, additional Table and Figures.

## Data Availability

All data that supports the findings of this study is available in the published article and/or the supporting information of this article.
