# Supplementary material for: The high potential of methyl laurate as a recyclable competitor to conventional toxic solvents in [3 + 2] cycloaddition reactions
Source: Beilstein J Org Chem. 2025 Nov 5;21:2389–415. doi: 10.3762/bjoc.21.184 (PMC12599400; doi:10.3762/bjoc.21.184)
Supplement: File 1 — Characterization data, copies of NMR spectra, additional Table and Figures. [file Beilstein_J_Org_Chem-21-2389-s001.pdf]

BEILSTEIN JOURNAL OF ORGANIC CHEMISTRY

Supporting Information

for

The high potential of methyl laurate as a recyclable competitor to conventional toxic solvents in [3 + 2] cycloaddition reactions

Ayhan Yıldırım and Mustafa Göker

Beilstein J. Org. Chem. **2025,** *21,* 2389–2415. doi:10.3762/bjoc.21.184

Characterization data, copies of NMR spectra, additional Table and Figures

License and Terms: This is a supporting information file under the terms of the Creative Commons Attribution License (https://creativecommons.org/licenses/by/4.0). Please note that the reuse, redistribution and reproduction in particular requires that the author(s) and source are credited and that individual graphics may be subject to special legal provisions.
The license is subject to the *Beilstein Journal of Organic Chemistry* terms and conditions: (https://www.beilstein-journals.org/bjoc/terms)

Table of contents

1. Characterization data S2–S14
2. References S14
3. ^{1}H NMR spectra S15–S35
4. ^{13}C NMR spectra S36–S56
5. Figure S1a–m) Results for the 13 other solvents calculated by *ChemFREE* S57–S69
6. Table S1. Polarizabilities of the solvents S70–S71
7. Figure S2. FTIR spectra of recovered methyl laurate S72
8. Figure S3. TLC plate of recovered methyl laurate S73
9. Figure S4. GC chromatogram and MS spectrum of methyl laurate S74

*2,3,5-Triphenyltetrahydro-4H-pyrrolo[3,4-d]isoxazole-4,6(5H)-dione (**3a**)*[1]

White solid, mp 149–150 °C; ^{1}H NMR (400 MHz, $CDCl_3$), mixture of *cis/trans*-diastereoisomers (*28/72*): δ 7.56 (*d*, *J* = 8 Hz, 2H, Ar), 7.47-7.31 (*m*, 15H, Ar), 7.26-7.22 (*m*, 4H, Ar), 7.15-7.11 (*m*, 4H, Ar), 7.05-6.96 (*m*, 3H, Ar), 6.64-6.60 (*m*, 2H, Ar), 5.75 (*s*, 1H, *trans*-H^3), 5.27 (*d*, *J* = 7.8 Hz, 1H, *cis*-H^3), 5.10 (*d*, *J* = 7.6 Hz, 1H, *trans*-H^{6a}), 4.94 (*d*, *J* = 9.2 Hz, 1H, *cis*-H^{6a}), 4.05 (*t*, *J* = 9.2 Hz, 1H, *cis*-H^{3a}), 4.00 (*d*, *J* = 7.5 Hz, 1H, *trans*-H^{3a}); ^{13}C NMR (101 MHz, $CDCl_3$) δ 174.11, 173.34, 172.58, 171.28, 148.89, 147.44, 138.68, 134.56, 131.19, 130.99, 129.40, 129.13, 129.04, 129.00, 128.90, 128.85, 128.76, 128.18, 127.61, 126.56, 126.17, 125.98, 124.80, 122.94, 118.79, 114.46, 71.54, 70.07, 57.36, 54.67.

*6-(4,6-Dioxo-2,3-diphenylhexahydro-5H-pyrrolo[3,4-d]isoxazol-5-yl)hexanoic acid (**3b**)*

Oily amber liquid; ^{1}H NMR (400 MHz, $CDCl_3$), mixture of *cis/trans*-diastereoisomers (*29/71*): δ 7.49 (*d*, *J* = 7.3 Hz, 2H, Ar), 7.37 (*t*, *J* = 7.8 Hz, 2H, Ar), 7.32-7.28 (*m*, 3H, Ar), 7.24-7.17 (*m*, 3H, Ar), 7.09-7.02 (*m*, 4H, Ar), 6.92 (*t*, *J* = 7.4 Hz, 1H, Ar), 5.54 (*s*, 1H, *trans*-H^3), 5.08 (*d*, *J* = 7.8 Hz, 1H, *cis*-H^3), 4.98 (*d*, *J* = 7.4 Hz, 1H, *trans*-H^{6a}), 4.81 (*d*, *J* = 9.2 Hz, 1H, *cis*-H^{6a}), 3.90 (*t*, *J* = 9.2 Hz, 1H, *cis*-H^{3a}), 3.83 (*d*, *J* = 7.4 Hz, 1H, *trans*-H^{3a}); 3.20 (*t*, *J* = 7.5 Hz, 2H, -NC$\underline{H}_2$-), 2.26 (*t*, *J* = 7.5 Hz, 2H, -C$\underline{H}_2$COOH), 1.61 (*quin*, *J* = 7.4 Hz, 2H, -CH_2), 1.51 (*quin*, *J* = 6.8 Hz, 2H, -CH_2), 1.29-1.04 (*m*, 6H, -CH_2-); ^{13}C NMR (101 MHz, $CDCl_3$) δ 179.11, 175.06, 174.39, 173.60, 172.38, 170.86, 148.46, 147.31, 138.68, 134.48, 134.08, 129.06, 128.92, 128.83, 128.78, 128.75, 128.10, 127.52, 126.65, 124.82, 122.81, 119.04, 114.50, 71.11, 69.70, 57.24, 54.59, 39.02, 38.78, 37.61, 33.72, 33.65, 33.58, 28.17, 27.17, 26.25, 26.20, 26.15, 26.03, 24.10.

*11-(4,6-Dioxo-2,3-diphenylhexahydro-5H-pyrrolo[3,4-d]isoxazol-5-yl)undecanoic acid (**3c**)*

Oily amber liquid; ^{1}H NMR (400 MHz, $CDCl_3$), mixture of *cis/trans*-diastereoisomers (*29/71*): δ 7.48 (*d*, *J* = 7.6 Hz, 2H, Ar), 7.37 (*t*, *J* = 7.9 Hz, 2H, Ar), 7.32-7.28 (*m*, 4H, Ar), 7.23-7.16 (*m*, 4H, Ar), 7.09-7.01 (*m*, 4H, Ar), 6.90 (*t*, *J* = 7.5 Hz, 1H, Ar), 5.52 (*s*, 1H, *trans*-H^3), 5.07 (*d*, *J* = 7.8 Hz, 1H, *cis*-H^3), 4.98 (*d*, *J* = 7.5 Hz, 1H, *trans*-H^{6a}), 4.79 (*d*, *J* = 9.2 Hz, 1H, *cis*-H^{6a}), 3.89 (*t*, *J* = 9.2 Hz, 1H, *cis*-H^{3a}), 3.82 (*d*, *J* = 7.4 Hz, 1H, *trans*-H^{3a}); 3.19 (*t*, *J* = 7.5 Hz, 2H, -NC$\underline{H}_2$-), 2.34 (*t*, *J* = 7.5 Hz, 4H, 2x -C$\underline{H}_2$COOH), 1.66-1.55 (*m*, 4H, 2x -CH_2-), 1.41-1.01 (*m*, 26H, 13x -CH_2-); ^{13}C NMR (101 MHz, $CDCl_3$) δ 179.47, 175.08, 174.46, 173.63, 172.42, 170.92, 148.40, 147.30, 138.68, 134.49, 134.04, 129.00, 128.90, 128.81, 128.74, 128.71, 128.08, 127.54, 126.69, 124.81, 122.77, 119.14, 114.58, 71.15, 69.73, 57.24, 54.60, 39.35, 39.13, 37.94, 33.97, 29.36, 29.31, 29.25, 29.16, 29.02, 28.51, 27.56, 26.79, 26.71, 26.64, 24.68.

*4-(4,6-Dioxo-2,3-diphenylhexahydro-5H-pyrrolo[3,4-d]isoxazol-5-yl)-N-dodecylbenzamide (**3d**)*[2]

Yellowish solid, mp 146–147 °C; ^{1}H NMR (400 MHz, $CDCl_3$), mixture of *cis/trans*-diastereoisomers (*29/71*): δ 7.69 (*d*, *J* = 8.4 Hz, 2H, Ar), 7.56 (*d*, *J* = 7.8 Hz, 2H, Ar), 7.42 (*t*, *J* = 7.4 Hz, 3H, Ar), 7.38-7.34 (*m*, 3H, Ar), 7.23 (*t*, *J* = 7.5 Hz, 4H, Ar), 7.15-7.06 (*m*, 5H, Ar), 6.97 (*t*, *J* = 7.4 Hz, 3H, Ar), 6.69 (*d*, *J* = 8.4 Hz, 2H, Ar), 5.77 (*s*, 1H, *trans*-H^3), 5.29 (*d*, *J* = 7.8 Hz, 1H, *cis*-H^3), 5.12 (*d*, *J* = 7.5 Hz, 1H, *trans*-H^{6a}), 4.94 (*d*, *J* = 9.2 Hz, 1H, *cis*-H^{6a}), 4.07 (*t*, *J* = 8.8 Hz, 1H, *cis*-H^{3a}), 4.04 (*d*, *J* = 7.6 Hz, 1H, *trans*-H^{3a}), 3.45-3.39 (*m*, 4H, 2x -NHC$\underline{H}_2$-), 1.59 (*quin*, *J* = 7.3 Hz, 4H, 2x -NHCH_2C$\underline{H}_2$CH_2-), 1.36-1.26 (m, 36H, -CH_2-), 0.88 (*t*, *J* = 7 Hz, 6H, 2x -CH_3); ^{13}C NMR (101 MHz, $CDCl_3$) δ 173.82, 173.13, 172.28, 171.00, 169.04, 166.36, 148.82, 147.29, 138.50, 135.34,

135.07, 133.40, 129.42, 129.08, 129.02, 128.99, 128.87, 128.24, 127.68, 127.54, 126.52, 126.21, 125.91, 125.58, 124.92, 123.06, 118.85, 114.46, 71.57, 70.07, 57.37, 54.71, 40.23, 31.92, 29.65, 29.63, 29.60, 29.56, 29.47, 29.35, 29.33, 27.00, 22.69, 14.11.

*3-(4-Hydroxyphenyl)-2,5-diphenyltetrahydro-4H-pyrrolo[3,4-d]isoxazole-4,6(5H)-dione (**3e**)*[3]

Cream colored solid, mp 181–182 °C (dec); ^{1}H NMR (400 MHz, DMSO-d_6), mixture of *cis/trans*-diastereoisomers (*47/53*): δ 9.48 (*s*, 1H, Ar-OH), 9.47 (*s*, 1H, Ar-OH), 7.51-7.46 (*m*, 2H, Ar), 7.43-7.32 (*m*, 7H, Ar), 7.28-7.21 (*m*, 6H, Ar), 7.16-7.05 (*m*, 6H, Ar), 6.93 (*t*, *J* = 7.3 Hz, 1H, Ar), 6.77-6.71 (*m*, 4H, Ar), 6.67-6.65 (*m*, 2H, Ar), 5.68 (*s*, 1H, *trans*-H^3), 5.41 (*d*, *J* = 7.4 Hz, 1H, *cis*-H^3), 5.39 (*d*, *J* = 7.8 Hz, 1H, *trans*-H^{6a}), 4.87 (*d*, *J* = 9.2 Hz, 1H, *cis*-H^{6a}), 4.11 (*t*, *J* = 8.1 Hz, 1H, *cis*-H^{3a}), 4.05 (*d*, *J* = 7.4 Hz, 1H, *trans*-H^{3a}); ^{13}C NMR (101 MHz, DMSO-d_6) δ 174.63, 174.31, 173.38, 171.98, 157.22, 156.98, 148.89, 147.71, 131.54, 129.40, 128.95, 128.78, 128.66, 128.47, 128.37, 126.54, 126.40, 125.26, 124.48, 122.10, 119.13, 115.40, 115.20, 114.40, 77.66, 77.24, 70.46, 68.46, 56.54, 54.44.

*6-(3-(4-Hydroxyphenyl)-4,6-dioxo-2-phenylhexahydro-5H-pyrrolo[3,4-d]isoxazol-5-yl)hexanoic acid (**3f**)*

Sticky orange oil; ^{1}H NMR (400 MHz, $CDCl_3$), mixture of *cis/trans*-diastereoisomers (*9/91*): δ 7.32 (*d*, *J* = 8.7 Hz, 2H, Ar), 7.17 (*t*, *J* = 7.3 Hz, 2H, Ar), 7.00 (*d*, *J* = 7.8 Hz, 2H, Ar), 6.91 (*t*, *J* = 7.5 Hz, 2H, Ar), 6.81 (*d*, *J* = 8.5 Hz, 2H, Ar), 5.43 (*s*, 1H, *trans*-H^3), 5.10 (*d*, *J* = 7.9 Hz, 1H, *cis*-H^3), 4.99 (*d*, *J* = 7.4 Hz, 1H, *trans*-H^{6a}), 4.83 (*d*, *J* = 8.9 Hz, 1H, *cis*-H^{6a}), 3.87 (*t*, *J* = 8.7 Hz, 1H, *cis*-H^{3a}), 3.80 (*d*, *J* = 7.4 Hz, 1H, *trans*-H^{3a}), 3.24 (*t*, *J* = 6.6 Hz, 2H, -NCH_2), 2.26 (*t*, *J* = 7.4 Hz, 2H, -CH_2COOH), 1.64-1.12 (*m*,

12H, CH_2); ^{13}C NMR (101 MHz, $CDCl_3$) δ 177.90, 173.79, 155.50, 148.21, 130.67, 128.99, 128.23, 122.80, 115.68, 114.79, 69.34, 57.21, 38.98, 33.34, 26.25, 25.91, 24.06.

*11-(3-(4-Hydroxyphenyl)-4,6-dioxo-2-phenylhexahydro-5H-pyrrolo[3,4-d]isoxazol-5-yl)undecanoic acid (**3g**)*

Sticky orange oil; ^{1}H NMR (400 MHz, $CDCl_3$), mixture of *cis/trans*-diastereoisomers (37/*63*): δ 7.31 (*d*, *J* = 8.6 Hz, 2H, Ar), 7.23-7.15 (*m*, 4H, Ar), 7.08-7.03 (*m*, 2H, Ar), 6.99 (*d*, *J* = 8.0 Hz, 2H, Ar), 6.89 (*t*, *J* = 7.4 Hz, 1H, Ar), 6.80 (*d*, *J* = 8.6 Hz, 2H, Ar), 6.74 (*d*, *J* = 8.7 Hz, 1H, Ar), 5.42 (*s*, 1H, *trans*-H^3), 5.06 (*d*, *J* = 7.6 Hz, 1H, *cis*-H^3), 4.99 (*d*, *J* = 7.4 Hz, 1H, *trans*-H^{6a}), 4.71 (*d*, *J* = 8.9 Hz, 1H, *cis*-H^{6a}), 3.86 (*t*, *J* = 8.9 Hz, 1H, *cis*-H^{3a}), 3.79 (*d*, *J* = 7.4 Hz, 1H, *trans*-H^{3a}), 3.21 (*t*, *J* = 6.5 Hz, 2H, -NCH_2), 2.35 (*t*, *J* = 7.4 Hz, 2H, -CH_2COOH), 1.62 (*quin*, *J* = 7.4 Hz, 2H, CH_2), 1.34-1.09 (*m*, 38H, CH_2); ^{13}C NMR (101 MHz, $CDCl_3$) δ 178.64, 175.30, 174.77, 173.80, 173.01, 156.10, 155.51, 148.17, 147.24, 132.38, 130.64, 128.95, 128.90, 128.70, 128.25, 125.98, 124.90, 122.79, 119.37, 115.95, 115.82, 115.68, 114.85, 70.92, 69.38, 57.21, 54.53, 51.46, 39.35, 39.25, 34.16, 33.82, 33.75, 31.91, 29.59, 29.45, 29.32, 29.25, 29.20, 29.16, 28.98, 28.81, 27.58, 26.75, 26.70, 26.60, 24.98, 24.67, 24.63, 22.68.

*N-Dodecyl-4-(3-(4-hydroxyphenyl)-4,6-dioxo-2-phenylhexahydro-5H-pyrrolo[3,4-d]isoxazol-5-yl)benzamide (**3h**)*

Orange solid, mp 149–150 °C (dec); ^{1}H NMR (400 MHz, $CDCl_3$), mixture of *cis/trans*-diastereoisomers (33/*67*): δ 7.65 (*t*, *J* = 8.3 Hz, 4H, Ar), 7.31 (*d*, *J* = 8.4 Hz, 2H, Ar), 7.22-7.17 (*m*, 6H, Ar), 7.09-7.05 (*m*, 6H, Ar), 6.93 (*t*, *J* = 7.6 Hz, 2H, Ar), 6.82-6.75 (*m*, 4H, Ar), 6.66 (*d*, *J* = 8.3 Hz, 2H, Ar), 6.54 (*t*, *J* = 5.1 Hz, 1H, NH), 6.31 (*t*, *J* = 5.9 Hz, 1H, NH), 5.63 (*s*, 1H, *trans*-H^3), 5.23 (*d*, *J* = 7.8 Hz, 1H, *cis*-H^3), 5.08 (*d*, *J* = 7.5 Hz,

1H, *trans*-H^{6a}), 4.82 (*d*, *J* = 9.0 Hz, 1H, *cis*-H^{6a}), 3.97 (*t*, *J* = 8.9 Hz, 1H, *cis*-H^{3a}), 3.95 (*d*, *J* = 7.5 Hz, 1H, *trans*-H^{3a}), 3.43-3.38 (*m*, 4H, 2x -NCH_2), 1.59 (*s*, 4H, 2x CH_2), 1.33-1.25 (*m*, 36H, CH_2), 0.87 (*t*, *J* = 7.0 Hz, 6H, 2x CH_3); ^{13}C NMR (101 MHz, $CDCl_3$) δ 174.16, 173.59, 172.61, 171.53, 167.07, 166.81, 156.80, 156.07, 148.71, 147.40, 135.10, 134.75, 133.68, 133.51, 130.09, 129.35, 128.83, 127.98, 127.79, 127.58, 126.30, 126.02, 125.60, 124.86, 123.01, 118.85, 116.14, 115.86, 114.62, 71.35, 69.72, 57.30, 54.72, 40.43, 31.92, 29.64, 29.60, 29.57, 29.52, 29.35, 27.03, 27.00, 22.69, 14.11.

*3-(2-Hydroxyphenyl)-2,5-diphenyltetrahydro-4H-pyrrolo[3,4-d]isoxazole-4,6(5H)-dione **(3i)***[3]

Yellow solid, mp 186–187 °C (dec); ^1H NMR (400 MHz, DMSO-d_6), mixture of *cis/trans*-diastereoisomers (*34/66*): δ 8.06 (*s*, 1H, Ar-OH), 7.97 (*s*, 1H, Ar-OH), 7.52-7.42 (*m*, 4H, Ar), 7.39-7.15 (*m*, 18H, Ar), 7.07-7.03 (*m*, 1H, Ar), 6.98-6.94 (*m*, 1H, Ar), 6.92-6.85 (*m*, 2H, Ar), 6.65-6.62 (*m*, 2H, Ar), 5.91 (*s*, 1H, *trans*-H^3), 5.31 (*d*, *J* = 7.9 Hz, 1H, *cis*-H^3), 5.13 (*d*, *J* = 7.6 Hz, 1H, *trans*-H^{6a}), 4.96 (*d*, *J* = 9.2 Hz, 1H, *cis*-H^{6a}), 4.16 (*t*, *J* = 9.2 Hz, 1H, *cis*-H^{3a}), 4.15 (*d*, *J* = 7.6 Hz, 1H, *trans*-H^{3a}); ^{13}C NMR (101 MHz, DMSO-d_6) δ 174.28, 173.25, 172.07, 171.14, 155.20, 153.99, 150.21, 148.28, 145.94, 130.89, 130.52, 129.69, 129.51, 129.42, 129.18, 129.07, 129.01, 128.90, 128.84, 127.90, 126.55, 126.31, 126.11, 123.88, 123.18, 121.93, 121.00, 120.82, 120.25, 117.43, 116.77, 115.29, 68.16, 56.49, 53.52.

*6-(3-(2-Hydroxyphenyl)-4,6-dioxo-2-phenylhexahydro-5H-pyrrolo[3,4-d]isoxazol-5-yl)hexanoic acid (**3j**)*

Yellowish solid, mp 156–157 °C (dec); ^{1}H NMR (400 MHz, $CDCl_3$), mixture of *cis/trans*-diastereoisomers (*16/84*): δ 7.40 (*d*, *J* = 7.8 Hz, 1H, Ar), 7.24-7.21 (*m*, 3H, Ar), 7.11 (*d*, *J* = 7.6 Hz, 2H, Ar), 6.98 (*t*, *J* = 7.3 Hz, 1H, Ar), 6.94-6.89 (*m*, 2H, Ar), 5.70 (*s*, 1H, *trans*-H^3), 5.11 (*d*, *J* = 7.7 Hz, 1H, *cis*-H^3), 4.99 (*d*, *J* = 7.4 Hz, 1H, *trans*-H^{6a}), 4.89 (*d*, *J* = 9.4 Hz, 1H, *cis*-H^{6a}), 4.01 (*t*, *J* = 8.9 Hz, 1H, *cis*-H^{3a}), 3.96 (*d*, *J* = 7.5 Hz, 1H, *trans*-H^{3a}), 3.21 (t, *J* = 7.4 Hz, 2H, NCH_2), 2.26 (*t*, *J* = 7.4 Hz, 2H, -CH_2COOH), 1.65-1.09 (*m*, 6H, CH_2); ^{13}C NMR (101 MHz, $CDCl_3$) δ 172.85, 164.93, 154.04, 147.86, 134.08, 129.67, 129.18, 129.01, 128.03, 123.97, 120.80, 120.19, 116.91, 115.53, 70.34, 68.26, 56.57, 40.78, 38.98, 33.22, 29.69, 28.16, 26.28, 25.96, 24.04.

*11-(3-(2-Hydroxyphenyl)-4,6-dioxo-2-phenylhexahydro-5H-pyrrolo[3,4-d]isoxazol-5-yl)undecanoic acid (**3k**)*

Amber sticky oil; ^{1}H NMR (400 MHz, $CDCl_3$), mixture of *cis/trans*-diastereoisomers (*20/80*): δ 7.41 (*d*, *J* = 7.9 Hz, 1H, Ar), 7.24-7.17 (*m*, 3H, Ar), 7.10 (*d*, *J* = 8.3 Hz, 2H, Ar), 6.97-6.89 (*m*, 3H, Ar), 5.73 (*s*, 1H, *trans*-H^3), 5.09 (*d*, *J* = 7.7 Hz, 1H, *cis*-H^3), 4.98 (*d*, *J* = 7.4 Hz, 1H, *trans*-H^{6a}), 4.93 (*d*, *J* = 9.3 Hz, 1H, *cis*-H^{6a}), 4.03 (*t*, *J* = 9.3 Hz, 1H, *cis*-H^{3a}), 3.96 (*d*, *J* = 7.5 Hz, 1H, *trans*-H^{3a}), 3.18 (t, *J* = 7.3 Hz, 2H, NCH_2), 2.34 (*t*, *J* = 7.6 Hz, 2H, -CH_2COOH), 1.64-1.59 (*m*, 4H, CH_2), 1.26-1.08 (*m*, 26H, CH_2); ^{13}C NMR (101 MHz, $CDCl_3$) δ 179.38, 175.82, 174.48, 173.27, 172.96, 171.34, 154.86, 153.99, 148.20, 146.22, 134.06, 130.02, 129.48, 129.15, 128.91, 128.35, 127.81, 126.05, 123.82, 123.55, 120.69, 120.54, 120.18, 116.81, 116.40, 115.09, 67.34, 60.48, 56.43, 53.12, 39.46, 39.36, 37.96, 33.97, 31.90, 29.35, 29.27, 29.21, 29.14, 28.99, 28.50, 27.33, 26.83, 26.70, 26.62, 24.68, 21.04.

*N-Dodecyl-4-(3-(2-hydroxyphenyl)-4,6-dioxo-2-phenylhexahydro-5H-pyrrolo[3,4-d]isoxazol-5-yl)benzamide (**3l**)*

Yellowish solid, mp 182–183 °C (dec); ^{1}H NMR (400 MHz, DMSO-d_6), mixture of *cis/trans*-diastereoisomers (*33/67*): δ 10.06 (*s*, 2H, Ar-OH), 8.47 (*s*, 2H, C(O)NH), 7.94-7.77 (*m*, 4H, Ar), 7.40-6.85 (*m*, 20H, Ar), 6.64-6.62 (*m*, 2H, Ar), 5.93 (*s*, 1H, *trans*-H^3), 5.41 (*d*, *J* = 7.9 Hz, 1H, *cis*-H^3), 5.33 (*d*, *J* = 7.5 Hz, 1H, *trans*-H^{6a}), 5.08 (*d*, *J* = 8.9 Hz, 1H, *cis*-H^{6a}), 4.19 (*t*, *J* = 8.8 Hz, 1H, *cis*-H^{3a}), 4.13 (*d*, *J* = 7.6 Hz, 1H, *trans*-H^{3a}), 3.35 (*s*, 4H, 2x -NHC$\underline{H}_2$-), 1.50 (*s*, 4H, 2x -NHCH$_2$C$\underline{H}_2$CH$_2$-), 1.28-1.24 (m, 36H, -CH$_2$-), 0.85 (*t*, *J* = 7.2 Hz, 6H, 2x -CH$_3$); ^{13}C NMR (101 MHz, DMSO-d_6) δ 174.49, 173.09, 171.63, 165.31, 165.23, 154.84, 154.49, 149.72, 147.40, 134.86, 134.66, 133.95, 133.59, 129.17, 128.69, 127.76, 127.55, 127.16, 126.16, 126.04, 125.23, 124.71, 122.11, 121.44, 119.49, 119.07, 115.21, 113.70, 78.04, 77.13, 64.44, 62.61, 55.48, 52.86, 31.29, 29.04, 29.00, 28.77, 28.70, 26.46, 22.08, 13.92.

*3-(4-(Dimethylamino)phenyl)-2,5-diphenyltetrahydro-4H-pyrrolo[3,4-d]isoxazole-4,6(5H)-dione (**3m**)*[4]

Beige solid, mp 176–177 °C (dec); ^{1}H NMR (400 MHz, CDCl$_3$), mixture of *cis/trans*-diastereoisomers (*40/60*): δ 7.42-7.32 (*m*, 8H, Ar), 7.27-7.21 (*m*, 6H, Ar), 7.13-7.09 (*m*, 6H, Ar), 7.04 (*t*, *J* = 7.3 Hz, 1H, Ar), 6.96 (*t*, *J* = 7.3 Hz, 1H, Ar), 6.73 (*d*, *J* = 8.8 Hz, 2H, Ar), 6.68-6.64 (*m*, 4H, Ar), 5.65 (*s*, 1H, *trans*-H^3), 5.24 (*d*, *J* = 7.8 Hz, 1H, *cis*-H^3), 5.12 (*d*, *J* = 7.5 Hz, 1H, *trans*-H^{6a}), 4.83 (*d*, *J* = 9.2 Hz, 1H, *cis*-H^{6a}), 4.05 (*t*, *J* = 9.2 Hz, 1H, *cis*-H^{3a}), 3.98 (*d*, *J* = 7.5 Hz, 1H, *trans*-H^{3a}), 2.96 (*s*, 6H, 2x -C$\underline{H}_3$), 2.93 (*s*, 6H, 2x -C$\underline{H}_3$); ^{13}C NMR (101 MHz, CDCl$_3$) δ 174.39, 173.78, 172.86, 171.66, 150.65, 150.35, 148.98, 147.83, 131.38, 131.09, 129.27, 129.08, 128.98, 128.91, 128.72, 128.65,

128.42, 127.49, 126.22, 126.12, 126.04, 124.52, 122.69, 121.20, 118.85, 114.65, 112.57, 112.50, 71.68, 69.91, 57.33, 54.70, 40.50, 40.33.

*6-(3-(4-(Dimethylamino)phenyl)-4,6-dioxo-2-phenylhexahydro-5H-pyrrolo[3,4-d]isoxazol-5-yl)hexanoic acid (**3n**)*

Orange sticky liquid; ^{1}H NMR (400 MHz, $CDCl_3$), mixture of *cis/trans*-diastereoisomers (*43/57*): δ 7.30 (*d*, *J* = 8.9 Hz, 2H, Ar), 7.22-7.23 (*m*, 6H, Ar), 7.08 (*d*, *J* = 7.6 Hz, 2H, Ar), 7.04-7.00 (*m*, 3H, Ar), 6.89 (*t*, *J* = 7.3 Hz, 1H, Ar), 6.70-6.63 (*m*, 4H, Ar), 5.42 (*s*, 1H, *trans*-H^3), 5.05 (*d*, *J* = 7.8 Hz, 1H, *cis*-H^3), 4.98 (*d*, *J* = 7.4 Hz, 1H, *trans*-H^{6a}), 4.72 (*d*, *J* = 8.9 Hz, 1H, *cis*-H^{6a}), 3.84 (*d*, *J* = 7.6 Hz, 1H, *trans*-H^{3a}), 3.81 (*t*, *J* = 8.9 Hz, 1H, *cis*-H^{3a}), 3.22 (*t*, *J* = 6.6 Hz, 2H, -NCH_2-), 2.93 (*s*, 6H, 2x -$C\underline{H}_3$), 2.92 (*s*, 6H, 2x -$C\underline{H}_3$), 2.31 (*t*, *J* = 7.5 Hz, 2H, -$C\underline{H}_2$COOH), 2.26 (*t*, *J* = 7.5 Hz, 2H, -$C\underline{H}_2$COOH), 1.65-1.48 (*m*, 6H, CH_2), 1.34-1.26 (*m*, 6H, CH_2); ^{13}C NMR (101 MHz, $CDCl_3$) δ 173.82, 173.13, 172.28, 171.00, 169.04, 166.36, 148.82, 147.29, 138.50, 135.34, 129.42, 129.02, 128.87, 128.24, 127.68, 127.54, 126.52, 126.21, 125.91, 123.06, 118.85, 114.46, 71.57, 70.07, 57.37, 54.71, 40.23, 31.92, 29.65, 29.63, 29.60, 29.56, 29.35, 29.33, 27.00, 22.69, 14.11.

*11-(3-(4-(Dimethylamino)phenyl)-4,6-dioxo-2-phenylhexahydro-5H-pyrrolo[3,4-d]isoxazol-5-yl)undecanoic acid (**3o**)*

Orange sticky liquid; ^{1}H NMR (400 MHz, $CDCl_3$), mixture of *cis/trans*-diastereoisomers (*31/69*): δ 7.30 (*d*, *J* = 8.8 Hz, 2H, Ar), 7.21-7.12 (*m*, 5H, Ar), 7.09-7.04 (*m*, 2H, Ar), 6.88 (*t*, *J* = 7.5 Hz, 1H, Ar), 6.69 (*d*, *J* = 8.8 Hz, 2H, Ar), 6.63 (*d*, *J* = 8.8 Hz, 1H, Ar), 5.52 (*s*, 1H, *trans*-H^3), 5.04 (*d*, *J* = 7.6 Hz, 1H, *cis*-H^3), 4.98 (*d*, *J* = 7.4 Hz, 1H, *trans*-H^{6a}), 4.68 (*d*, *J* = 9.0 Hz, 1H, *cis*-H^{6a}), 3.83 (*d*, *J* = 7.6 Hz, 1H, *trans*-H^{3a}), 3.79 (*t*, *J* = 9.0 Hz, 1H, *cis*-H^{3a}), 3.23-3.19 (*m*, 2H, -NCH_2-), 2.93 (*s*, 12H, 4x -$C\underline{H}_3$); 2.36-2.32 (*m*,

4H, 2x -C$\underline{H}_2$COOH), 1.66-1.58 (*m*, 4H, CH_2), 1.28-1.09 (*m*, 28H, CH_2); ^{13}C NMR (101 MHz, $CDCl_3$) δ 179.36, 175.39, 174.85, 173.90, 172.77, 150.44, 150.25, 148.44, 147.72, 128.87, 128.62, 128.35, 127.66, 126.11, 124.51, 122.53, 121.24, 119.12, 114.86, 112.59, 112.31, 71.29, 69.55, 57.22, 54.55, 40.51, 40.29, 39.27, 39.10, 33.97, 29.35, 29.31, 29.26, 29.17, 29.11, 29.02, 27.63, 26.85, 26.74, 26.65, 24.69.

*4-(3-(4-(Dimethylamino)phenyl)-4,6-dioxo-2-phenylhexahydro-5H-pyrrolo[3,4-d]isoxazol-5-yl)-N-dodecylbenzamide (**3p**)*

Yellow solid, mp 147–148 °C (dec); ^{1}H NMR (400 MHz, $CDCl_3$), mixture of *cis/trans*-diastereoisomers (*29/71*): δ 7.74 (*d*, *J* = 8.5 Hz, 3H, Ar), 7.67 (*d*, *J* = 8.5 Hz, 2H, Ar), 7.37 (*d*, *J* = 8.8 Hz, 3H, Ar), 7.23-7.10 (*m*, 11H, Ar), 6.74-6.69 (*m*, 4H, Ar), 6.64 (*d*, *J* = 8.8 Hz, 3H, Ar), 6.25 (*t*, *J* = 5.6 Hz, 1H, NH), 6.20 (*t*, *J* = 5.6 Hz, 1H, NH), 5.65 (*s*, 1H, *trans*-H^{3}), 5.24 (*d*, *J* = 7.8 Hz, 1H, *cis*-H^{3}), 5.11 (*d*, *J* = 7.5 Hz, 1H, *trans*-H^{6a}), 4.79 (*d*, *J* = 9.0 Hz, 1H, *cis*-H^{6a}), 3.99 (*d*, *J* = 7.5 Hz, 1H, *trans*-H^{3a}), 3.97 (*t*, *J* = 9.0 Hz, 1H, *cis*-H^{3a}), 3.44-3.38 (*m*, 4H, 2x -NCH_2-), 2.96 (*s*, 6H, 2x -C$\underline{H}_3$), 2.92 (*s*, 6H, 2x -C$\underline{H}_3$), 1.61-1.56 (*m*, 6H, CH_2), 1.37-1.21 (*m*, 32H, CH_2), 0.88 (*t*, *J* = 7.0 Hz, 6H, 2x CH_3); ^{13}C NMR (101 MHz, $CDCl_3$) δ 174.13, 173.65, 172.59, 171.42, 166.51, 166.42, 150.65, 150.36, 148.91, 147.69, 135.23, 134.95, 133.80, 133.50, 129.29, 128.73, 128.34, 127.67, 127.53, 127.47, 126.23, 126.03, 124.63, 122.80, 120.96, 118.94, 114.65, 112.57, 112.44, 71.71, 69.93, 57.32, 54.77, 40.49, 40.29, 40.24, 31.93, 29.66, 29.64, 29.61, 29.58, 29.35, 27.03, 22.69, 14.11.

*3-(4-((11-Hydroxyundecyl)oxy)phenyl)-2,5-diphenyltetrahydro-4H-pyrrolo[3,4-d]isoxazole-4,6(5H)-dione (**3q**)*[2]

White solid, mp 84–85 °C; ^{1}H NMR (400 MHz, $CDCl_3$), mixture of *cis/trans*-diastereoisomers (*40/60*): δ 7.45-7.32 (*m*, 11H, Ar), 7.26-7.22 (*m*, 5H, Ar), 7.14-7.05 (*m*, 6H, Ar), 6.97 (*t*, *J* = 8.8 Hz, 1H, Ar), 6.91 (*d*, *J* = 8.8 Hz, 2H, Ar), 6.87 (*d*, *J* = 8.6 Hz, 1H, Ar), 6.65-6.63 (*m*, 2H, Ar), 5.68 (*s*, 1H, *trans*-H^3), 5.26 (*d*, *J* = 7.9 Hz, 1H, *cis*-H^3), 5.12 (*d*, *J* = 7.5 Hz, 1H, *trans*-H^{6a}), 4.87 (*d*, *J* = 9.2 Hz, 1H, *cis*-H^{6a}), 4.02 (*t*, *J* = 9.2 Hz, 1H, *cis*-H^{3a}), 3.97 (*d*, *J* = 7.5 Hz, 1H, *trans*-H^{3a}), 3.94-3.90 (*m*, 4H, 2x -C$\underline{H}_2$OAr), 3.63 (*t*, *J* = 6.6 Hz, 4H, 2x -C$\underline{H}_2$OH), 1.81-1.72 (*m*, 4H, 2x -C$\underline{H}_2$-), 1.56 (*quin*, *J* = 6.5 Hz, 6H, 3x -C$\underline{H}_2$-), 1.47-1.42 (m, 6H, 3x -C$\underline{H}_2$-), 1.32-1.26 (m, 20H, 10x -C$\underline{H}_2$-); ^{13}C NMR (101 MHz, $CDCl_3$) δ 174.21, 173.56, 172.67, 171.49, 159.50, 159.03, 148.81, 147.49, 131.25, 131.01, 130.42, 129.33, 129.14, 129.00, 128.96, 128.77, 128.74, 127.80, 126.18, 126.00, 125.96, 124.76, 122.87, 118.95, 114.95, 114.88, 114.59, 71.30, 69.73, 68.16, 68.00, 63.09, 57.37, 54.63, 32.83, 29.58, 29.53, 29.50, 29.42, 29.37, 29.24, 26.06, 26.03, 25.76.

*6-(3-(4-((11-Hydroxyundecyl)oxy)phenyl)-4,6-dioxo-2-phenylhexahydro-5H-pyrrolo[3,4-d]isoxazol-5-yl)hexanoic acid (**3r**)*

Amber sticky liquid, ^{1}H NMR (400 MHz, $CDCl_3$), mixture of *cis/trans*-diastereoisomers (*42/58*): δ 7.37-7.34 (*m*, 2H, Ar), 7.23-7.16 (m, 6H, Ar), 7.08-7.00 (m, 4H, Ar), 6.92-6.82 (m, 6H, Ar), 5.45 (*s*, 1H, *trans*-H^3), 5.07 (*d*, *J* = 7.6 Hz, 1H, *cis*-H^3), 4.99 (*d*, *J* = 7.4 Hz, 1H, *trans*-H^{6a}), 4.74 (*d*, *J* = 9.0 Hz, 1H, *cis*-H^{6a}), 3.93 (*t*, *J* = 6.9 Hz, 2H, -CH_2OAr), 3.85 (*t*, *J* = 9.2 Hz, 1H, *cis*-H^{3a}), 3.79 (*d*, *J* = 7.4 Hz, 1H, *trans*-H^{3a}), 3.66 (*t*, *J* = 2.1 Hz, 1H, -OH), 3.64 (*t*, *J* = 6.6 Hz, 2H, -CH_2OH), 3.22 (*t*, *J* = 6.5 Hz, 2H, -NCH_2), 2.32 (*t*, *J* = 7.5 Hz, 2H, -CH_2COOH), 2.26 (*t*, *J* = 7.5 Hz, 2H, -CH_2COOH), 1.76 (*quin*,

J = 6.3 Hz, 4H, CH_2), 1.63-1.07 (*m*, 44H, CH_2); ^{13}C NMR (101 MHz, $CDCl_3$) δ 178.36, 178.09, 175.17, 174.56, 173.70, 172.55, 159.36, 158.94, 148.35, 147.43, 130.39, 128.99, 128.72, 128.65, 127.91, 125.90, 124.75, 122.73, 119.09, 114.80, 114.71, 70.90, 69.36, 68.11, 67.95, 63.07, 57.24, 54.52, 51.43, 38.99, 38.79, 34.15, 33.61, 33.52, 32.75, 32.67, 31.91, 29.60, 29.56, 29.52, 29.48, 29.40, 29.34, 29.25, 29.22, 29.16, 27.25, 26.31, 26.27, 26.04, 25.74, 25.68, 24.98, 24.13, 22.68.

*11-(3-(4-((11-Hydroxyundecyl)oxy)phenyl)-4,6-dioxo-2-phenylhexahydro-5H-pyrrolo[3,4-d]isoxazol-5-yl)undecanoic acid (**3s**)*

Yellowish sticky liquid, ^{1}H NMR (400 MHz, $CDCl_3$), mixture of *cis/trans*-diastereoisomers (*37/63*): δ 7.35 (*d*, *J* = 8.6 Hz, 2H, Ar), 7.23-7.15 (m, 4H, Ar), 7.08-6.99 (m, 4H, Ar), 6.91-6.81 (m, 4H, Ar), 5.43 (*s*, 1H, *trans*-H^{3}), 5.06 (*d*, *J* = 7.6 Hz, 1H, *cis*-H^{3}), 4.99 (*d*, *J* = 7.4 Hz, 1H, *trans*-H^{6a}), 4.73 (*d*, *J* = 9.1 Hz, 1H, *cis*-H^{6a}), 3.93 (*t*, *J* = 7.4 Hz, 2H, -CH_2OAr), 3.85 (*t*, *J* = 9.2 Hz, 1H, *cis*-H^{3a}), 3.79 (*d*, *J* = 7.4 Hz, 1H, *trans*-H^{3a}), 3.65 (*t*, *J* = 2.1 Hz, 1H, -OH), 3.64 (*t*, 2H, *J* = 6.6 Hz, -CH_2OH), 3.22-3.19 (*m*, 2H, -NCH_2), 2.35-2.28 (m, 4H 2x -CH_2COOH), 1.76 (*quin*, *J* = 7.3 Hz, 4H, CH_2), 1.64-1.08 (*m*, 56H, CH_2); ^{13}C NMR (101 MHz, $CDCl_3$) δ 178.89, 178.67, 175.20, 174.60, 173.73, 172.59, 159.33, 158.93, 148.29, 147.45, 130.39, 128.93, 128.69, 127.96, 125.92, 124.72, 122.70, 119.12, 114.78, 114.69, 70.93, 69.39, 68.10, 67.93, 63.05, 57.23, 54.52, 39.31, 39.12, 33.94, 32.75, 29.60, 29.56, 29.52, 29.48, 29.41, 29.38, 29.35, 29.31, 29.25, 29.22, 29.20, 29.16, 29.08, 29.05, 29.02, 27.62, 26.84, 26.71, 26.63, 26.08, 26.01, 25.74, 24.98, 24.70.

N-Dodecyl-4-(3-(4-((11-hydroxyundecyl)oxy)phenyl)-4,6-dioxo-2-phenylhexahydro-5H-pyrrolo[3,4-d]isoxazol-5-yl)benzamide (***3t***)[2]

Yellowish solid, mp 108–109 °C; ^{1}H NMR (400 MHz, $CDCl_3$), mixture of *cis/trans*-diastereoisomers (*44/56*): δ 7.77-775 (*m*, 2H, Ar), 7.69-7.67 (m, 2H, Ar), 7.44-7.42 (m, 3H, Ar), 7.31-7.29 (m, 3H, Ar), 7.25-7.16 (m, 4H, Ar), 7.12-7.05 (m, 4H, Ar), 6.97-6.96 (m, 2H, Ar), 6.92-6.90 (m, 2H, Ar), 6.86-6.84 (m, 2H, Ar), 6.71-6.69 (m, 2H, Ar), 6.25 (*t*, *J* = 5.5 Hz, 1H, NH), 6.19 (*t*, *J* = 5.8 Hz, 1H, NH), 5.69 (*s*, 1H, *trans*-H^3), 5.25 (*d*, *J* = 7.8 Hz, 1H, *cis*-H^3), 5.11 (*d*, *J* = 7.5 Hz, 1H, *trans*-H^{6a}), 4.82 (*d*, *J* = 9.0 Hz, 1H, *cis*-H^{6a}), 4.00 (*t*, *J* = 9.0 Hz, 1H, *cis*-H^{3a}), 3.99 (*d*, *J* = 7.5 Hz, 1H, *trans*-H^{3a}), 3.95 (*t*, *J* = 6.5 Hz, 2H, -CH_2OAr), 3.90 (*t*, *J* = 6.5 Hz, 2H, -CH_2OAr), 3.67-3.61 (*m*, 6H, 2x –OH, 2x -CH_2OH), 3.41 (*quin*, *J* = 5.5 Hz, 4H, 2x -NCH_2), 1.80-1.26 (*m*, 76H, CH_2), 0.88 (*t*, *J* = 7.0 Hz, 6H, 2x CH_3); ^{13}C NMR (101 MHz, $CDCl_3$) δ 173.95, 173.47, 172.42, 171.25, 166.40, 159.56, 159.06, 148.75, 147.30, 138.64, 135.29, 135.03, 133.43, 130.24, 129.35, 128.77, 128.66, 127.78, 127.72, 127.55, 126.21, 125.93, 125.78, 124.91, 122.98, 119.14, 114.99, 114.89, 114.59, 71.33, 69.73, 68.16, 68.04, 63.05, 57.36, 54.69, 40.24, 32.82, 31.92, 29.66, 29.63, 29.60, 29.58, 29.54, 29.50, 29.46, 29.43, 29.40, 29.35, 29.24, 27.01, 26.03, 25.76, 25.74, 22.69, 14.11.

3-(Furan-2-yl)-2,5-diphenyltetrahydro-4H-pyrrolo[3,4-d]isoxazole-4,6(5H)-dione (***3u***)[1]

Beige solid, mp 144–145 °C; ^{1}H NMR (400 MHz, $CDCl_3$), mixture of *cis/trans*-diastereoisomers (*33/67*): δ 7.54-7.34 (*m*, 7H, Ar, 1H, furyl), 7.28-7.22 (*m*, 5H, Ar), 7.11 (*d*, *J* = 7.6 Hz, 3H, Ar), 7.05 (*d*, *J* = 8.6 Hz, 1H, Ar), 7.00 (*t*, *J* = 7.4 Hz, 1H, Ar), 6.77-6.75 (*m*, 2H, Ar), 6.37-6.32 (*m*, 3H, furyl), 5.68 (*s*, 1H, *trans*-H^3), 5.26 (*d*, *J* = 7.8 Hz, 1H, *cis*-H^3), 5.20 (*d*, *J* = 7.5 Hz, 1H, *trans*-H^{6a}), 4.86 (*d*, *J* = 9.2 Hz, 1H, *cis*-H^{6a}), 4.18 (*d*, *J* = 7.5 Hz, 1H, *trans*-H^{3a}), 4.00 (*t*, *J* = 9.2 Hz, 1H, *cis*-H^{3a}); ^{13}C NMR (101 MHz,

$CDCl_3$) δ 173.79, 173.20, 172.70, 171.46, 150.33, 147.64, 147.05, 146.85, 143.42, 142.85, 131.46, 131.04, 129.24, 129.22, 129.06, 129.02, 128.87, 128.84, 126.23, 126.21, 125.33, 123.39, 118.82, 115.32, 110.92, 110.82, 110.71, 108.50, 66.73, 64.36, 53.71, 52.77.

References

1. Iwakura, Y.; Uno, K.; Hongu, T. *Bull. Chem. Soc. Jpn.* **1969**, *42*, 2882–2885. doi: 10.1246/bcsj.42.2882
2. Yıldırım, A.; Kaya, Y. *J. Phys. Org. Chem.* **2017**, *30*, e3629. doi: 10.1002/poc.3629
3. Kaur, M.; Singh, B.; Singh, B. *WJPPS* **2014**, *3*, 1299–1317
4. Singal, K. K.; Giare, R. *Chem. Environ. Res.* **1998**, *7*, 93–98

3a

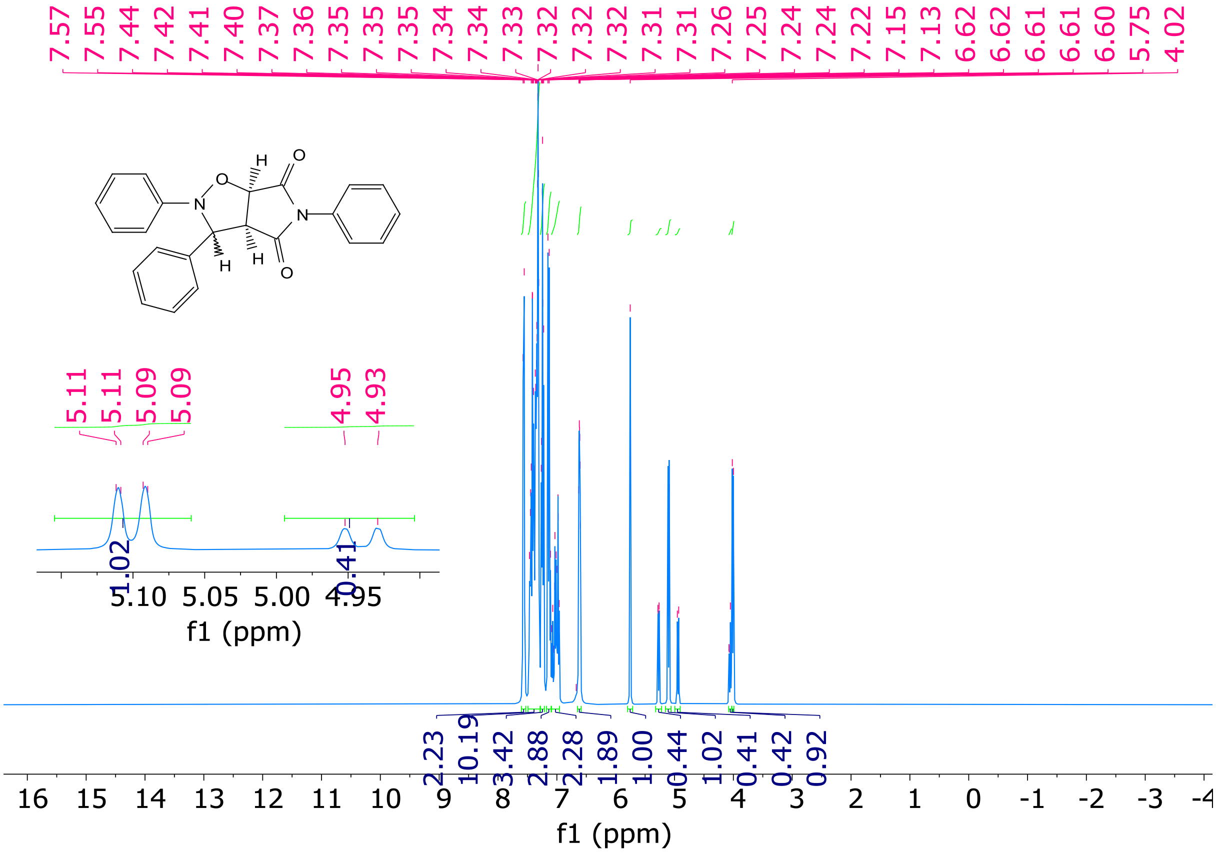

7.57
7.55
7.44
7.42
7.41
7.40
7.37
7.36
7.35
7.35
7.35
7.34
7.34
7.33
7.32
7.32
7.32
7.31
7.31
7.26
7.25
7.24
7.24
7.22
7.15
7.13
6.62
6.62
6.61
6.61
6.60
5.75
4.02
5.11
5.11
5.09
5.09
4.95
4.93
1.02
0.41
5.10 5.05 5.00 4.95
f1 (ppm)
2.23
10.19
3.42
2.88
2.28
1.89
1.00
0.44
1.02
0.41
0.42
0.92
16 15 14 13 12 11 10 9 8 7 6 5 4 3 2 1 0 -1 -2 -3 -4
f1 (ppm)

3b

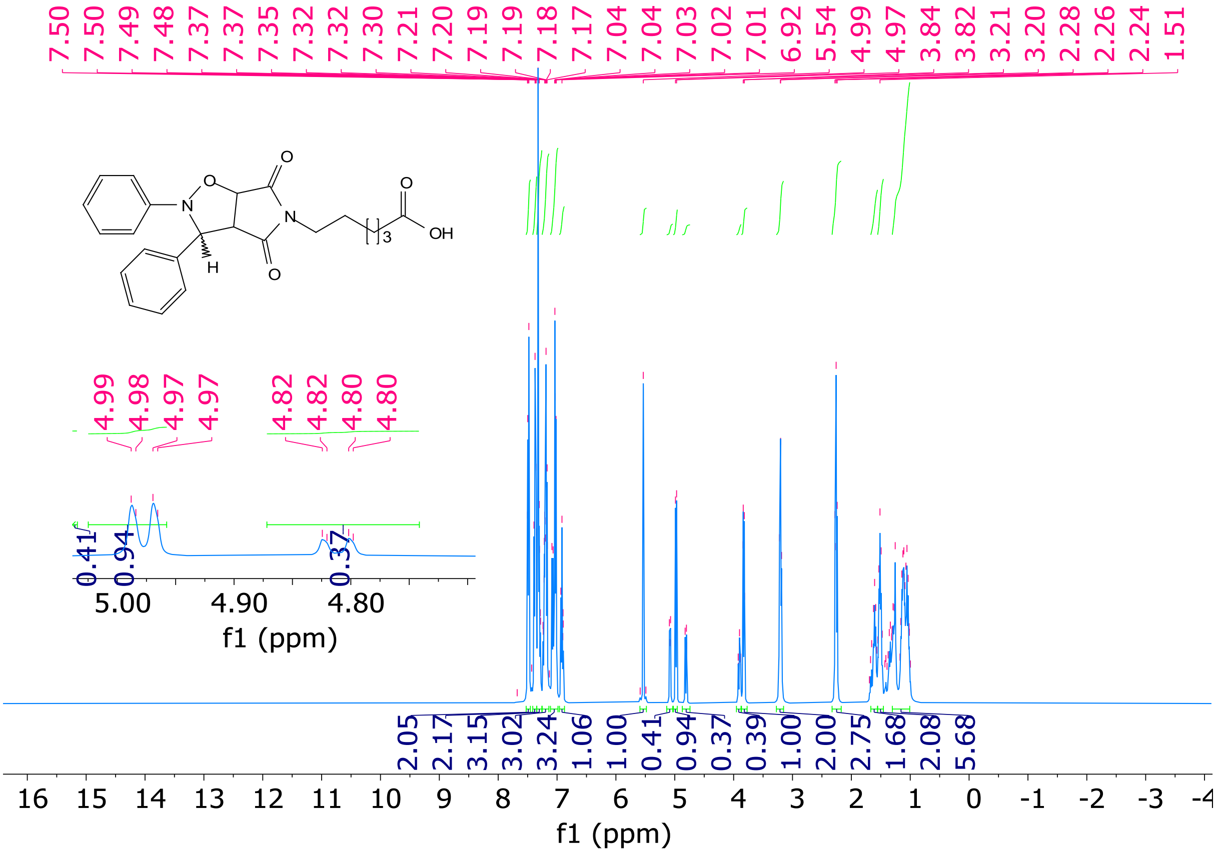
7.50
7.50
7.49
7.48
7.37
7.37
7.35
7.32
7.32
7.30
7.21
7.20
7.19
7.19
7.18
7.17
7.04
7.04
7.03
7.02
7.01
6.92
5.54
4.99
4.97
3.84
3.82
3.21
3.20
2.28
2.26
2.24
1.51
O
N
O
H
O
N
3
O
OH
4.99
4.98
4.97
4.97
4.82
4.82
4.80
4.80
0.41
0.94
0.37
5.00
4.90
4.80
f1 (ppm)
2.05
2.17
3.15
3.02
3.24
1.06
1.00
0.41
0.94
0.37
0.39
1.00
2.00
2.75
1.68
2.08
5.68
16
15
14
13
12
11
10
9
8
7
6
5
4
3
2
1
0
-1
-2
-3
-4
f1 (ppm)

3c

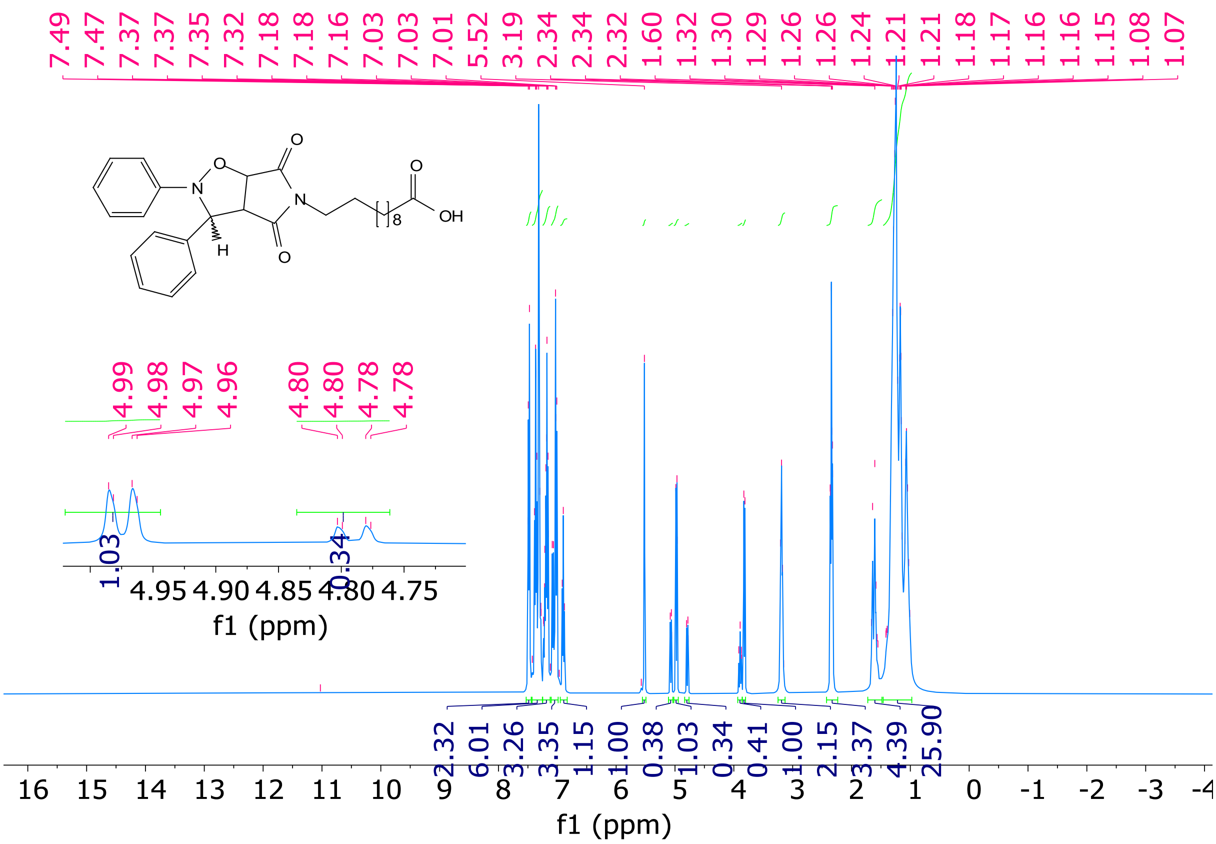
7.49
7.47
7.37
7.37
7.35
7.32
7.18
7.18
7.16
7.03
7.03
7.01
5.52
3.19
2.34
2.34
2.32
1.60
1.32
1.30
1.29
1.26
1.26
1.24
1.21
1.21
1.18
1.17
1.16
1.16
1.15
1.08
1.07
O
N
O
O
N
8
O
OH
H
4.99
4.98
4.97
4.96
4.80
4.80
4.78
4.78
1.03
0.34
4.95 4.90 4.85 4.80 4.75
f1 (ppm)
2.32
6.01
3.26
3.35
1.15
1.00
0.38
1.03
0.34
0.41
1.00
2.15
3.37
4.39
25.90
16 15 14 13 12 11 10 9 8 7 6 5 4 3 2 1 0 -1 -2 -3 -4
f1 (ppm)

3d

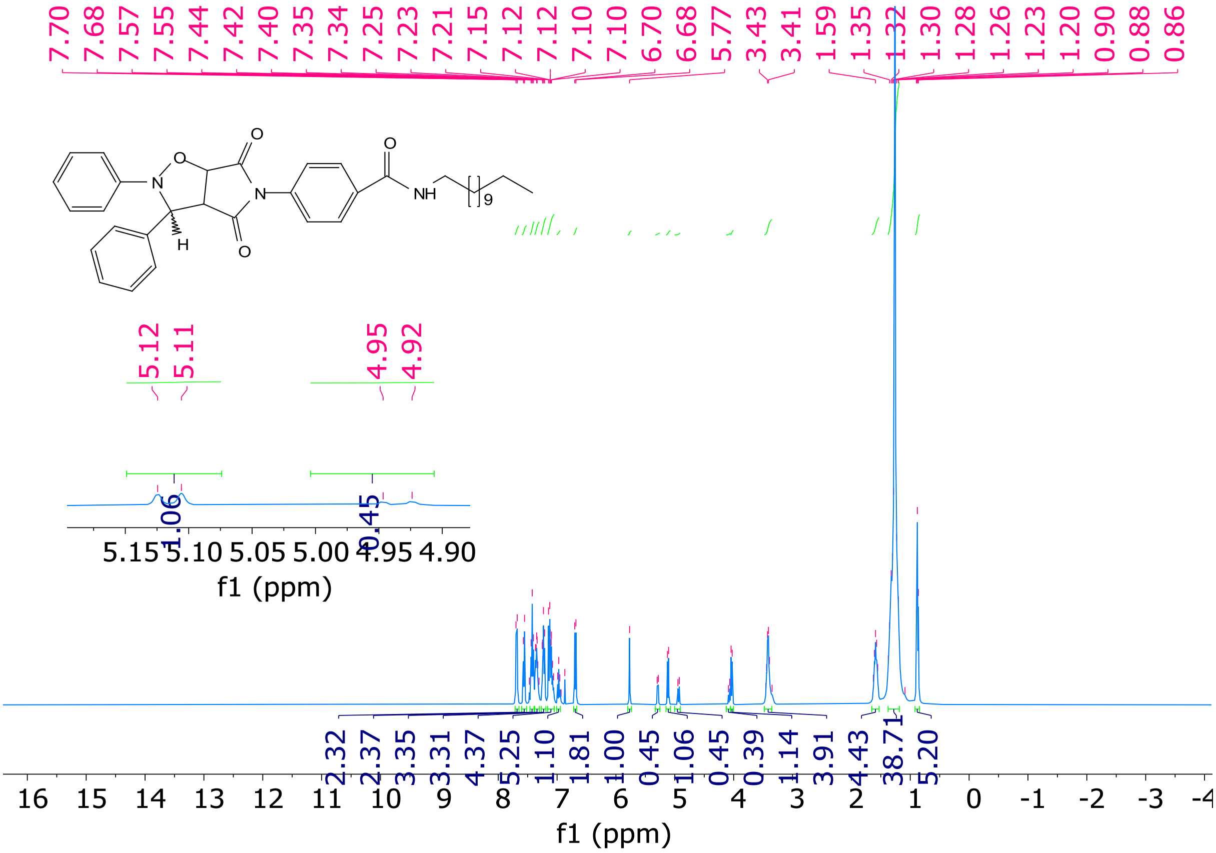

7.70 7.68 7.57 7.55 7.44 7.42 7.40 7.35 7.34 7.25 7.23 7.21 7.15 7.12 7.12 7.10 7.10 6.70 6.68 5.77 3.43 3.41 1.59 1.35 1.32 1.30 1.28 1.26 1.23 1.20 0.90 0.88 0.86
O
N
O
H
O
N
O
NH
9
5.12 5.11 4.95 4.92
1.06 0.45
5.15 5.10 5.05 5.00 4.95 4.90
f1 (ppm)
2.32 2.37 3.35 3.31 4.37 5.25 1.10 1.81 1.00 0.45 1.06 0.45 0.39 1.14 3.91 4.43 38.71 5.20
16 15 14 13 12 11 10 9 8 7 6 5 4 3 2 1 0 -1 -2 -3 -4
f1 (ppm)

3e

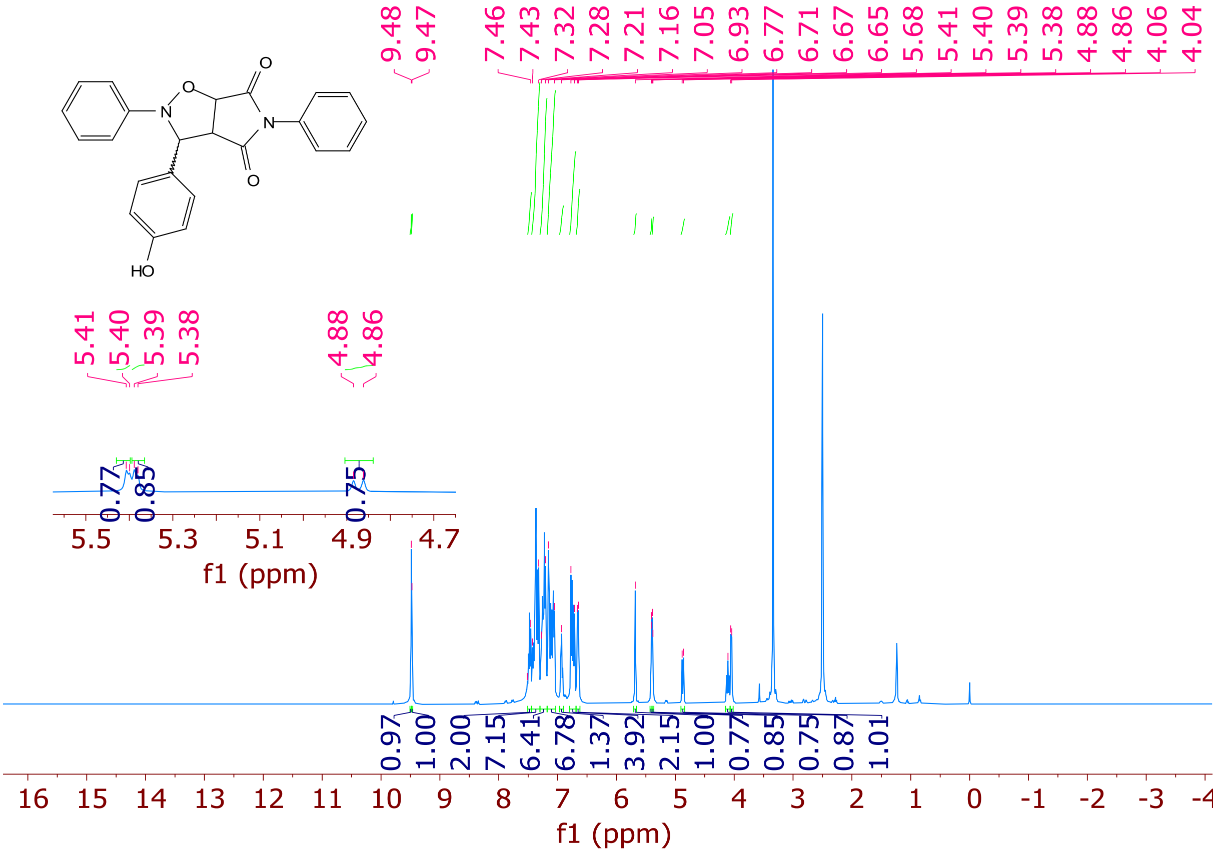

9.48
9.47
7.46
7.43
7.32
7.28
7.21
7.16
7.05
6.93
6.77
6.71
6.67
6.65
5.68
5.41
5.40
5.39
5.38
4.88
4.86
4.06
4.04
O
N
O
N
O
HO
5.41
5.40
5.39
5.38
4.88
4.86
0.77
0.85
0.75
5.5
5.3
5.1
4.9
4.7
f1 (ppm)
0.97
1.00
2.00
7.15
6.41
6.78
1.37
3.92
2.15
1.00
0.77
0.85
0.75
0.87
1.01
16
15
14
13
12
11
10
9
8
7
6
5
4
3
2
1
0
-1
-2
-3
-4
f1 (ppm)

3f

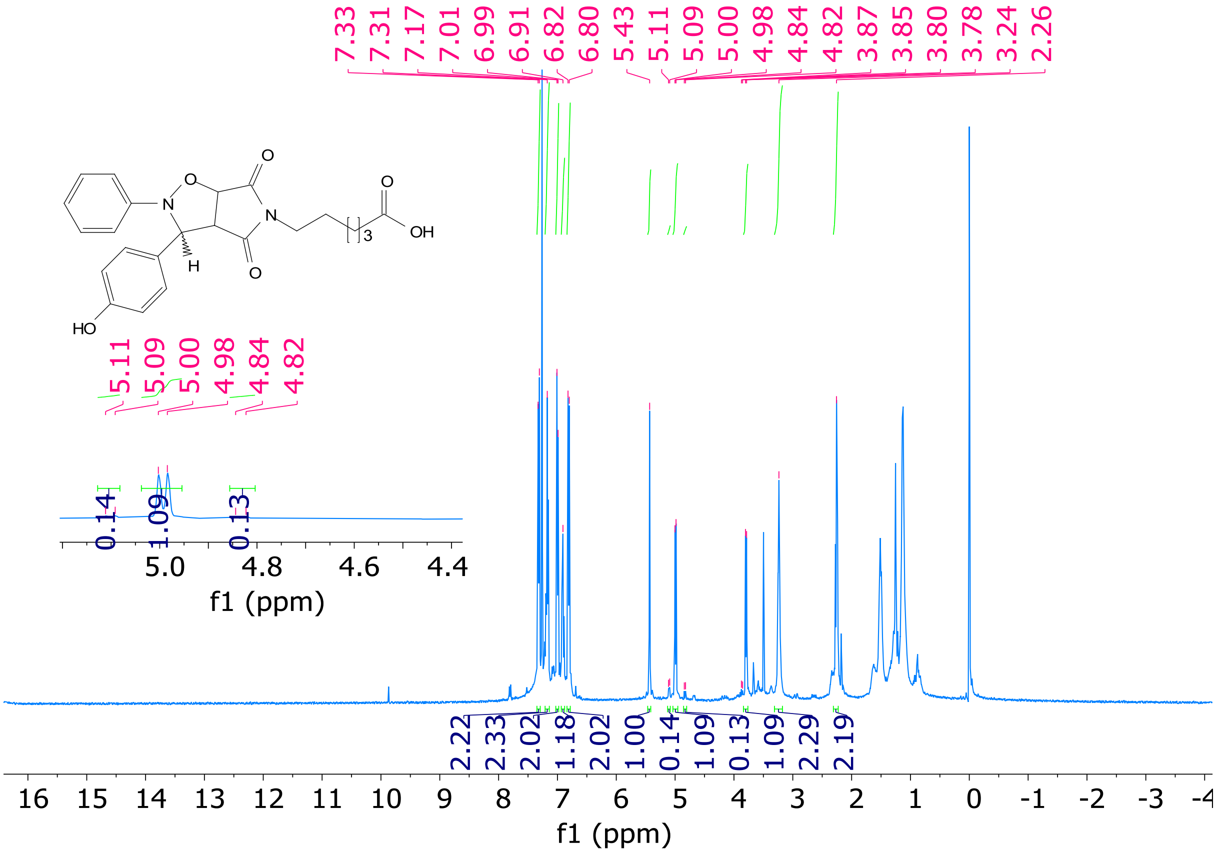

7.33
7.31
7.17
7.01
6.99
6.91
6.82
6.80
5.43
5.11
5.09
5.00
4.98
4.84
4.82
3.87
3.85
3.80
3.78
3.24
2.26
O
N
O
N
O
O
OH
3
H
HO
5.11
5.09
5.00
4.98
4.84
4.82
0.14
1.09
0.13
5.0
4.8
4.6
4.4
f1 (ppm)
2.22
2.33
2.02
1.18
2.02
1.00
0.14
1.09
0.13
1.09
2.29
2.19
16
15
14
13
12
11
10
9
8
7
6
5
4
3
2
1
0
-1
-2
-3
f1 (ppm)

3g

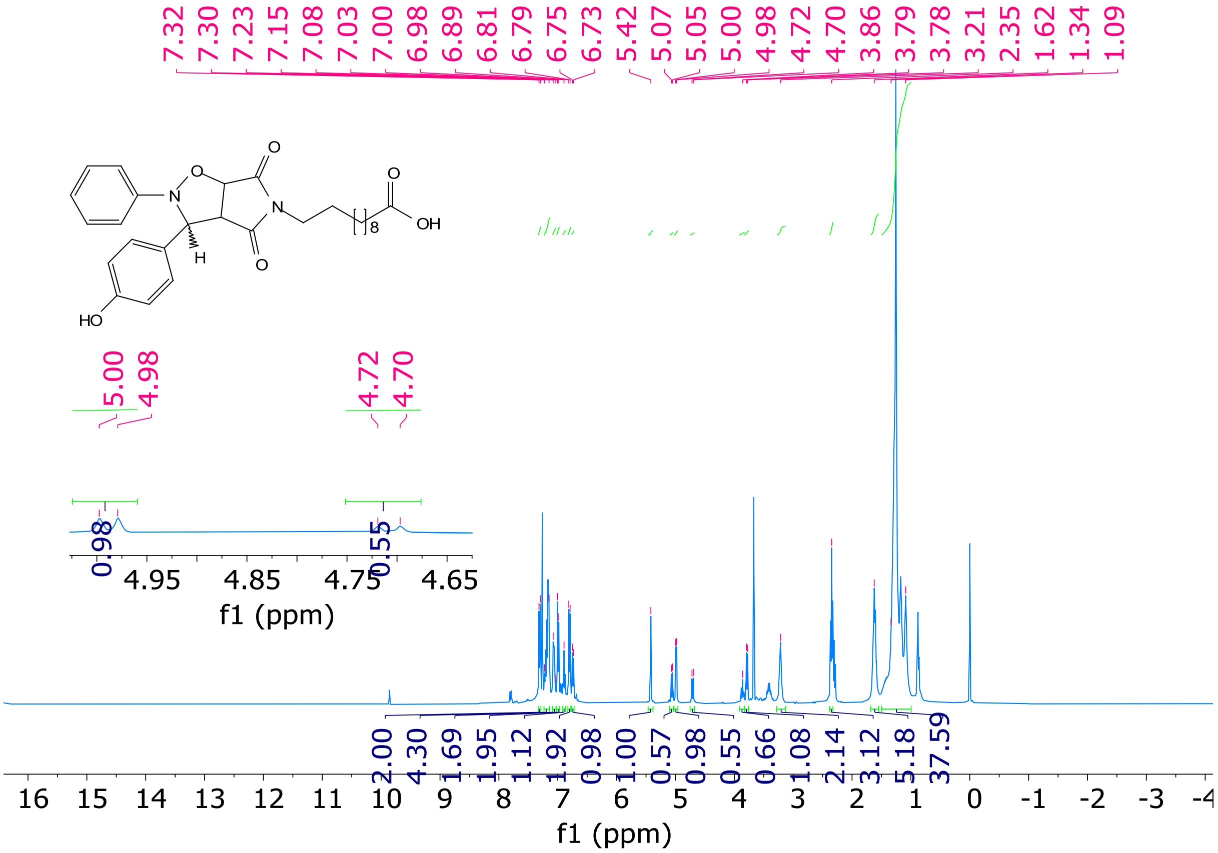

7.32
7.30
7.23
7.15
7.08
7.03
7.00
6.98
6.89
6.81
6.79
6.75
6.73
5.42
5.07
5.05
5.00
4.98
4.72
4.70
3.86
3.79
3.78
3.21
2.35
1.62
1.34
1.09
O
O
N
O
OH
8
H
O
N
O
HO
5.00
4.98
4.72
4.70
0.98
0.55
4.95
4.85
4.75
4.65
f1 (ppm)
2.00
4.30
1.69
1.95
1.12
1.92
0.98
1.00
0.57
0.98
0.55
0.66
1.08
2.14
3.12
5.18
37.59
16
15
14
13
12
11
10
9
8
7
6
5
4
3
2
1
0
-1
-2
-3
-4
f1 (ppm)

3h

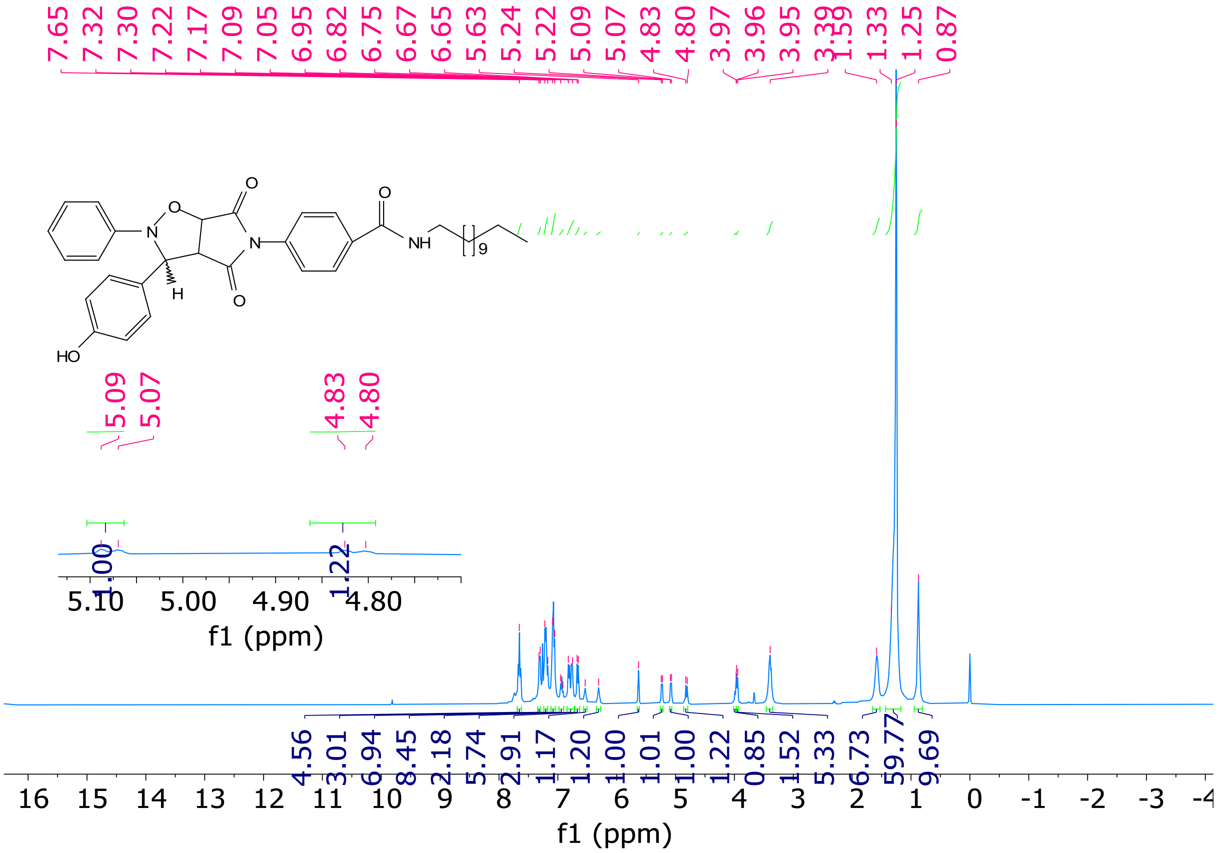

3i

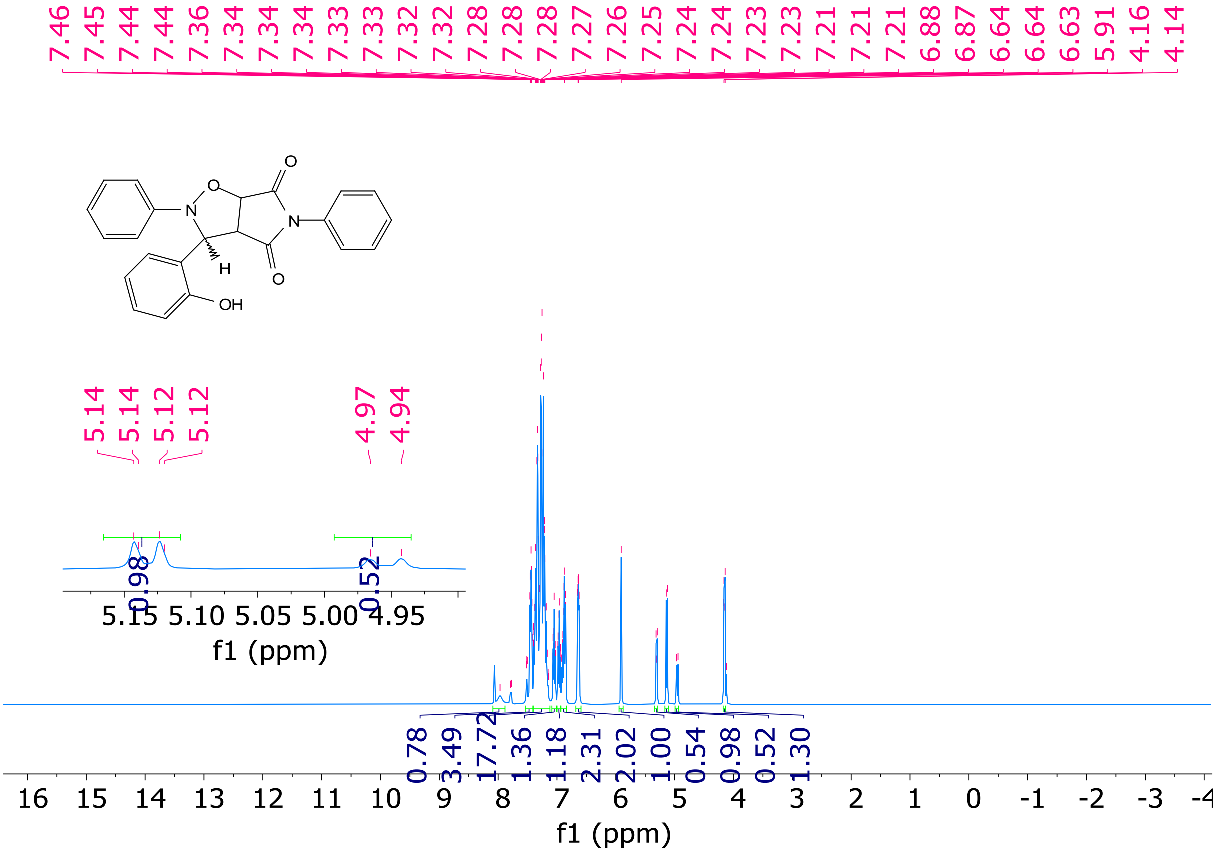

7.46
7.45
7.44
7.44
7.36
7.34
7.34
7.34
7.33
7.33
7.32
7.32
7.28
7.28
7.28
7.27
7.26
7.25
7.24
7.24
7.23
7.23
7.21
7.21
7.21
6.88
6.87
6.64
6.64
6.63
5.91
4.16
4.14
O
N
O
N
O
H
OH
5.14
5.14
5.12
5.12
4.97
4.94
0.98
0.52
5.15 5.10 5.05 5.00 4.95
f1 (ppm)
0.78
3.49
17.72
1.36
1.18
2.31
2.02
1.00
0.54
0.98
0.52
1.30
16 15 14 13 12 11 10 9 8 7 6 5 4 3 2 1 0 -1 -2 -3 -4
f1 (ppm)

3j

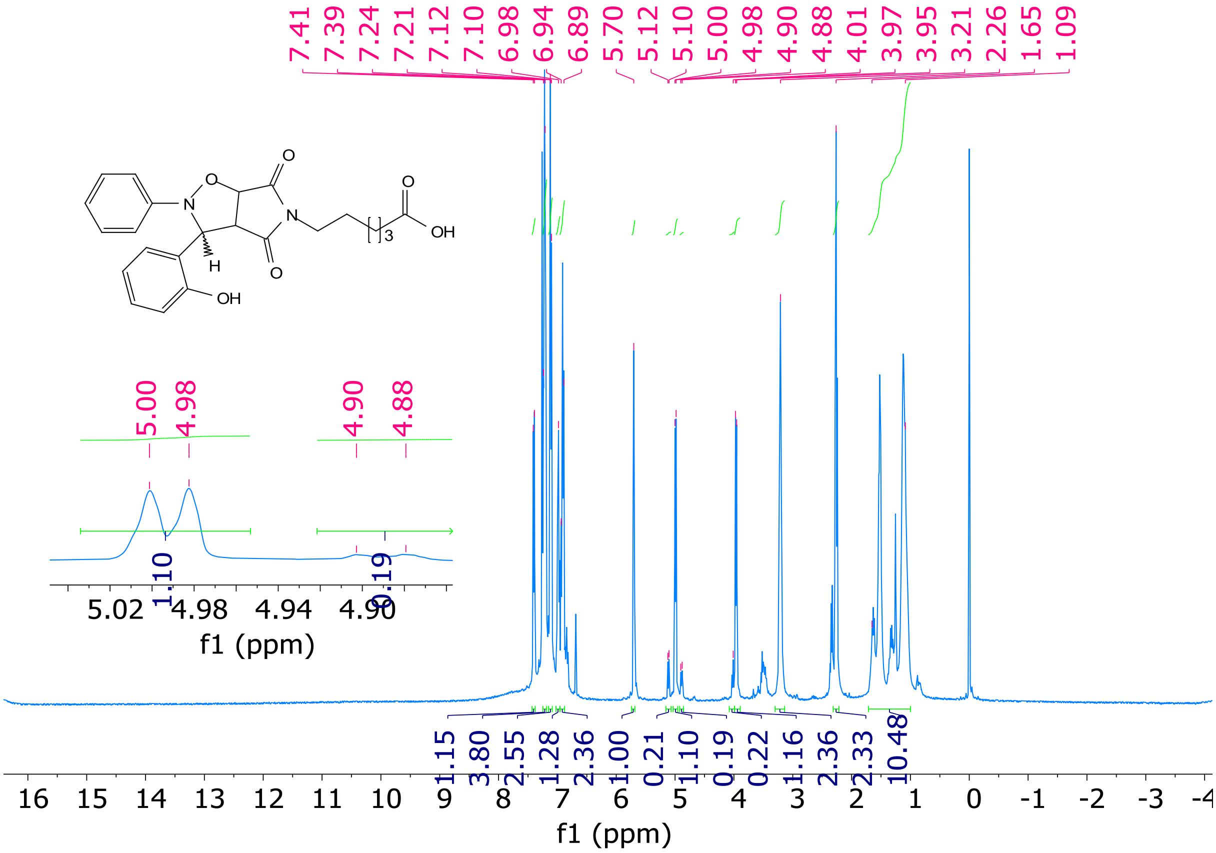
7.41
7.39
7.24
7.21
7.12
7.10
6.98
6.94
6.89
5.70
5.12
5.10
5.00
4.98
4.90
4.88
4.01
3.97
3.95
3.21
2.26
1.65
1.09
O
N
O
O
N
3
O
OH
H
OH
5.00
4.98
4.90
4.88
1.10
0.19
5.02
4.98
4.94
4.90
f1 (ppm)
1.15
3.80
2.55
1.28
2.36
1.00
0.21
1.10
0.19
0.22
1.16
2.36
2.33
10.48
16
15
14
13
12
11
10
9
8
7
6
5
4
3
2
1
0
-1
-2
-3
-4
f1 (ppm)

3k

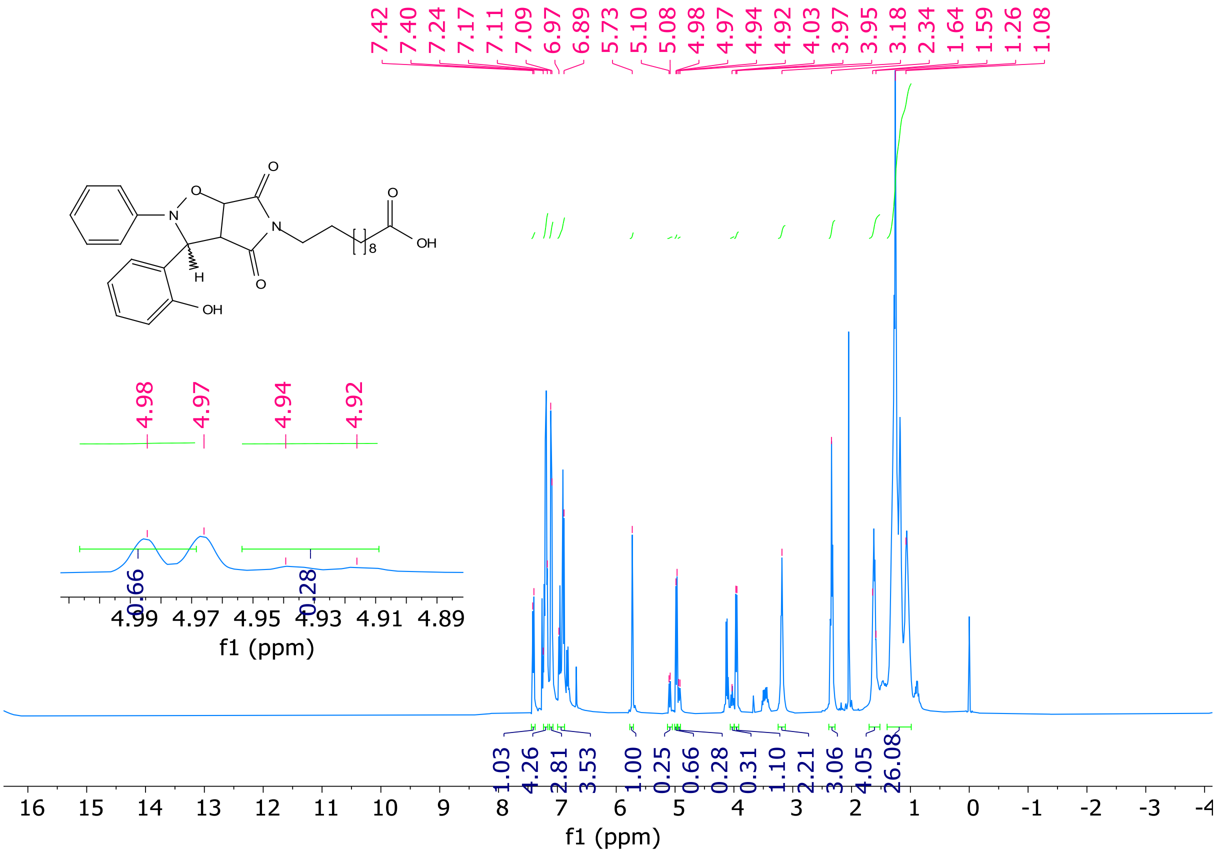
7.42 7.40 7.24 7.17 7.11 7.09 6.97 6.89 5.73 5.10 5.08 4.98 4.97 4.94 4.92 4.03 3.97 3.95 3.18 2.34 1.64 1.59 1.26 1.08
4.98 4.97 4.94 4.92
0.66 0.28
4.99 4.97 4.95 4.93 4.91 4.89
f1 (ppm)
1.03 4.26 2.81 3.53 1.00 0.25 0.66 0.28 0.31 1.10 2.21 3.06 4.05 26.08
16 15 14 13 12 11 10 9 8 7 6 5 4 3 2 1 0 -1 -2 -3 -4
f1 (ppm)

3l

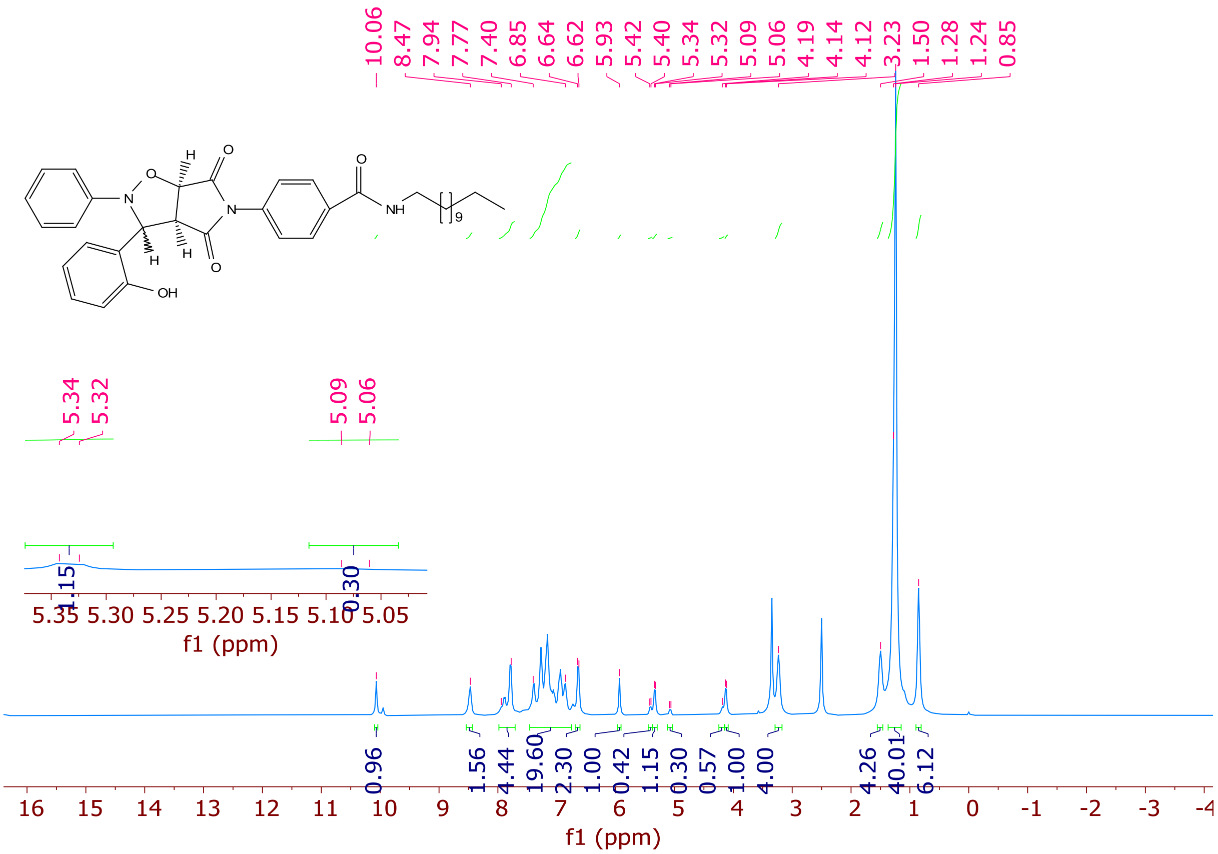
10.06
8.47
7.94
7.77
7.40
6.85
6.64
6.62
5.93
5.42
5.40
5.34
5.32
5.09
5.06
4.19
4.14
4.12
3.23
1.50
1.28
1.24
0.85
O
H
N
NH
OH
9
5.34
5.32
5.09
5.06
1.15
0.30
5.35 5.30 5.25 5.20 5.15 5.10 5.05
f1 (ppm)
0.96
1.56
4.44
19.60
2.30
1.00
0.42
1.15
0.30
0.57
1.00
4.00
4.26
40.01
6.12
16 15 14 13 12 11 10 9 8 7 6 5 4 3 2 1 0 -1 -2 -3 -4
f1 (ppm)

3m

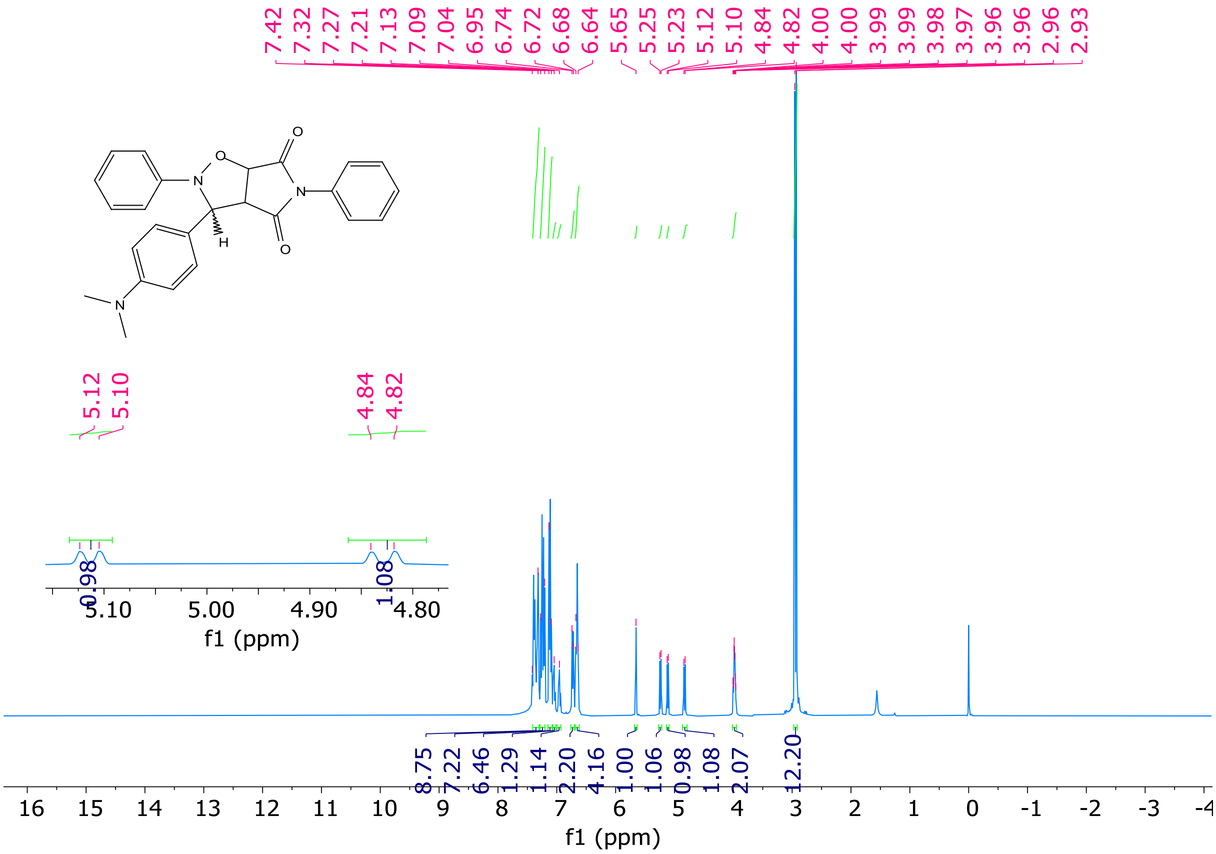

7.42
7.32
7.27
7.21
7.13
7.09
7.04
6.95
6.74
6.72
6.68
6.64
5.65
5.25
5.23
5.12
5.10
4.84
4.82
4.00
4.00
3.99
3.99
3.98
3.97
3.96
3.96
2.96
2.93
5.12
5.10
4.84
4.82
0.98
1.08
5.10
5.00
4.90
4.80
f1 (ppm)
8.75
7.22
6.46
1.29
1.14
2.20
4.16
1.00
1.06
0.98
1.08
2.07
12.20
16 15 14 13 12 11 10 9 8 7 6 5 4 3 2 1 0 -1 -2 -3 -4
f1 (ppm)

3n

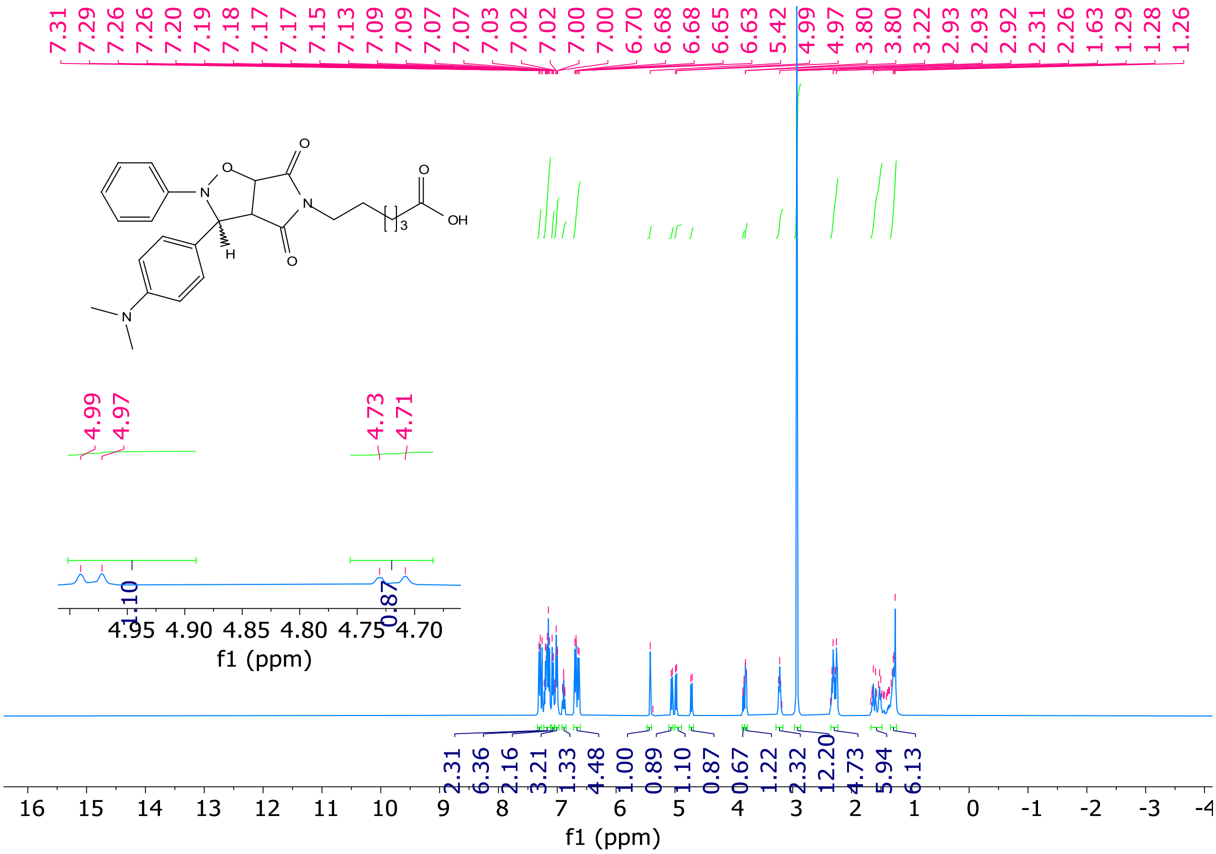

7.31
7.29
7.26
7.26
7.20
7.19
7.18
7.17
7.17
7.15
7.13
7.09
7.09
7.07
7.07
7.03
7.02
7.02
7.00
7.00
6.70
6.68
6.68
6.65
6.63
5.42
4.99
4.97
3.80
3.80
3.22
2.93
2.93
2.92
2.31
2.26
1.63
1.29
1.28
1.26
O
N
O
O
N
[ ]3
O
OH
H
N
4.99
4.97
4.73
4.71
1.10
0.87
4.95 4.90 4.85 4.80 4.75 4.70
f1 (ppm)
2.31
6.36
2.16
3.21
1.33
4.48
1.00
0.89
1.10
0.87
0.67
1.22
2.32
12.20
4.73
5.94
6.13
16 15 14 13 12 11 10 9 8 7 6 5 4 3 2 1 0 -1 -2 -3 -4
f1 (ppm)

3o

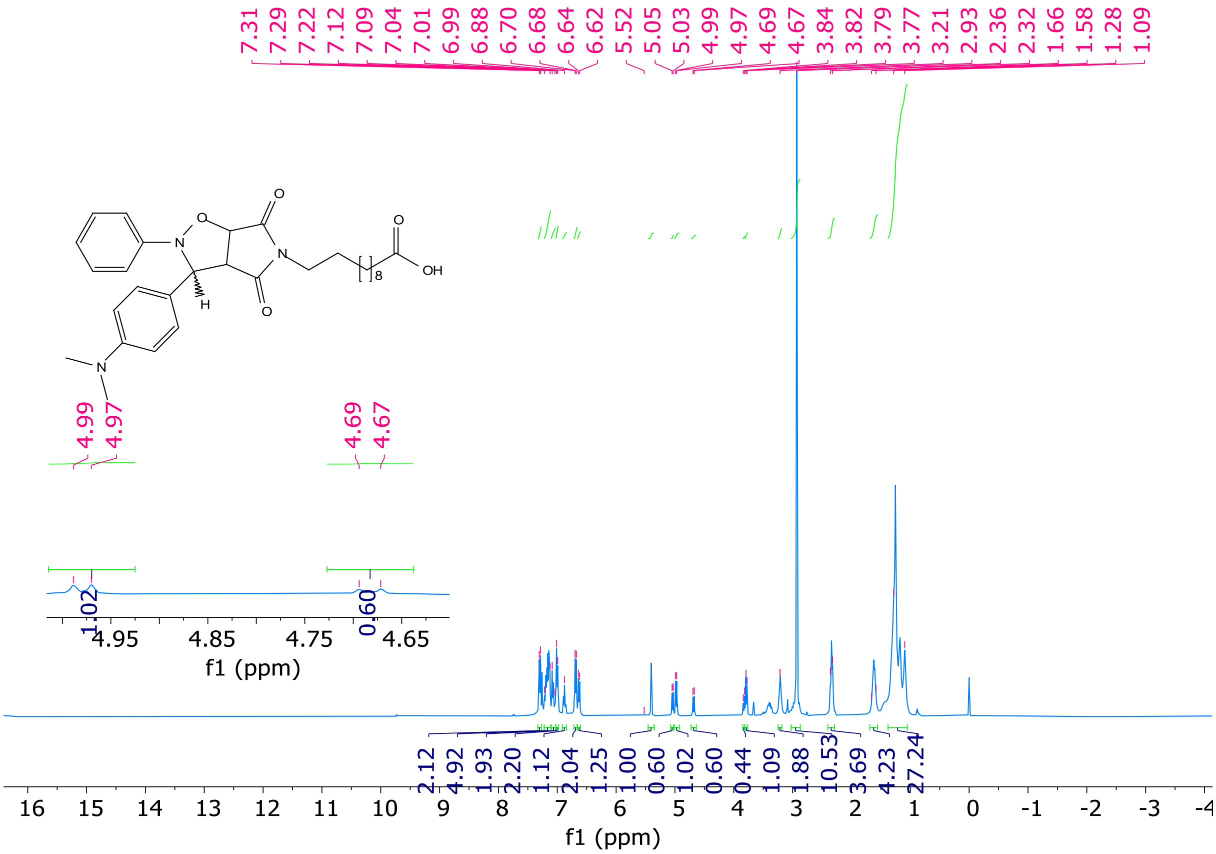

7.31
7.29
7.22
7.12
7.09
7.04
7.01
6.99
6.88
6.70
6.68
6.64
6.62
5.52
5.05
5.03
4.99
4.97
4.69
4.67
3.84
3.82
3.79
3.77
3.21
2.93
2.36
2.32
1.66
1.58
1.28
1.09
4.99
4.97
4.69
4.67
1.02
0.60
4.95
4.85
4.75
4.65
f1 (ppm)
2.12
4.92
1.93
2.20
1.12
2.04
1.25
1.00
0.60
1.02
0.60
0.44
1.09
1.88
10.53
3.69
4.23
27.24
16
15
14
13
12
11
10
9
8
7
6
5
4
3
2
1
0
-1
-2
-3
-4
f1 (ppm)

3p

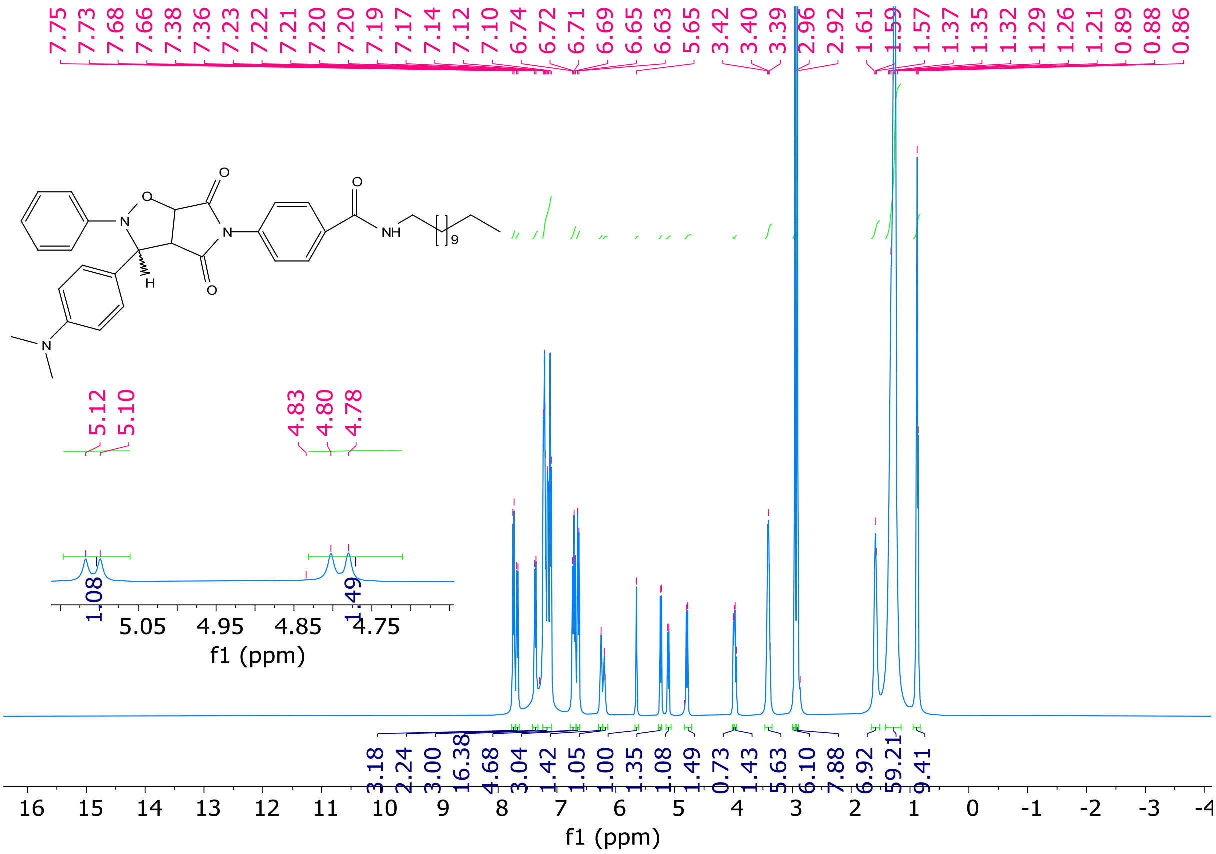

7.75
7.73
7.68
7.66
7.38
7.36
7.23
7.22
7.21
7.20
7.20
7.19
7.17
7.14
7.12
7.10
6.74
6.72
6.71
6.69
6.65
6.63
5.65
3.42
3.40
3.39
2.96
2.92
1.61
1.59
1.57
1.37
1.35
1.32
1.29
1.26
1.21
0.89
0.88
0.86
O
N
NH
H
9
5.12
5.10
4.83
4.80
4.78
1.08
1.49
5.05
4.95
4.85
4.75
f1 (ppm)
3.18
2.24
3.00
16.38
4.68
3.04
1.42
1.05
1.00
1.35
1.08
1.49
0.73
1.43
5.63
6.10
7.88
6.92
59.21
9.41
16
15
14
13
12
11
10
9
8
7
6
5
4
3
2
1
0
-1
-2
-3
-4
f1 (ppm)

3q

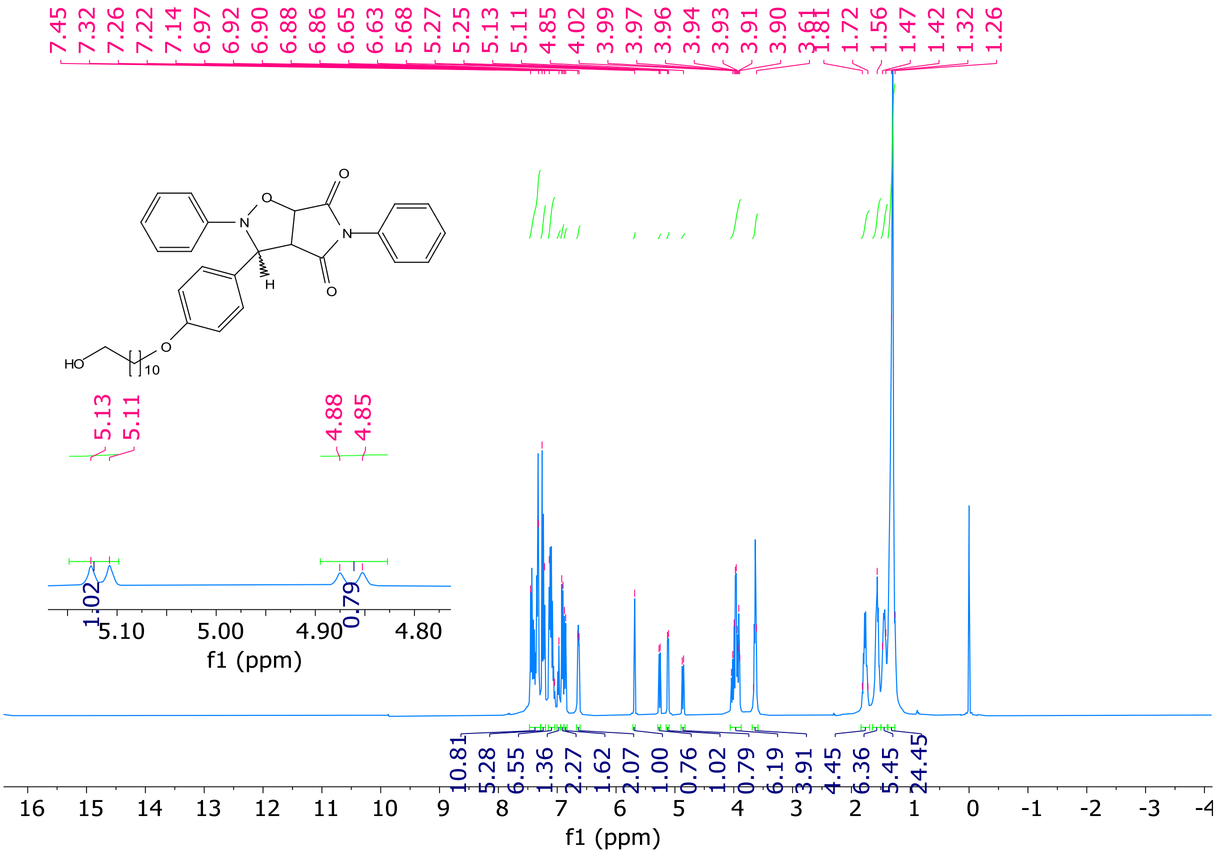

3r

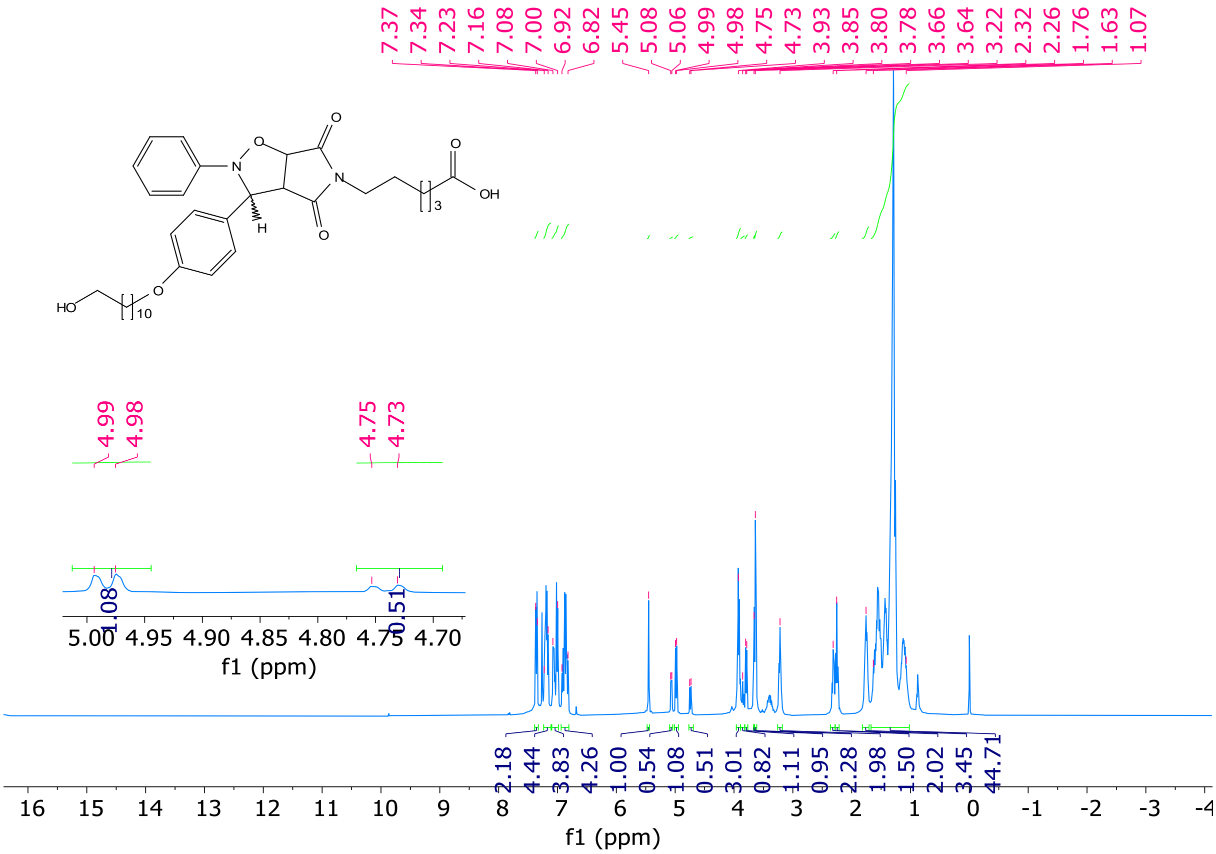

7.37 7.34 7.23 7.16 7.08 7.00 6.92 6.82 5.45 5.08 5.06 4.99 4.98 4.75 4.73 3.93 3.85 3.80 3.78 3.66 3.64 3.22 2.32 2.26 1.76 1.63 1.07
O
N
O
N
O
O
OH
H
3
HO
10
O
4.99 4.98
4.75 4.73
1.08
0.51
5.00 4.95 4.90 4.85 4.80 4.75 4.70
f1 (ppm)
2.18 4.44 3.83 4.26 1.00 0.54 1.08 0.51 3.01 0.82 1.11 0.95 2.28 1.98 1.50 2.02 3.45 44.71
16 15 14 13 12 11 10 9 8 7 6 5 4 3 2 1 0 -1 -2 -3 -4
f1 (ppm)

3s

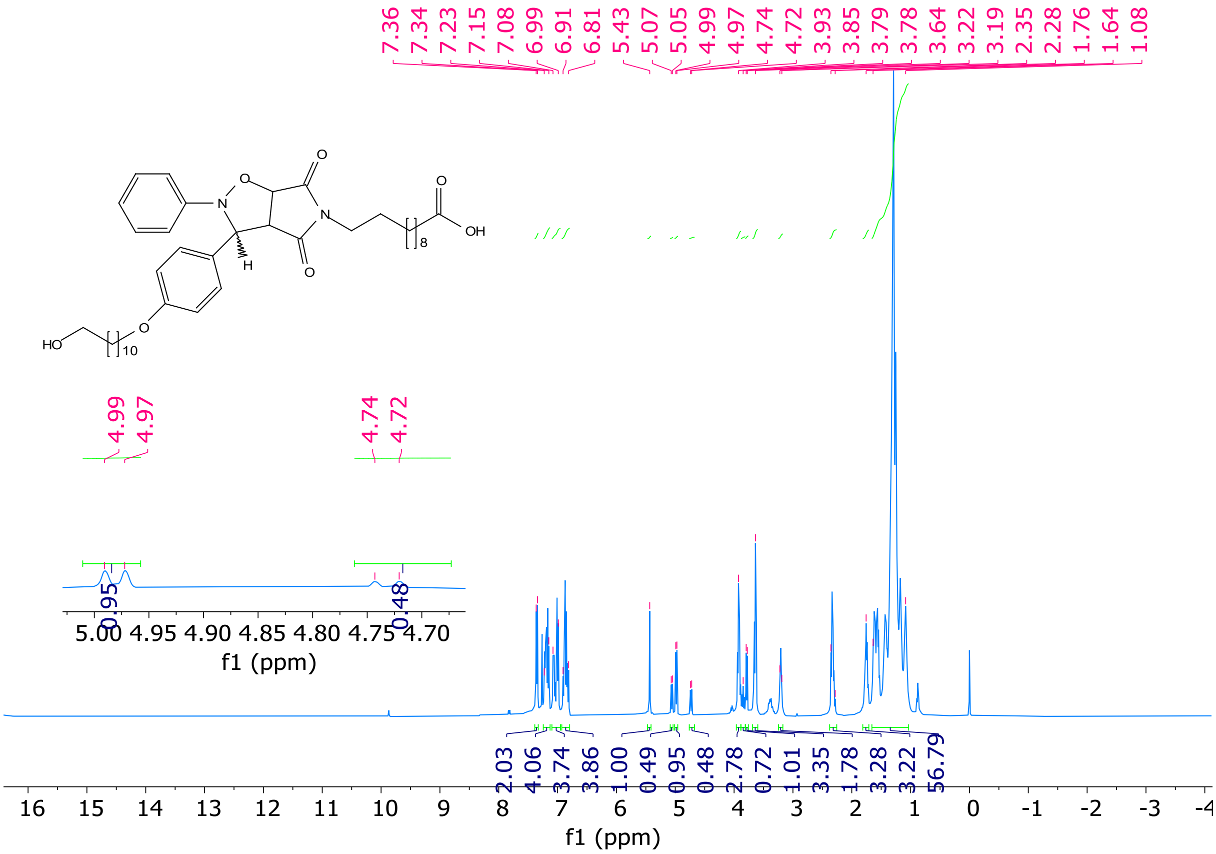

7.36
7.34
7.23
7.15
7.08
6.99
6.91
6.81
5.43
5.07
5.05
4.99
4.97
4.74
4.72
3.93
3.85
3.79
3.78
3.64
3.22
3.19
2.35
2.28
1.76
1.64
1.08
O
O
N
N
O
OH
O
H
8
O
HO
10
4.99
4.97
4.74
4.72
0.95
0.48
5.00 4.95 4.90 4.85 4.80 4.75 4.70
f1 (ppm)
2.03
4.06
3.74
3.86
1.00
0.49
0.95
0.48
2.78
0.72
1.01
3.35
1.78
3.28
3.22
56.79
16 15 14 13 12 11 10 9 8 7 6 5 4 3 2 1 0 -1 -2 -3 -4
f1 (ppm)

3t

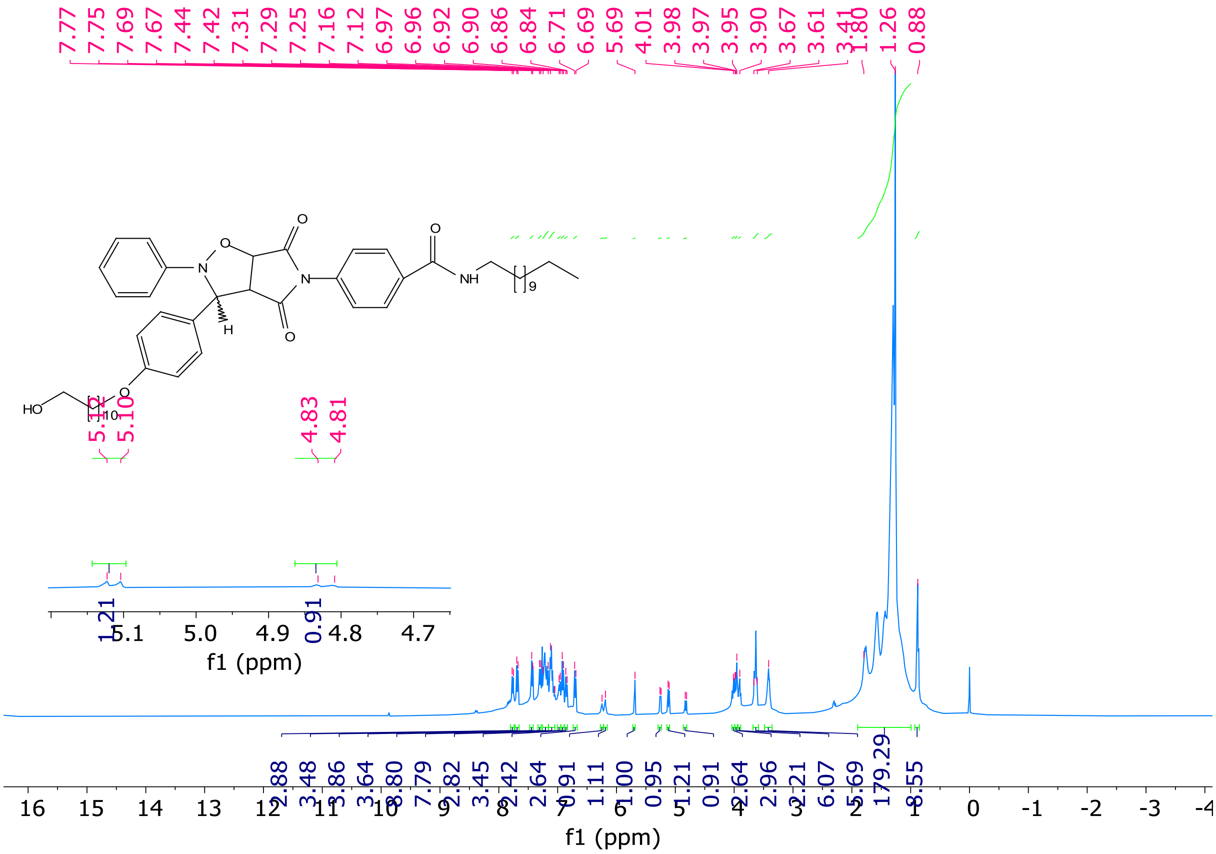

3u

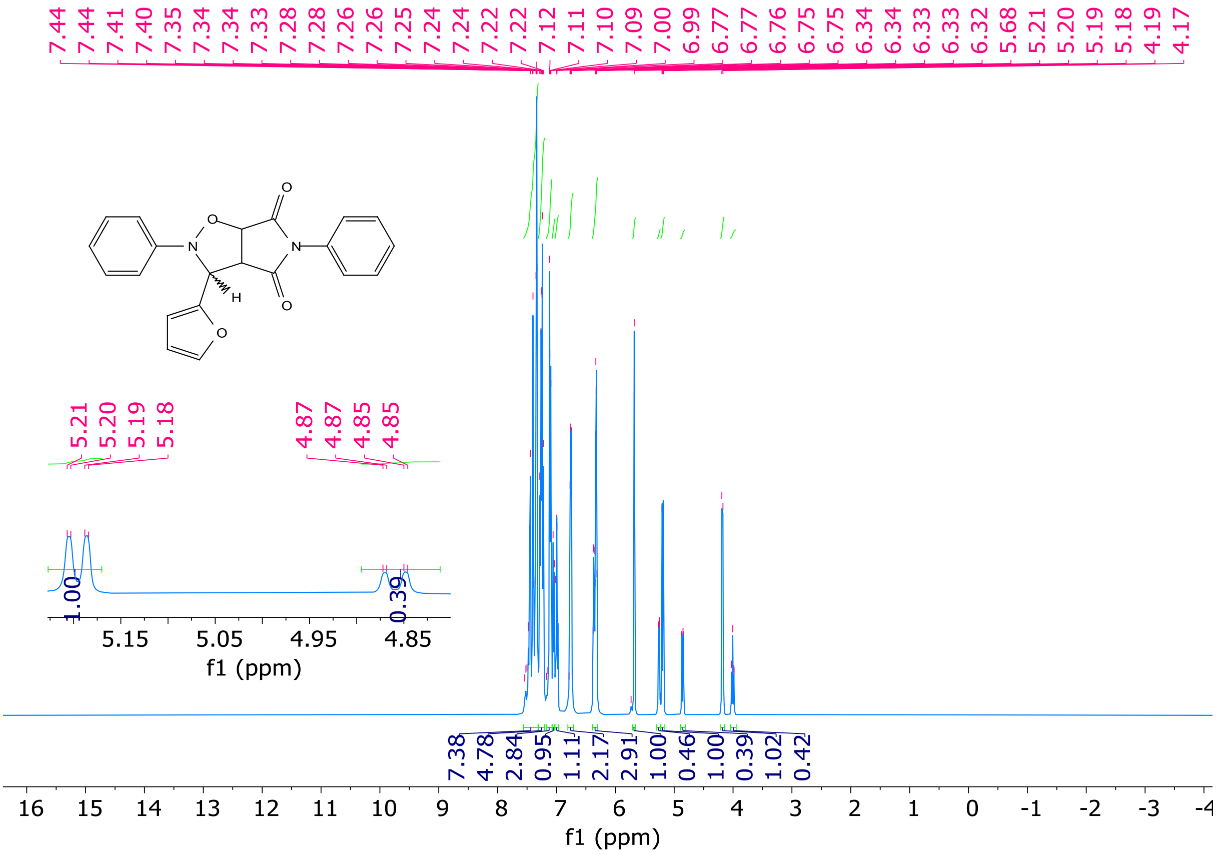

7.44
7.44
7.41
7.40
7.35
7.34
7.34
7.33
7.28
7.28
7.26
7.26
7.25
7.24
7.24
7.22
7.22
7.12
7.11
7.10
7.09
7.00
6.99
6.77
6.77
6.76
6.75
6.75
6.34
6.34
6.33
6.33
6.32
5.68
5.21
5.20
5.19
5.18
4.19
4.17
5.21
5.20
5.19
5.18
4.87
4.87
4.85
4.85
1.00
0.39
5.15
5.05
4.95
4.85
f1 (ppm)
7.38
4.78
2.84
0.95
1.11
2.17
2.91
1.00
0.46
1.00
0.39
1.02
0.42
16
15
14
13
12
11
10
9
8
7
6
5
4
3
2
1
0
-1
-2
-3
-4
f1 (ppm)

3a

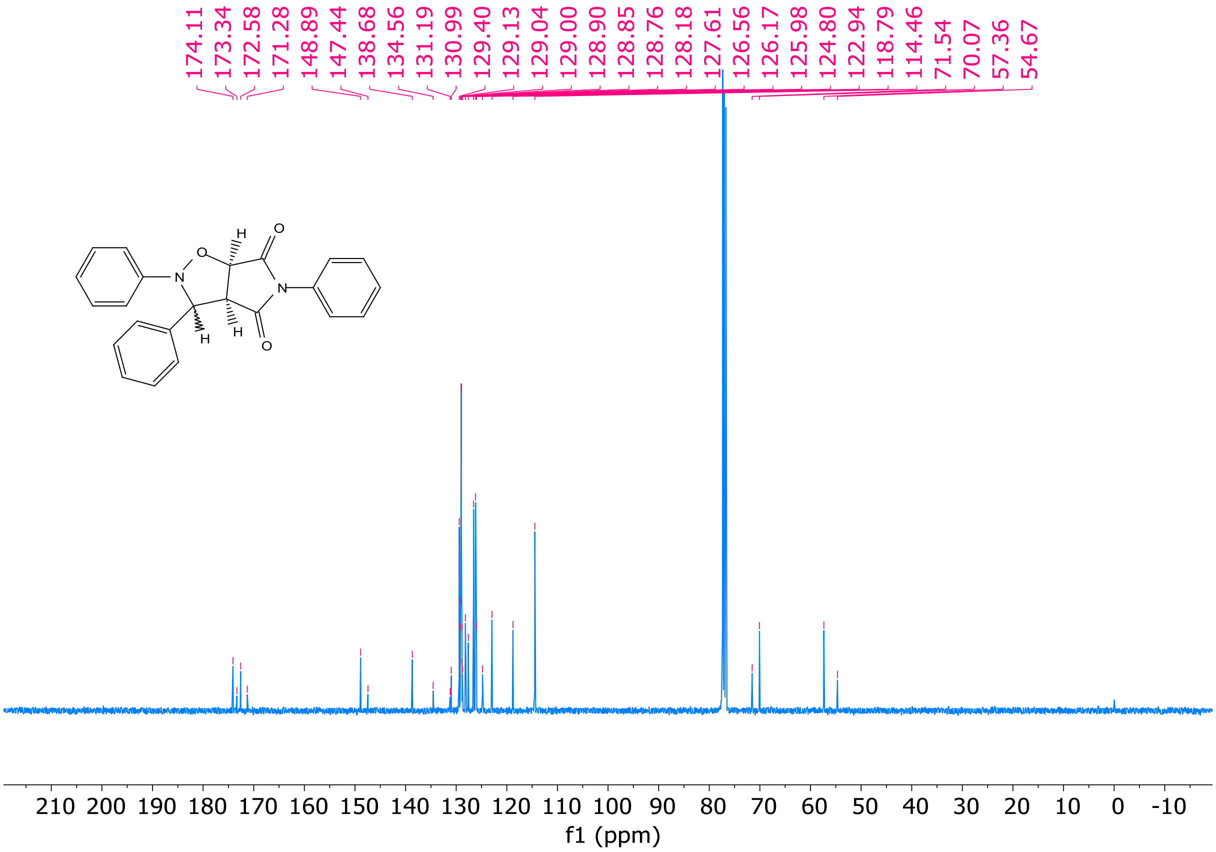
174.11
173.34
172.58
171.28
148.89
147.44
138.68
134.56
131.19
130.99
129.40
129.13
129.04
129.00
128.90
128.85
128.76
128.18
127.61
126.56
126.17
125.98
124.80
122.94
118.79
114.46
71.54
70.07
57.36
54.67
O
N
H
H
H
210 200 190 180 170 160 150 140 130 120 110 100 90 80 70 60 50 40 30 20 10 0 -10
f1 (ppm)

3b

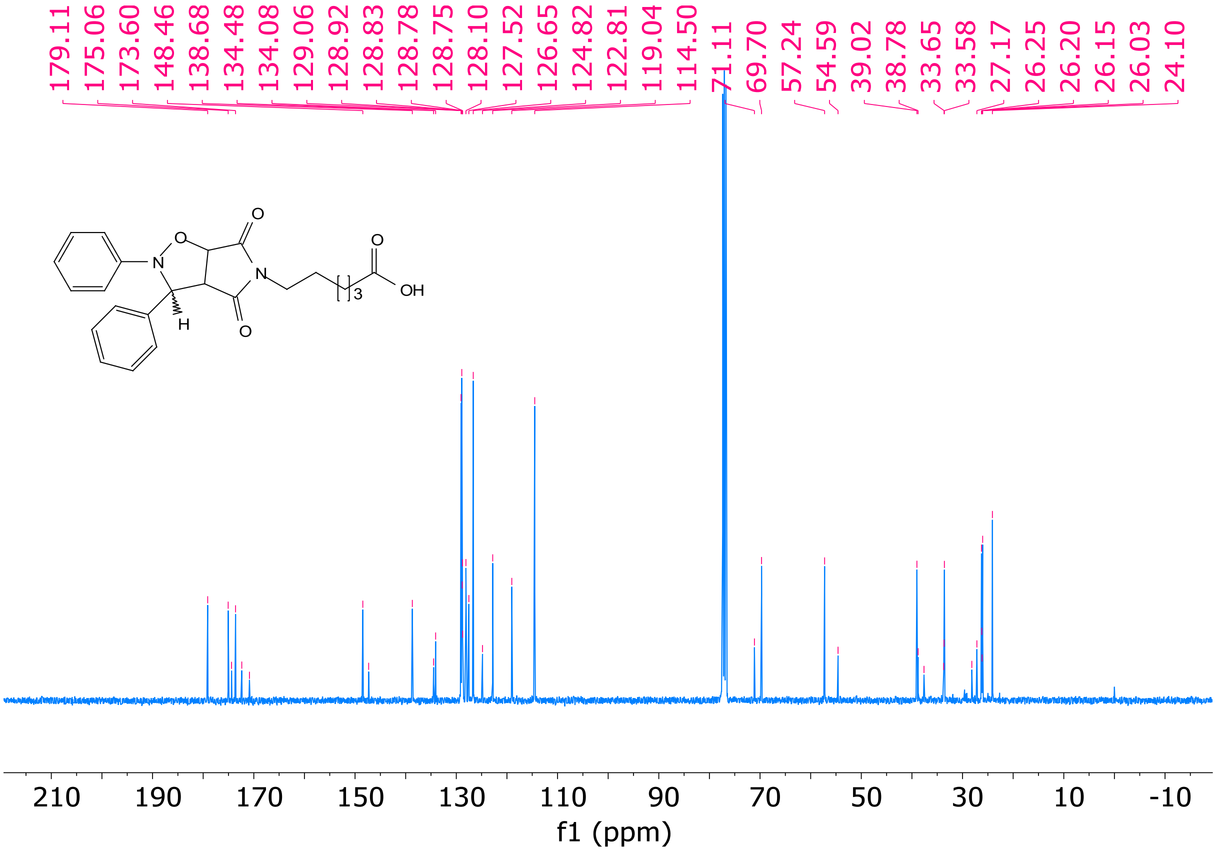
179.11
175.06
173.60
148.46
138.68
134.48
134.08
129.06
128.92
128.83
128.78
128.75
128.10
127.52
126.65
124.82
122.81
119.04
114.50
71.11
69.70
57.24
54.59
39.02
38.78
33.65
33.58
27.17
26.25
26.20
26.15
26.03
24.10
O
N
H
N
O
O
OH
3
210 190 170 150 130 110 90 70 50 30 10 -10
f1 (ppm)

3c

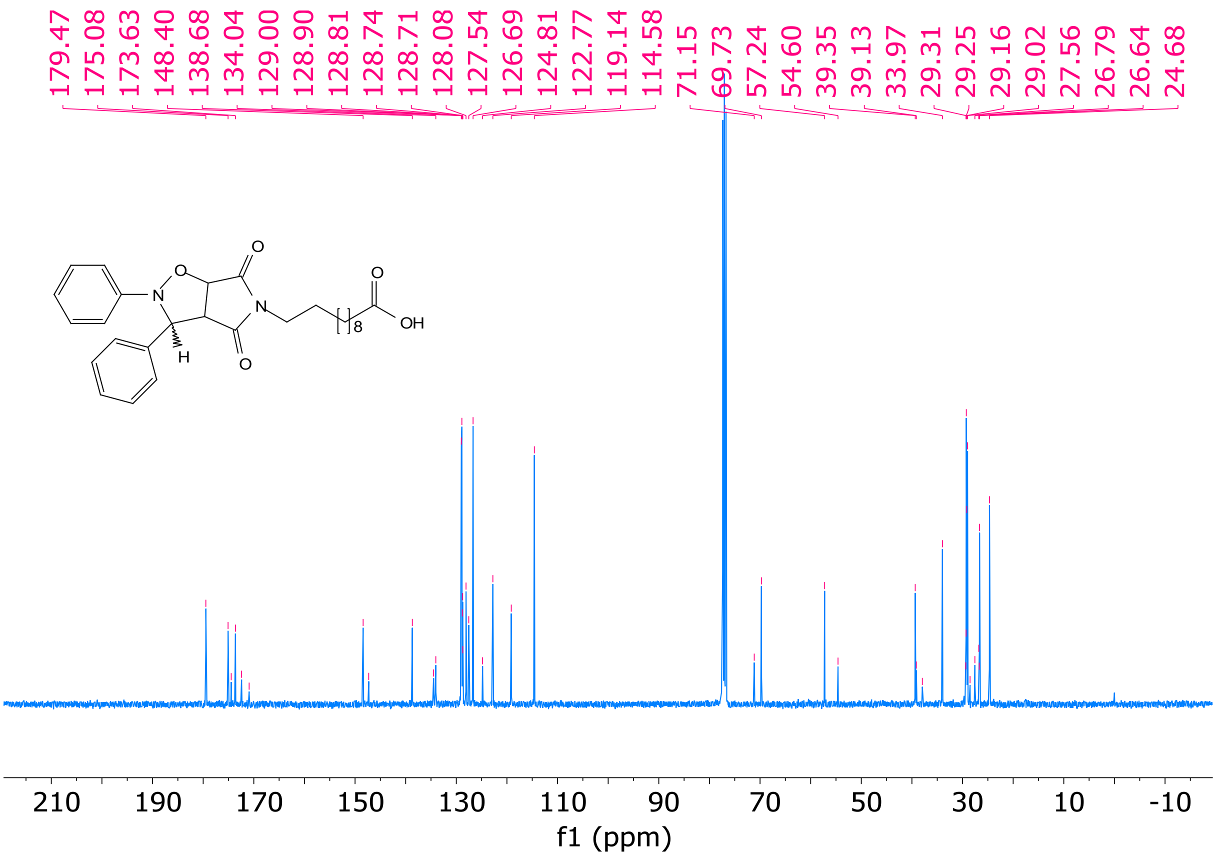
179.47
175.08
173.63
148.40
138.68
134.04
129.00
128.90
128.81
128.74
128.71
128.08
127.54
126.69
124.81
122.77
119.14
114.58
71.15
69.73
57.24
54.60
39.35
39.13
33.97
29.31
29.25
29.16
29.02
27.56
26.79
26.64
24.68
O
N
O
N
H
O
O
8
OH
210
190
170
150
130
110
90
70
50
30
10
-10
f1 (ppm)

3d

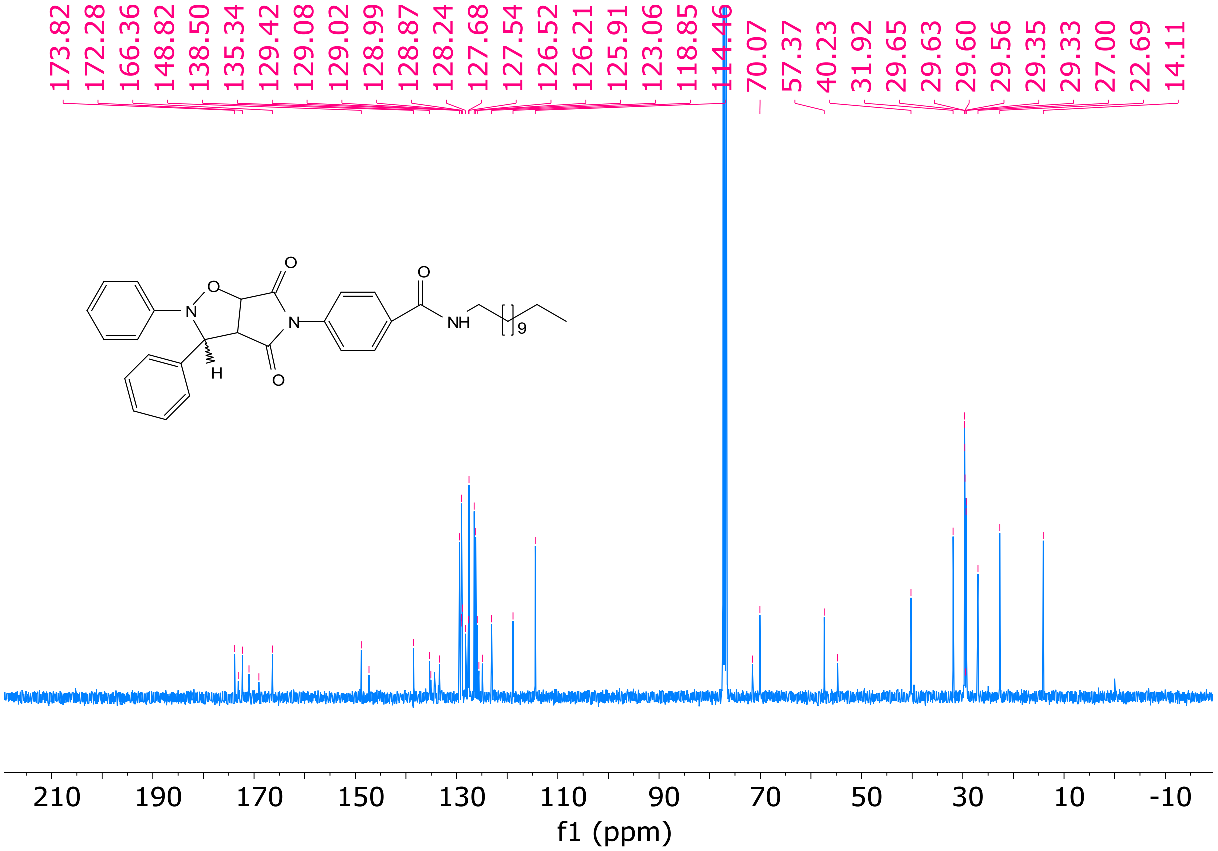
173.82
172.28
166.36
148.82
138.50
135.34
129.42
129.08
129.02
128.99
128.87
128.24
127.68
127.54
126.52
126.21
125.91
123.06
118.85
114.46
70.07
57.37
40.23
31.92
29.65
29.63
29.60
29.56
29.35
29.33
27.00
22.69
14.11
O
O
N
N
O
NH
O
H
9
210
190
170
150
130
110
90
70
50
30
10
-10
f1 (ppm)

3e

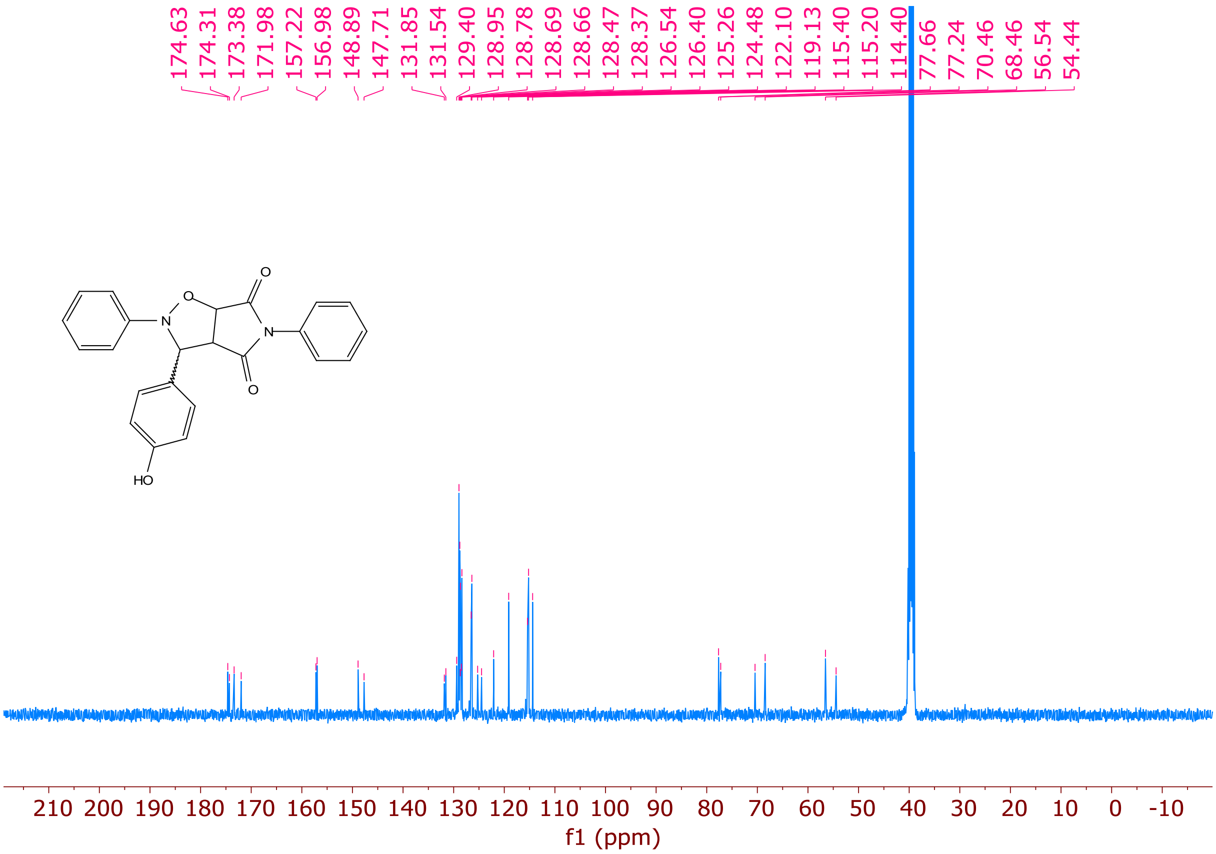
174.63
174.31
173.38
171.98
157.22
156.98
148.89
147.71
131.85
131.54
129.40
128.95
128.78
128.69
128.66
128.47
128.37
126.54
126.40
125.26
124.48
122.10
119.13
115.40
115.20
114.40
77.66
77.24
70.46
68.46
56.54
54.44
O
N
O
N
O
HO
210 200 190 180 170 160 150 140 130 120 110 100 90 80 70 60 50 40 30 20 10 0 -10
f1 (ppm)

3f

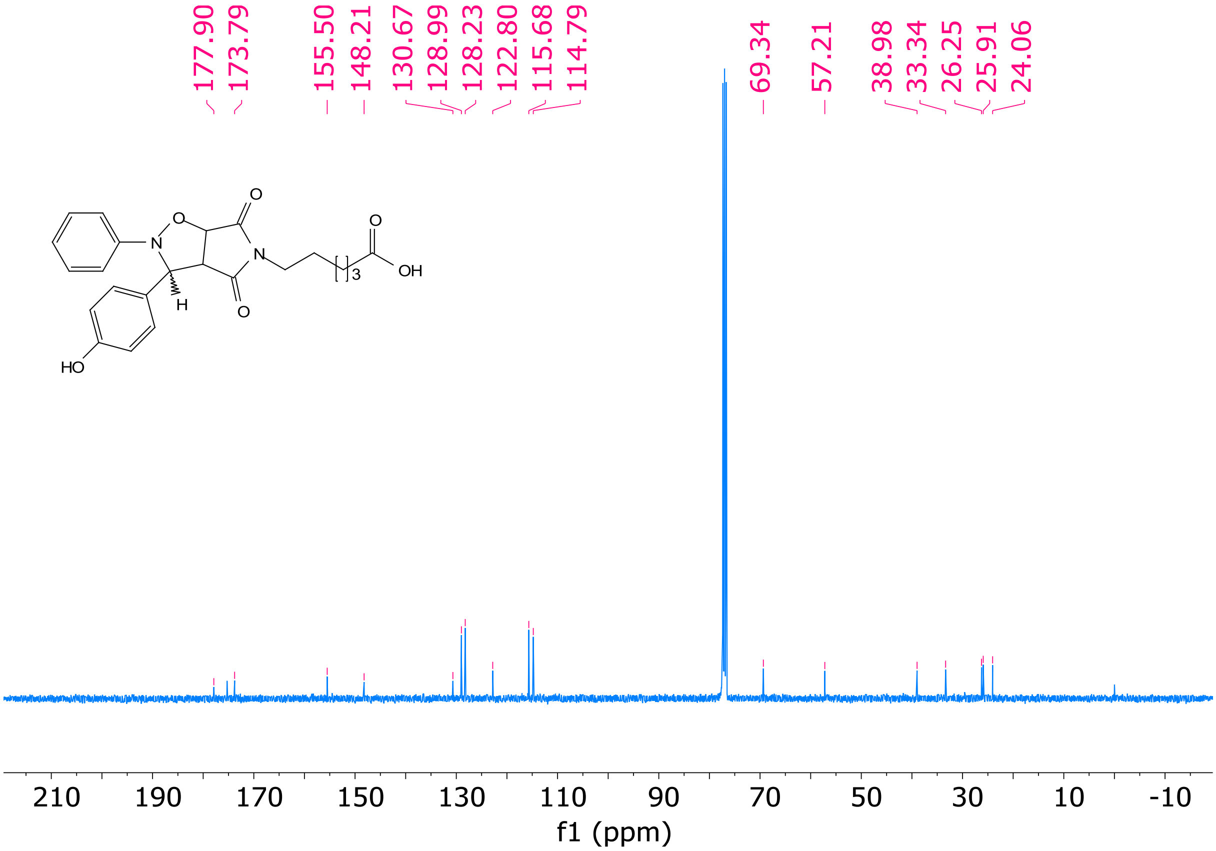

177.90
173.79
155.50
148.21
130.67
128.99
128.23
122.80
115.68
114.79
69.34
57.21
38.98
33.34
26.25
25.91
24.06
O
N
O
N
O
O
OH
3
H
HO
210
190
170
150
130
110
90
70
50
30
10
-10
f1 (ppm)

3g

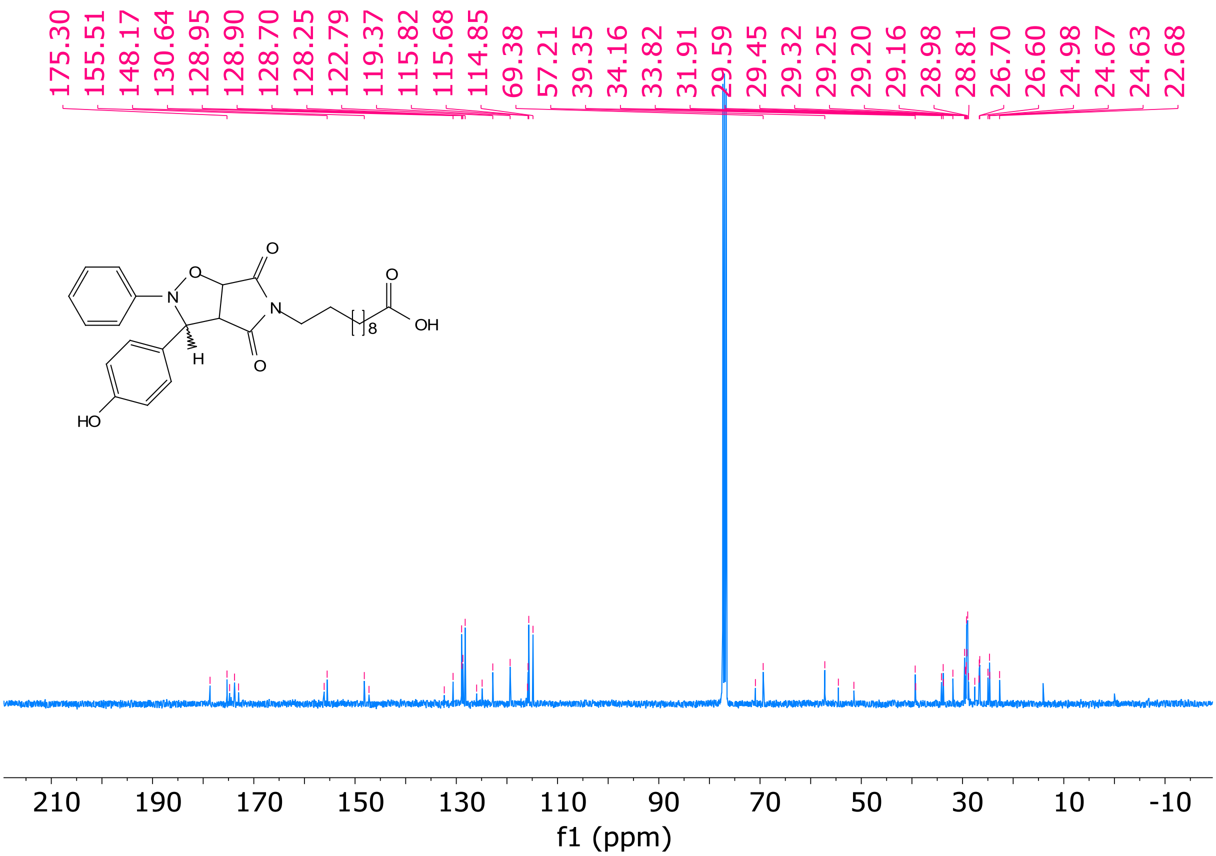
175.30
155.51
148.17
130.64
128.95
128.90
128.70
128.25
122.79
119.37
115.82
115.68
114.85
69.38
57.21
39.35
34.16
33.82
31.91
29.59
29.45
29.32
29.25
29.20
29.16
28.98
28.81
26.70
26.60
24.98
24.67
24.63
22.68
N
O
O
O
N
H
8
O
OH
HO
210
190
170
150
130
110
90
70
50
30
10
-10
f1 (ppm)

3h

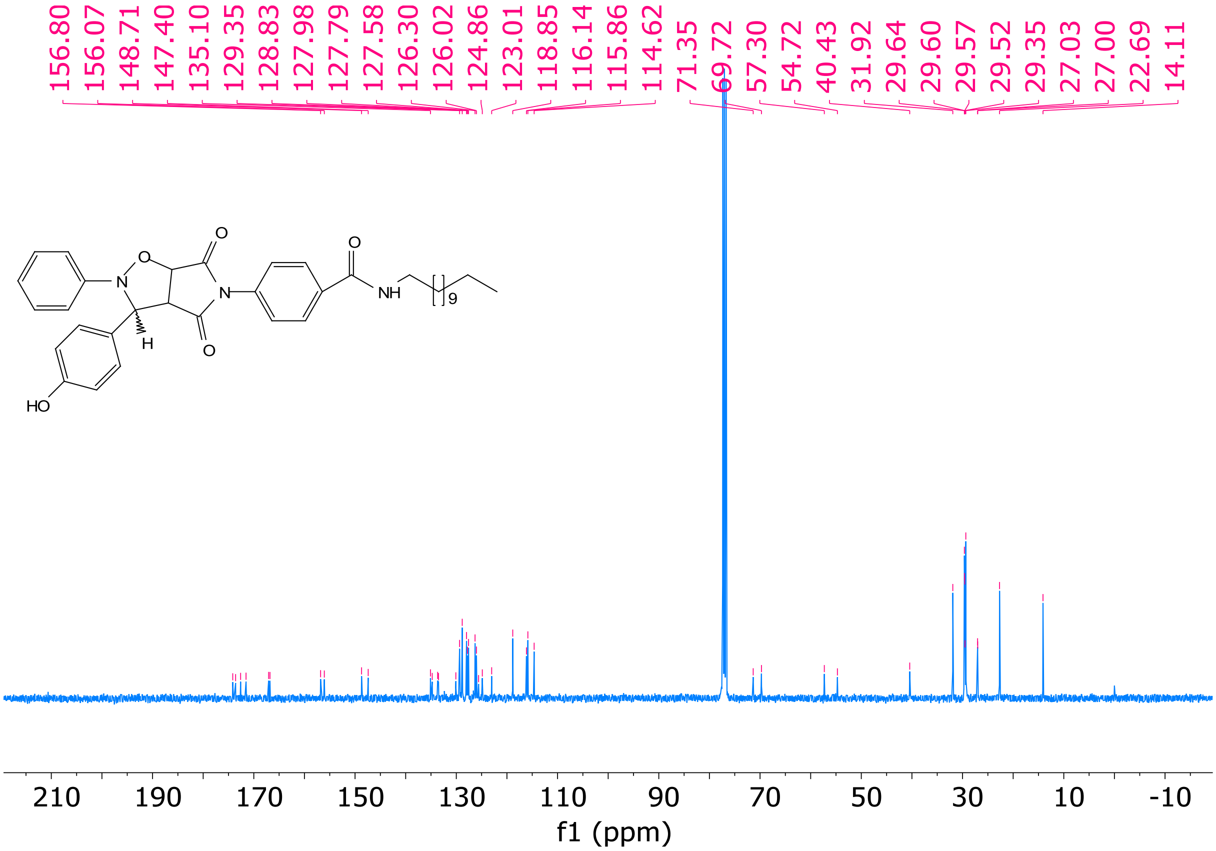

156.80
156.07
148.71
147.40
135.10
129.35
128.83
127.98
127.79
127.58
126.30
126.02
124.86
123.01
118.85
116.14
115.86
114.62
71.35
69.72
57.30
54.72
40.43
31.92
29.64
29.60
29.57
29.52
29.35
27.03
27.00
22.69
14.11
f1 (ppm)

3i

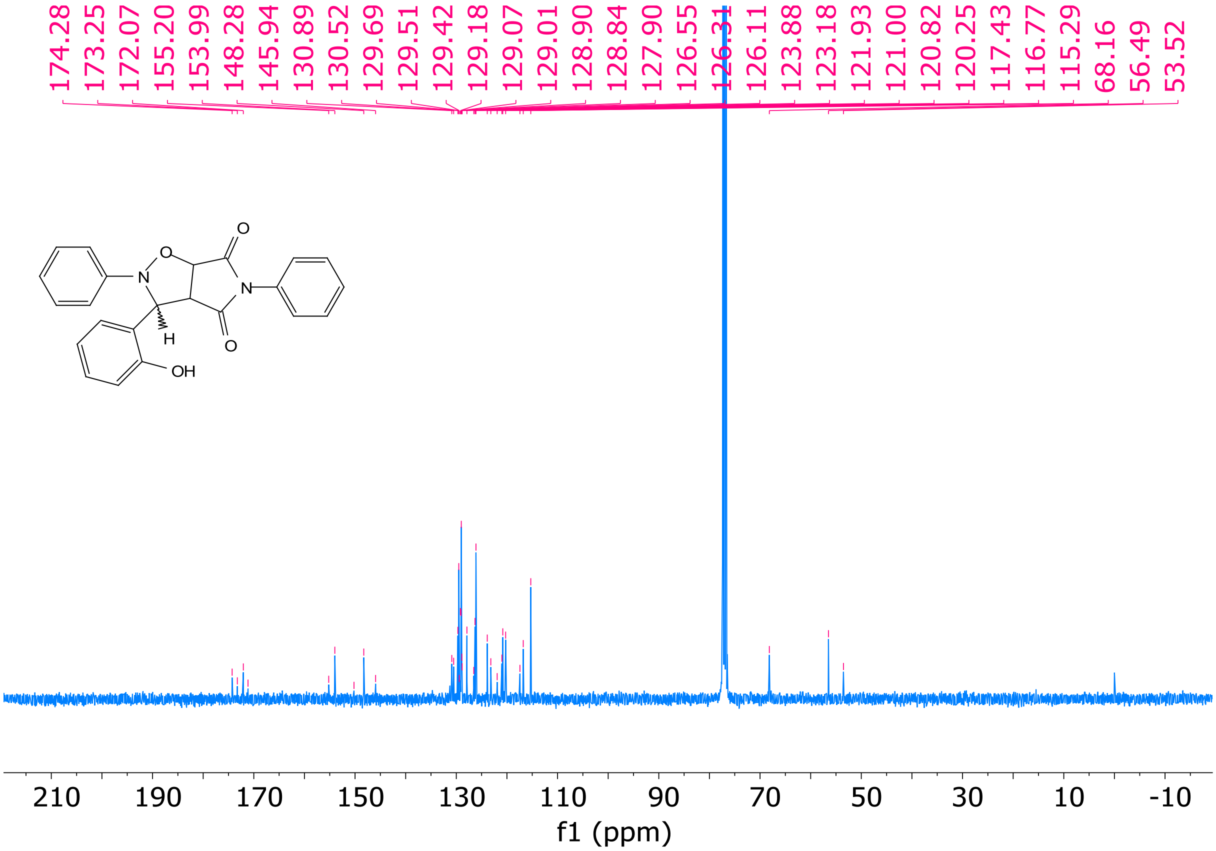

174.28
173.25
172.07
155.20
153.99
148.28
145.94
130.89
130.52
129.69
129.51
129.42
129.18
129.07
129.01
128.90
128.84
127.90
126.55
126.31
126.11
123.88
123.18
121.93
121.00
120.82
120.25
117.43
116.77
115.29
68.16
56.49
53.52
O
N
OH
H
210
190
170
150
130
110
90
70
50
30
10
-10
f1 (ppm)

3j

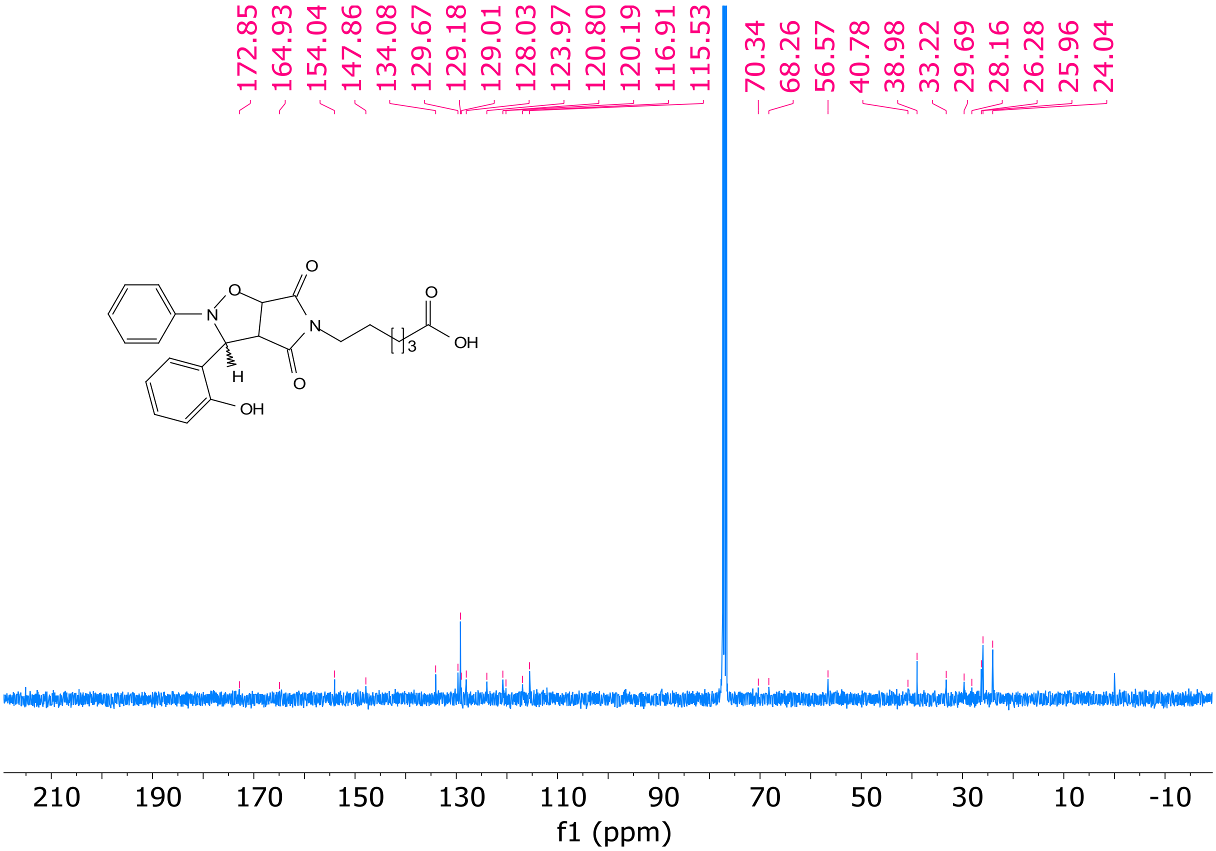
172.85
164.93
154.04
147.86
134.08
129.67
129.18
129.01
128.03
123.97
120.80
120.19
116.91
115.53
70.34
68.26
56.57
40.78
38.98
33.22
29.69
28.16
26.28
25.96
24.04
O
N
O
N
H
OH
O
3
O
OH
210
190
170
150
130
110
90
70
50
30
10
-10
f1 (ppm)

3k

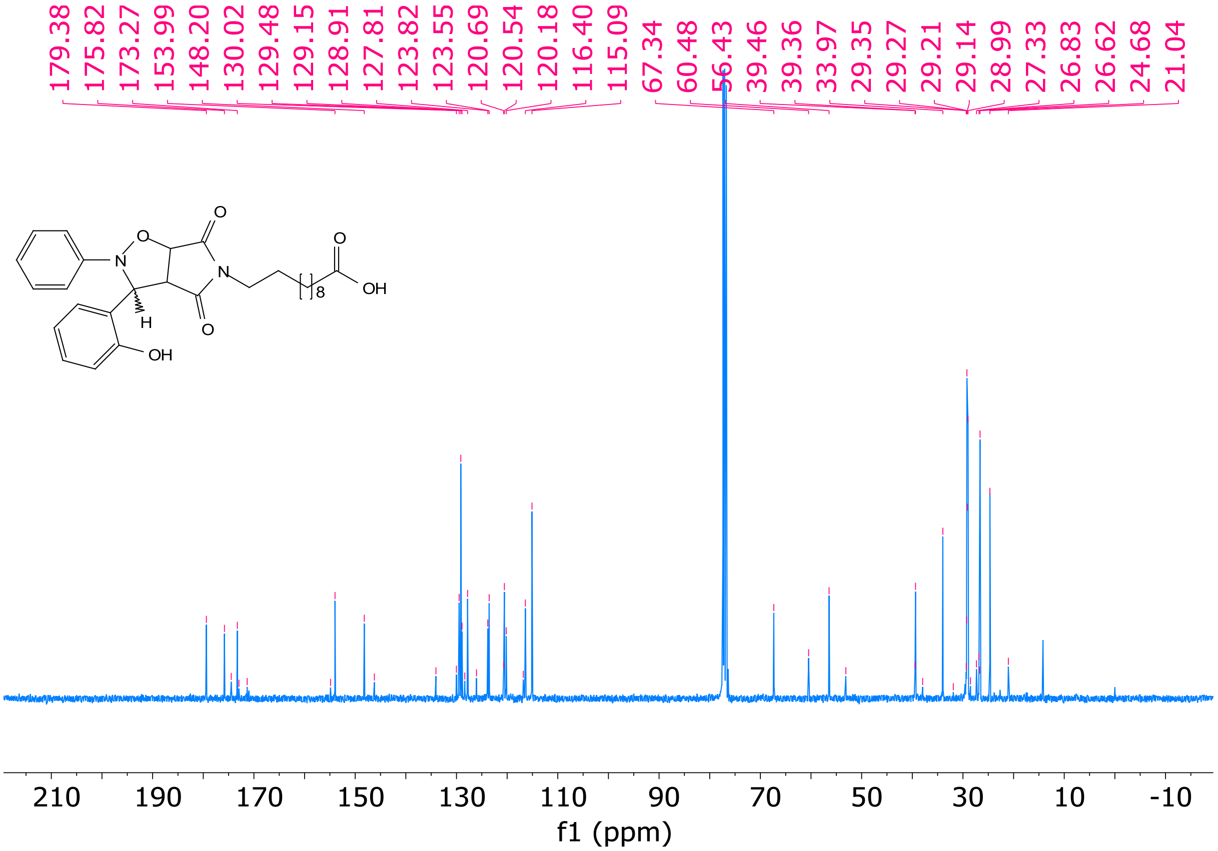

179.38
175.82
173.27
153.99
148.20
130.02
129.48
129.15
128.91
127.81
123.82
123.55
120.69
120.54
120.18
116.40
115.09
67.34
60.48
56.43
39.46
39.36
33.97
29.35
29.27
29.21
29.14
28.99
27.33
26.83
26.62
24.68
21.04
O
N
O
N
H
O
OH
8
OH
O
210
190
170
150
130
110
90
70
50
30
10
-10
f1 (ppm)

3l

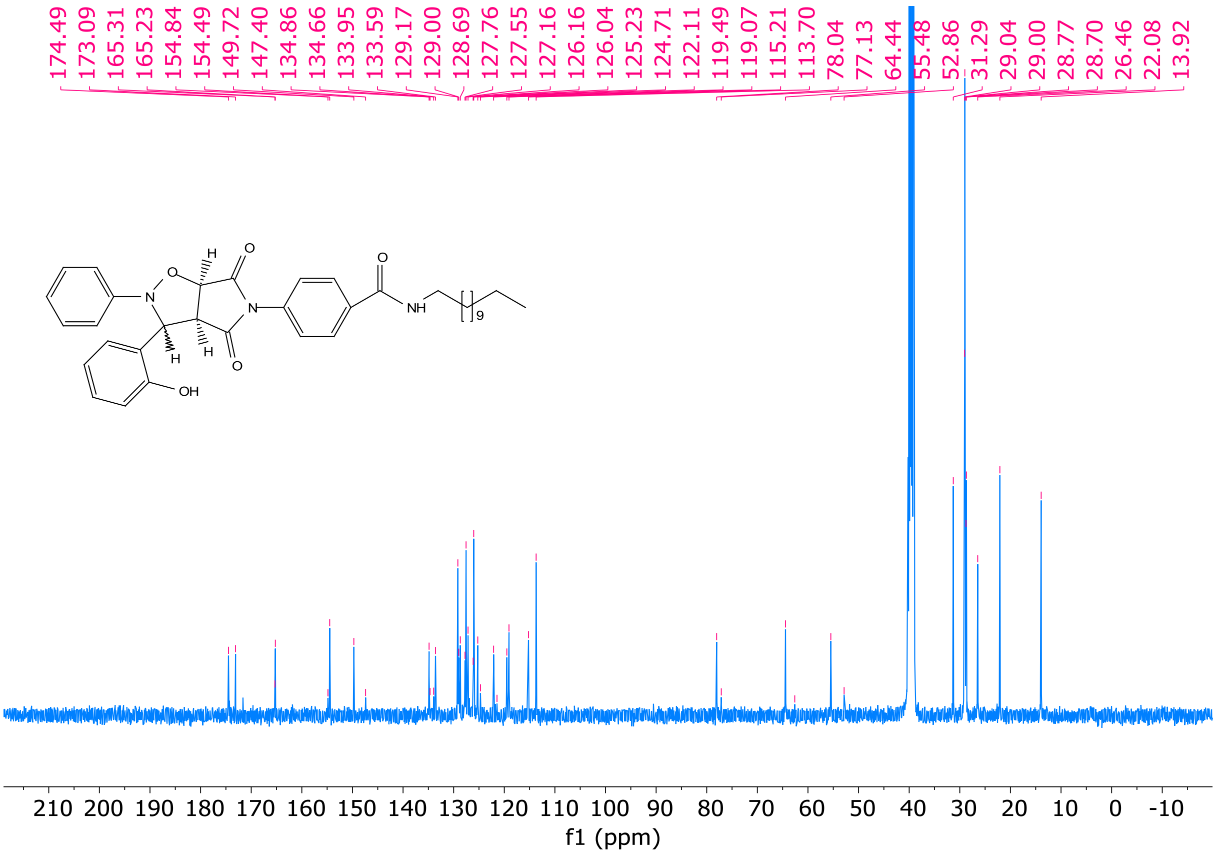
174.49
173.09
165.31
165.23
154.84
154.49
149.72
147.40
134.86
134.66
133.95
133.59
129.17
129.00
128.69
127.76
127.55
127.16
126.16
126.04
125.23
124.71
122.11
119.49
119.07
115.21
113.70
78.04
77.13
64.44
55.48
52.86
31.29
29.04
29.00
28.77
28.70
26.46
22.08
13.92
O
H
O
N
N
H
H
OH
O
O
NH
9
210 200 190 180 170 160 150 140 130 120 110 100 90 80 70 60 50 40 30 20 10 0 -10
f1 (ppm)

3m

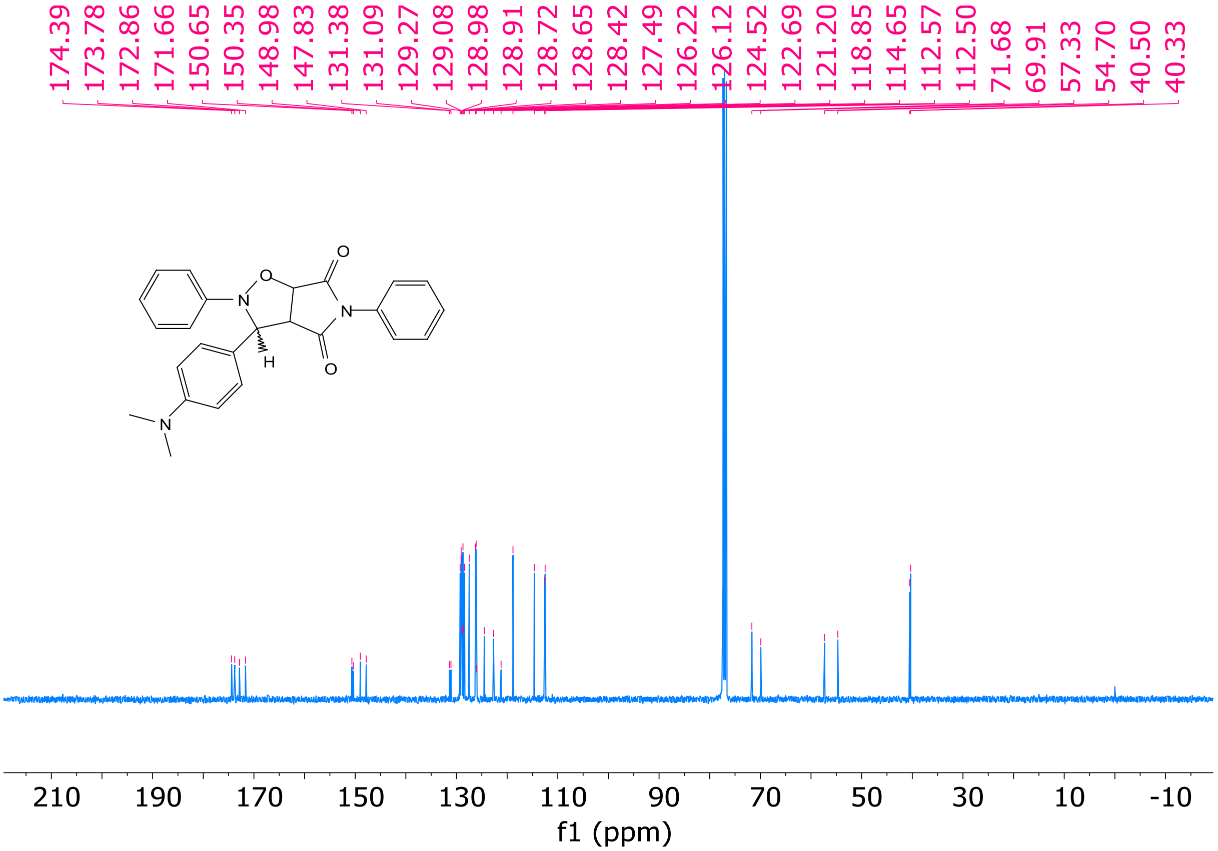

N
O
N
O
O
H
N
174.39
173.78
172.86
171.66
150.65
150.35
148.98
147.83
131.38
131.09
129.27
129.08
128.98
128.91
128.72
128.65
128.42
127.49
126.22
126.12
124.52
122.69
121.20
118.85
114.65
112.57
112.50
71.68
69.91
57.33
54.70
40.50
40.33
210
190
170
150
130
110
90
70
50
30
10
-10
f1 (ppm)

3n

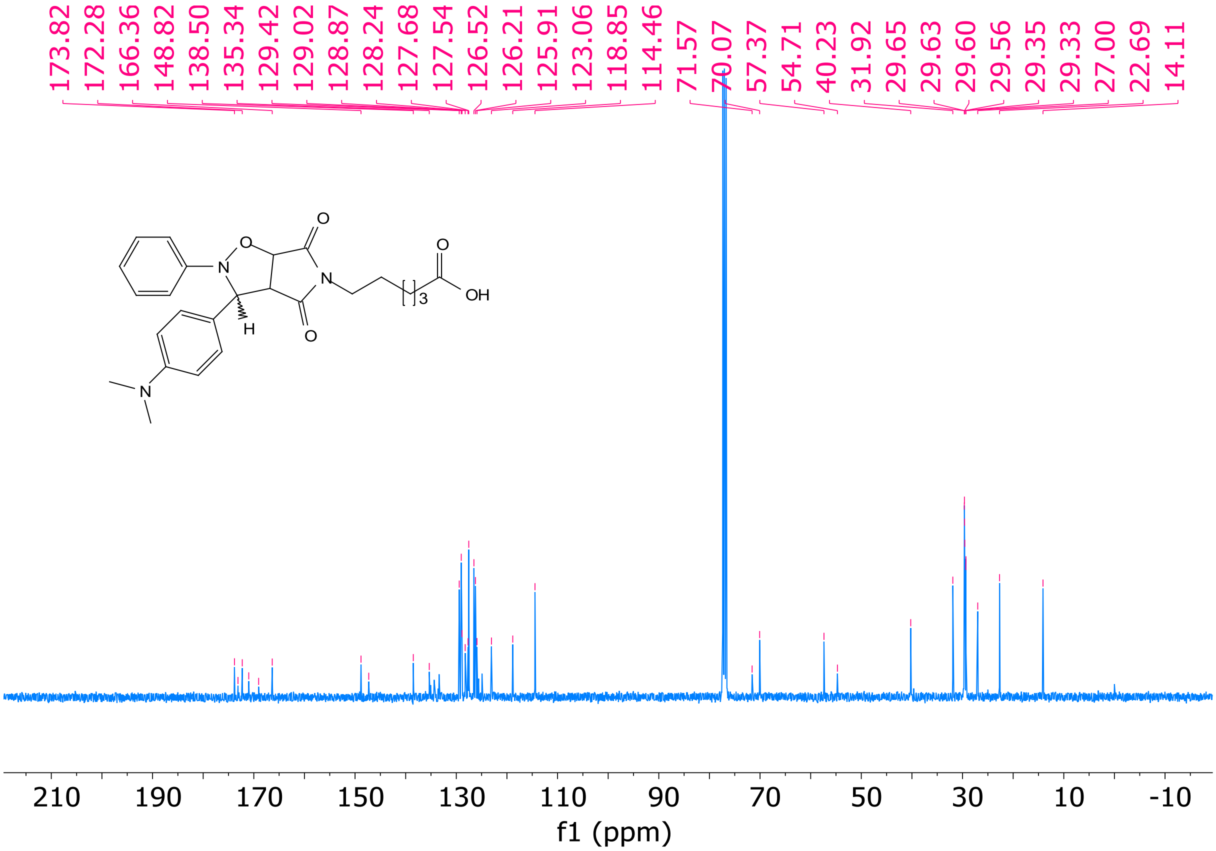

173.82
172.28
166.36
148.82
138.50
135.34
129.42
129.02
128.87
128.24
127.68
127.54
126.52
126.21
125.91
123.06
118.85
114.46
71.57
70.07
57.37
54.71
40.23
31.92
29.65
29.63
29.60
29.56
29.35
29.33
27.00
22.69
14.11
O
N
O
N
O
O
OH
3
H
N
210
190
170
150
130
110
90
70
50
30
10
-10
f1 (ppm)

3o

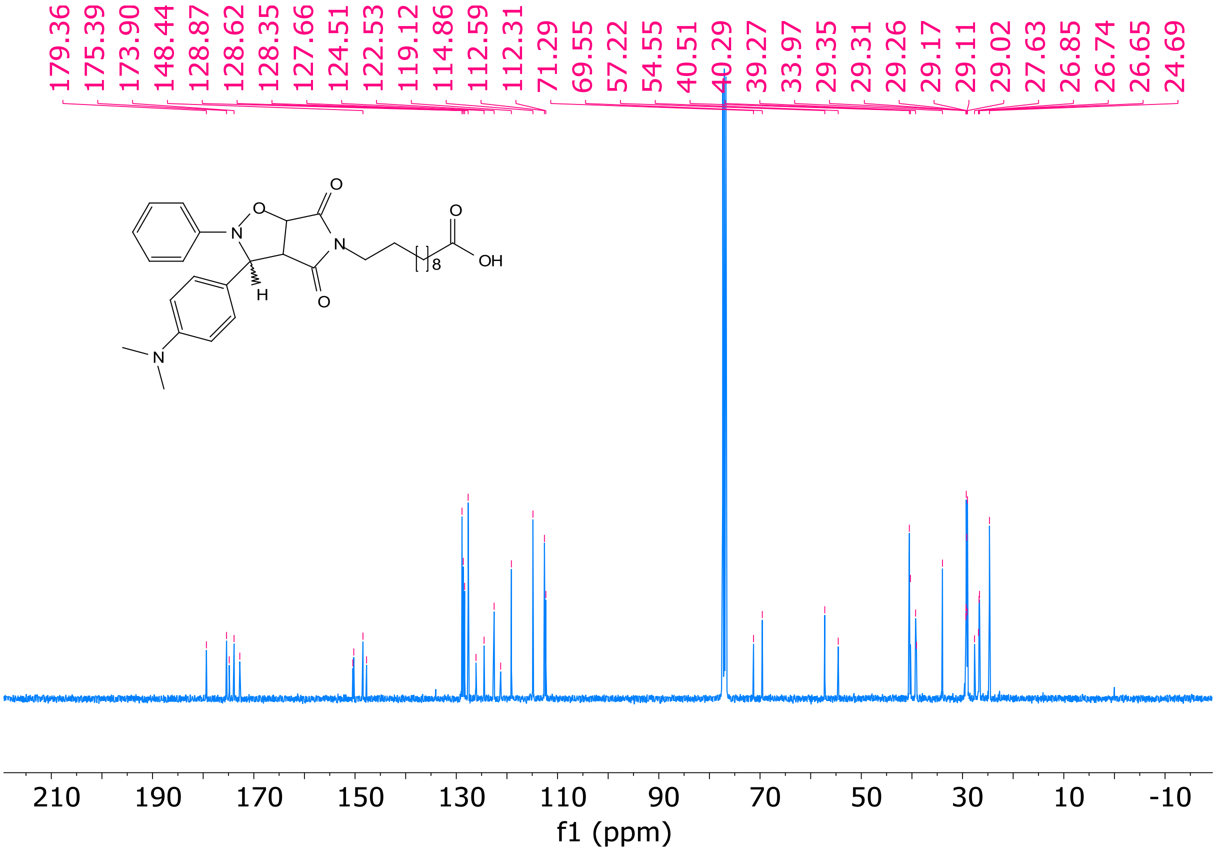
179.36
175.39
173.90
148.44
128.87
128.62
128.35
127.66
124.51
122.53
119.12
114.86
112.59
112.31
71.29
69.55
57.22
54.55
40.51
40.29
39.27
33.97
29.35
29.31
29.26
29.17
29.11
29.02
27.63
26.85
26.74
26.65
24.69
O
N
OH
H
8
210
190
170
150
130
110
90
70
50
30
10
-10
f1 (ppm)

3p

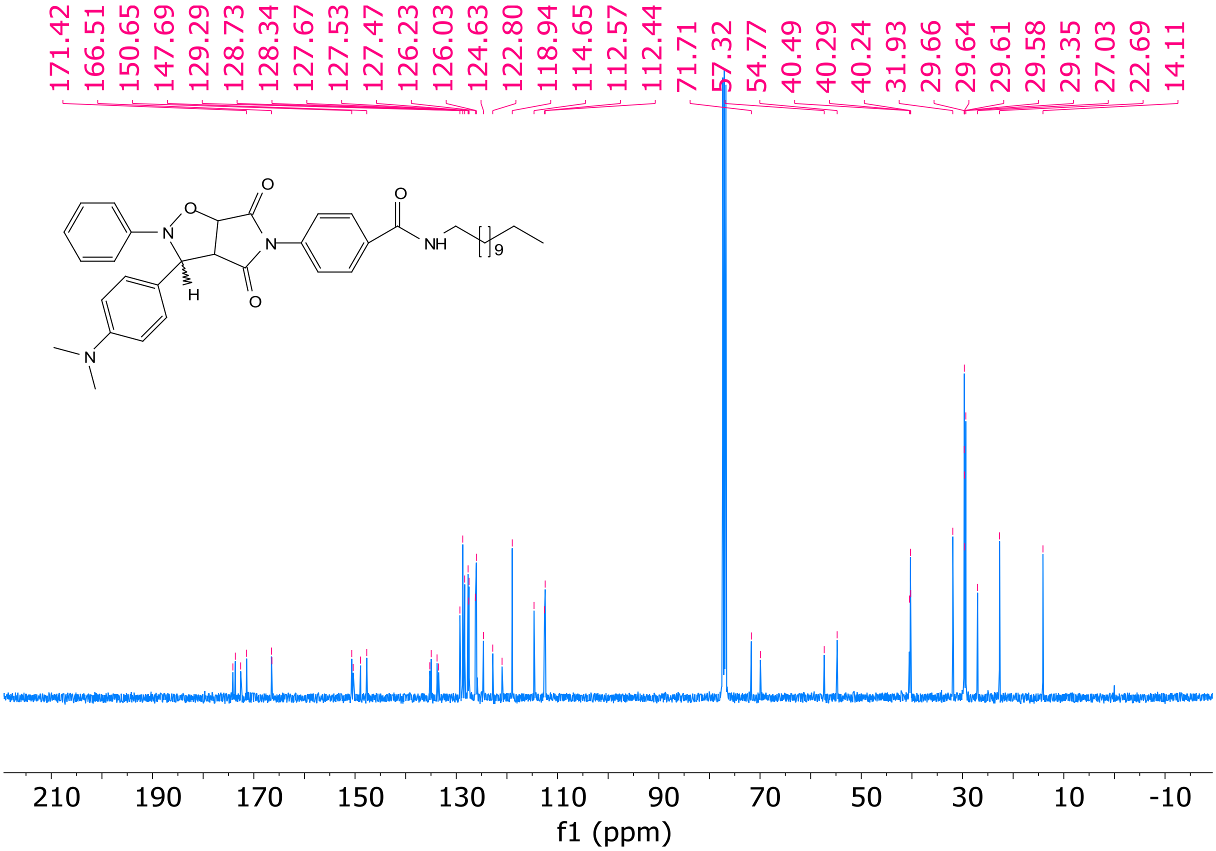
171.42
166.51
150.65
147.69
129.29
128.73
128.34
127.67
127.53
127.47
126.23
126.03
124.63
122.80
118.94
114.65
112.57
112.44
71.71
57.32
54.77
40.49
40.29
40.24
31.93
29.66
29.64
29.61
29.58
29.35
27.03
22.69
14.11
O
N
O
N
O
NH
9
H
N
O
210
190
170
150
130
110
90
70
50
30
10
-10
f1 (ppm)

3q

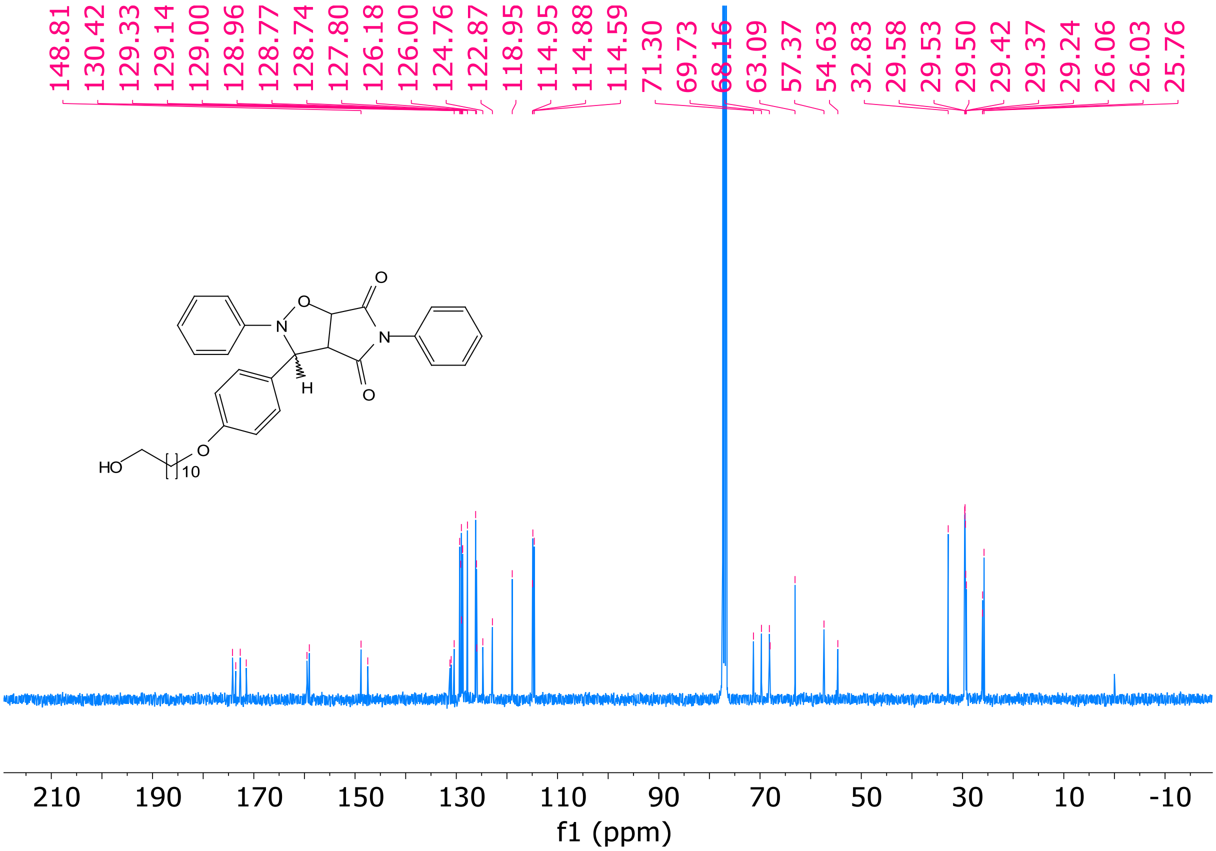
148.81
130.42
129.33
129.14
129.00
128.96
128.77
128.74
127.80
126.18
126.00
124.76
122.87
118.95
114.95
114.88
114.59
71.30
69.73
68.16
63.09
57.37
54.63
32.83
29.58
29.53
29.50
29.42
29.37
29.24
26.06
26.03
25.76
O
N
O
N
O
H
HO
O
10
210
190
170
150
130
110
90
70
50
30
10
-10
f1 (ppm)

3r

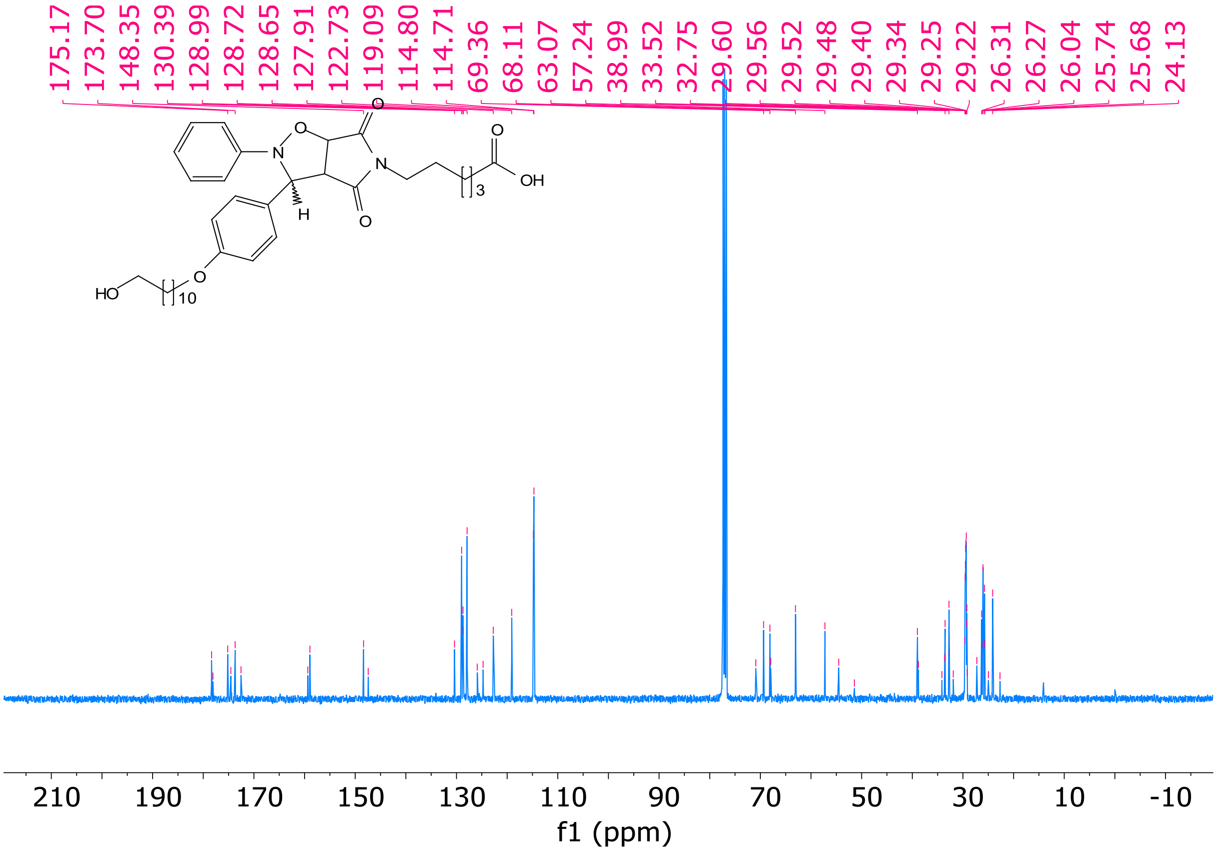
175.17
173.70
148.35
130.39
128.99
128.72
128.65
127.91
122.73
119.09
114.80
114.71
69.36
68.11
63.07
57.24
38.99
33.52
32.75
29.60
29.56
29.52
29.48
29.40
29.34
29.25
29.22
26.31
26.27
26.04
25.74
25.68
24.13
O
O
N
N
O
O
OH
3
H
O
HO
10
210
190
170
150
130
110
90
70
50
30
10
-10
f1 (ppm)

3s

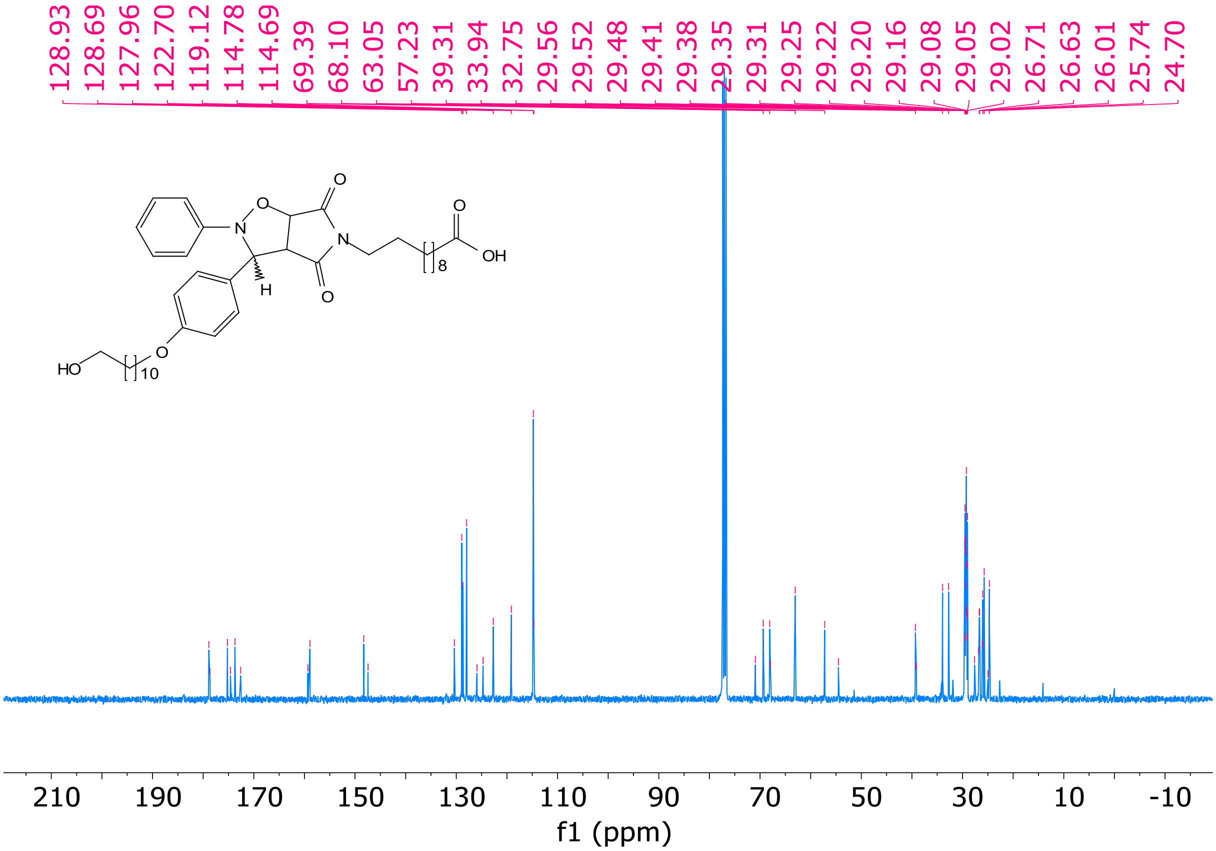
128.93
128.69
127.96
122.70
119.12
114.78
114.69
69.39
68.10
63.05
57.23
39.31
33.94
32.75
29.56
29.52
29.48
29.41
29.38
29.35
29.31
29.25
29.22
29.20
29.16
29.08
29.05
29.02
26.71
26.63
26.01
25.74
24.70
O
N
O
N
O
H
O
OH
8
HO
O
10
210
190
170
150
130
110
90
70
50
30
10
-10
f1 (ppm)

3t

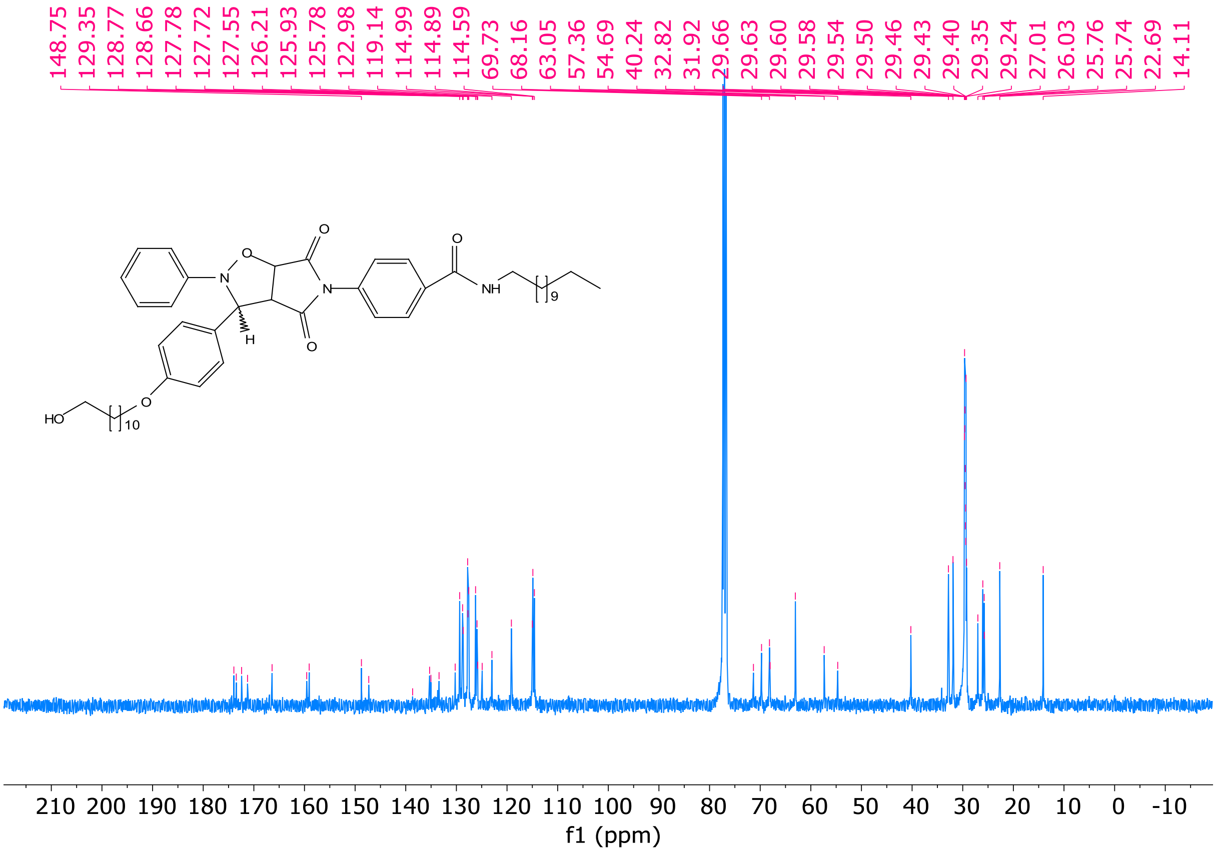

148.75
129.35
128.77
128.66
127.78
127.72
127.55
126.21
125.93
125.78
122.98
119.14
114.99
114.89
114.59
69.73
68.16
63.05
57.36
54.69
40.24
32.82
31.92
29.66
29.63
29.60
29.58
29.54
29.50
29.46
29.43
29.40
29.35
29.24
27.01
26.03
25.76
25.74
22.69
14.11
O
O
O
O
N
N
NH
H
HO
O
9
10
210 200 190 180 170 160 150 140 130 120 110 100 90 80 70 60 50 40 30 20 10 0 -10
f1 (ppm)

3u

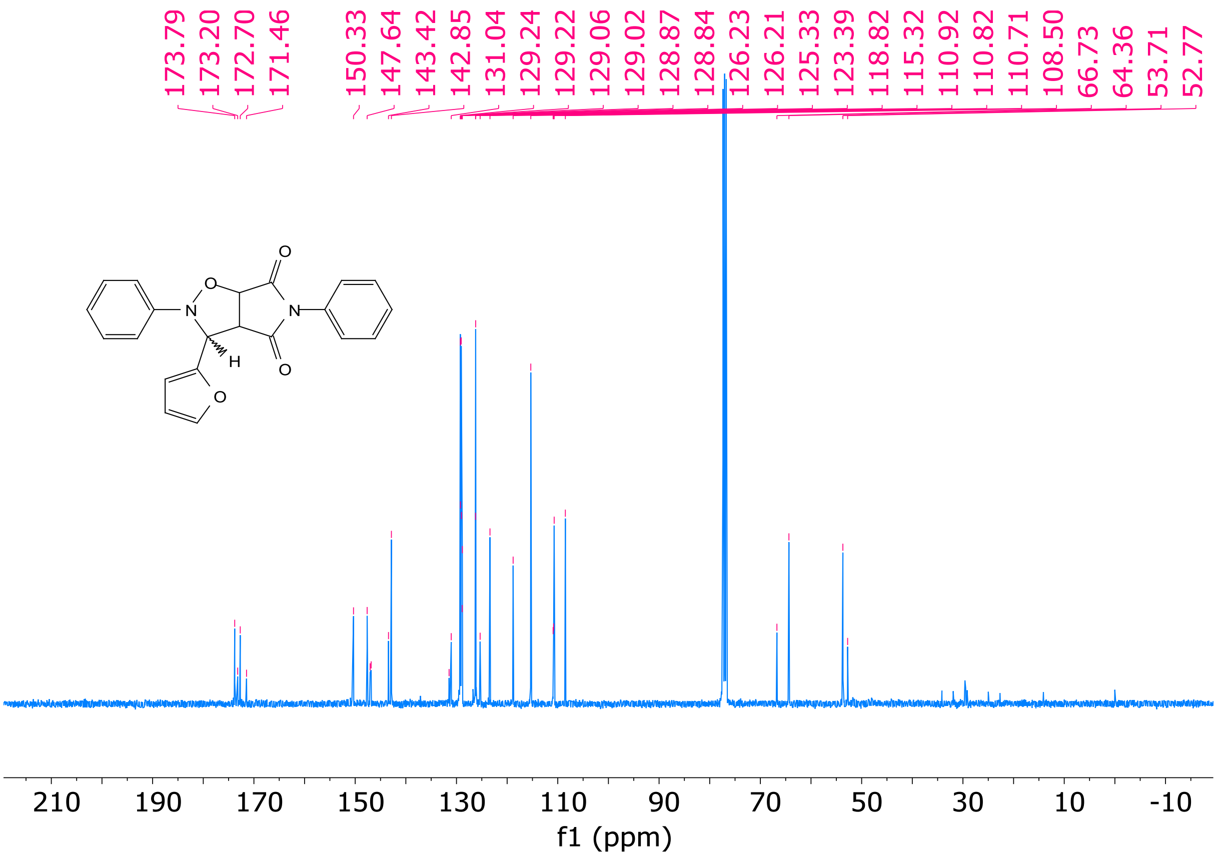
173.79
173.20
172.70
171.46
150.33
147.64
143.42
142.85
131.04
129.24
129.22
129.06
129.02
128.87
128.84
126.23
126.21
125.33
123.39
118.82
115.32
110.92
110.82
110.71
108.50
66.73
64.36
53.71
52.77
O
N
O
N
H
O
O
210
190
170
150
130
110
90
70
50
30
10
-10
f1 (ppm)

Pesticide-likeness

Pesticide-likeness Radar

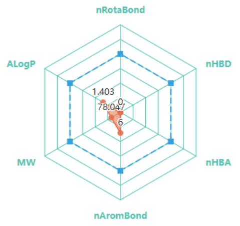

Pesticide-likeness Score

Type	RDL	QEP	GAU
Fungicide Likeness	3.778	0.473	6.326
Herbicide Likeness	4.179	0.396	4.596
Insecticide Likeness	4.581	0.469	3.312

Environmental Risk

nAromBond	6	Biodegradability	NON Readily Biodegradable
Persistence (air)	0.023	Persistence (soil)	22.917
Persistence (water)	7.122	Persistence (sediment)	332

Ecological Risk

Mammals toxicity	Low Toxic
Mallard duck toxicity	High Toxic
Bobwhite quail toxicity	High Toxic
Zebra finch toxicity	Low Toxic
Algae toxicity	4.224
Fish toxicity	3.887
Daphnia toxicity	24.622
Bee toxicity	Non Toxic
Mysid toxicity	5.248
Earthworm toxicity	4.783
Fish BCF	0.983

Figure S1: a) Results for the benzene calculated by *ChemFREE*

Pesticide-likeness

Pesticide-likeness Radar

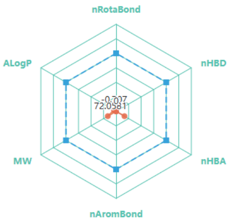

Pesticide-likeness Score

Type	RDL	QEP	GAU
Fungicide Likeness	3.158	0.54	5.734
Herbicide Likeness	3.164	0.347	4.679
Insecticide Likeness	2.993	0.347	3.3

Environmental Risk

nAromBond	0	Biodegradability	Readily Biodegradable
Persistence (air)	0.051	Persistence (soil)	4.954
Persistence (water)	22.997	Persistence (sediment)	229

Ecological Risk

Mammals toxicity	Low Toxic
Mallard duck toxicity	Low Toxic
Bobwhite quail toxicity	High Toxic
Zebra finch toxicity	High Toxic
Algae toxicity	4.694
Fish toxicity	3.086
Daphnia toxicity	97.361
Bee toxicity	Non Toxic
Mysid toxicity	3.985
Earthworm toxicity	8.435
Fish BCF	0.179

Figure S1: b) Results for the THF calculated by *ChemFREE*

Pesticide-likeness

Pesticide-likeness Radar

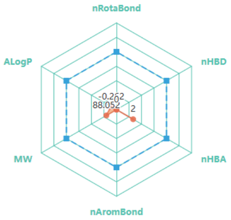

Pesticide-likeness Score

Type	RDL	QEP	GAU
Fungicide Likeness	1.795	0.649	5.719
Herbicide Likeness	3.193	0.383	5.306
Insecticide Likeness	3.135	0.436	4.332

Environmental Risk

nAromBond	0	Biodegradability	Readily Biodegradable
Persistence (air)	0.028	Persistence (soil)	16.207
Persistence (water)	22.997	Persistence (sediment)	127

Ecological Risk

Mammals toxicity	Low Toxic
Mallard duck toxicity	Low Toxic
Bobwhite quail toxicity	High Toxic
Zebra finch toxicity	Low Toxic
Algae toxicity	4.405
Fish toxicity	2.376
Daphnia toxicity	110.881
Bee toxicity	Non Toxic
Mysid toxicity	4.053
Earthworm toxicity	4.127
Fish BCF	0.111

Figure S1: c) Results for the dioxane calculated by *ChemFREE*

Pesticide-likeness

Pesticide-likeness Radar

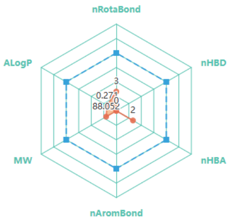

Pesticide-likeness Score

Type	RDL	QEP	GAU
Fungicide Likeness	1.915	0.693	5.461
Herbicide Likeness	2.699	0.444	5.575
Insecticide Likeness	2.483	0.574	4.878

Environmental Risk

nAromBond	0	Biodegradability	Readily Biodegradable
Persistence (air)	0.015	Persistence (soil)	10.672
Persistence (water)	2.883	Persistence (sediment)	23

Ecological Risk

Mammals toxicity	Low Toxic
Mallard duck toxicity	Low Toxic
Bobwhite quail toxicity	High Toxic
Zebra finch toxicity	High Toxic
Algae toxicity	3.334
Fish toxicity	3.47
Daphnia toxicity	314.496
Bee toxicity	Non Toxic
Mysid toxicity	4.992
Earthworm toxicity	40.414
Fish BCF	0.251

Figure S1: d) Results for the EtOAc calculated by *ChemFREE*

Pesticide-likeness

Pesticide-likeness Radar

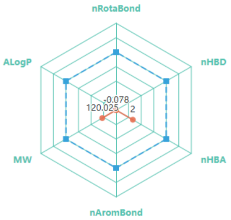

Pesticide-likeness Score

Type	RDL	QEP	GAU
Fungicide Likeness	1.795	0.556	5.54
Herbicide Likeness	2.186	0.429	5.277
Insecticide Likeness	2.415	0.349	4.406

Environmental Risk

nAromBond	0	Biodegradability	Readily Biodegradable
Persistence (air)	0.00311	Persistence (soil)	40.904
Persistence (water)	6.197	Persistence (sediment)	46

Ecological Risk

Mammals toxicity	Low Toxic
Mallard duck toxicity	Low Toxic
Bobwhite quail toxicity	High Toxic
Zebra finch toxicity	Low Toxic
Algae toxicity	3.524
Fish toxicity	3.58
Daphnia toxicity	37.980
Bee toxicity	Non Toxic
Mysid toxicity	4.216
Earthworm toxicity	84.395
Fish BCF	0.064

Figure S1: e) Results for the sulfolane calculated by *ChemFREE*

Pesticide-likeness

Pesticide-likeness Radar

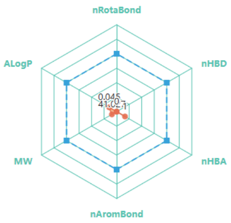

Pesticide-likeness Score

Type	RDL	QEP	GAU
Fungicide Likeness	3.465	0.598	6.156
Herbicide Likeness	2.847	0.368	5.025
Insecticide Likeness	3.445	0.425	3.125

Environmental Risk

nAromBond	0	Biodegradability	Readily Biodegradable
Persistence (air)	0.00201	Persistence (soil)	22.917
Persistence (water)	22.997	Persistence (sediment)	46

Ecological Risk

Mammals toxicity	Low Toxic
Mallard duck toxicity	High Toxic
Bobwhite quail toxicity	High Toxic
Zebra finch toxicity	Low Toxic
Algae toxicity	NaN
Fish toxicity	3.596
Daphnia toxicity	28.124
Bee toxicity	Toxic
Mysid toxicity	5.074
Earthworm toxicity	16.803
Fish BCF	0.250

Figure S1: f) Results for the MeCN calculated by *ChemFREE*

Pesticide-likeness

Pesticide-likeness Radar

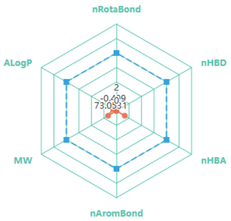

Pesticide-likeness Score

Type	RDL	QEP	GAU
Fungicide Likeness	2.734	0.584	5.77
Herbicide Likeness	2.48	0.382	5.415
Insecticide Likeness	1.866	0.413	3.438

Environmental Risk

nAromBond	0	Biodegradability	NON Readily Biodegradable
Persistence (air)	0.013	Persistence (soil)	4.954
Persistence (water)	4.191	Persistence (sediment)	46

Ecological Risk

Mammals toxicity	Low Toxic
Mallard duck toxicity	High Toxic
Bobwhite quail toxicity	High Toxic
Zebra finch toxicity	High Toxic
Algae toxicity	4.009
Fish toxicity	2.644
Daphnia toxicity	387.173
Bee toxicity	Toxic
Mysid toxicity	4.84
Earthworm toxicity	25.959
Fish BCF	0.103

Figure S1: g) Results for the DMFcalculated by *ChemFREE*

Pesticide-likeness

Pesticide-likeness Radar

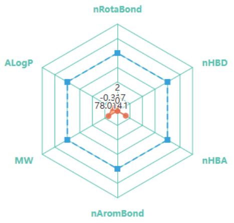

Pesticide-likeness Score

Type	RDL	QEP	GAU
Fungicide Likeness	2.734	0.614	5.507
Herbicide Likeness	2.48	0.392	4.746
Insecticide Likeness	1.866	0.442	3.571

Environmental Risk

nAromBond	0	Biodegradability	Readily Biodegradable
Persistence (air)	0.021	Persistence (soil)	22.917
Persistence (water)	2.529	Persistence (sediment)	46

Ecological Risk

Mammals toxicity	Low Toxic
Mallard duck toxicity	High Toxic
Bobwhite quail toxicity	High Toxic
Zebra finch toxicity	Low Toxic
Algae toxicity	5.023
Fish toxicity	3.359
Daphnia toxicity	9763.896
Bee toxicity	Non Toxic
Mysid toxicity	4.361
Earthworm toxicity	80.385
Fish BCF	0.116

Figure S1: h) Results for the DMSO calculated by *ChemFREE*

Pesticide-likeness

Pesticide-likeness Radar

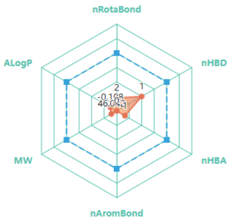

Pesticide-likeness Score

Type	RDL	QEP	GAU
Fungicide Likeness	2.639	0.521	5.588
Herbicide Likeness	1.963	0.38	5.311
Insecticide Likeness	1.543	0.396	3.563

Environmental Risk

nAromBond	0	Biodegradability	NON Readily Biodegradable
Persistence (air)	0.00703	Persistence (soil)	4.954
Persistence (water)	22.997	Persistence (sediment)	46

Ecological Risk

Mammals toxicity	Low Toxic
Mallard duck toxicity	High Toxic
Bobwhite quail toxicity	High Toxic
Zebra finch toxicity	Low Toxic
Algae toxicity	5.311
Fish toxicity	2.83
Daphnia toxicity	439.857
Bee toxicity	Non Toxic
Mysid toxicity	4.817
Earthworm toxicity	126.170
Fish BCF	0.233

Figure S1: i) Results for the EtOH calculated by *ChemFREE*

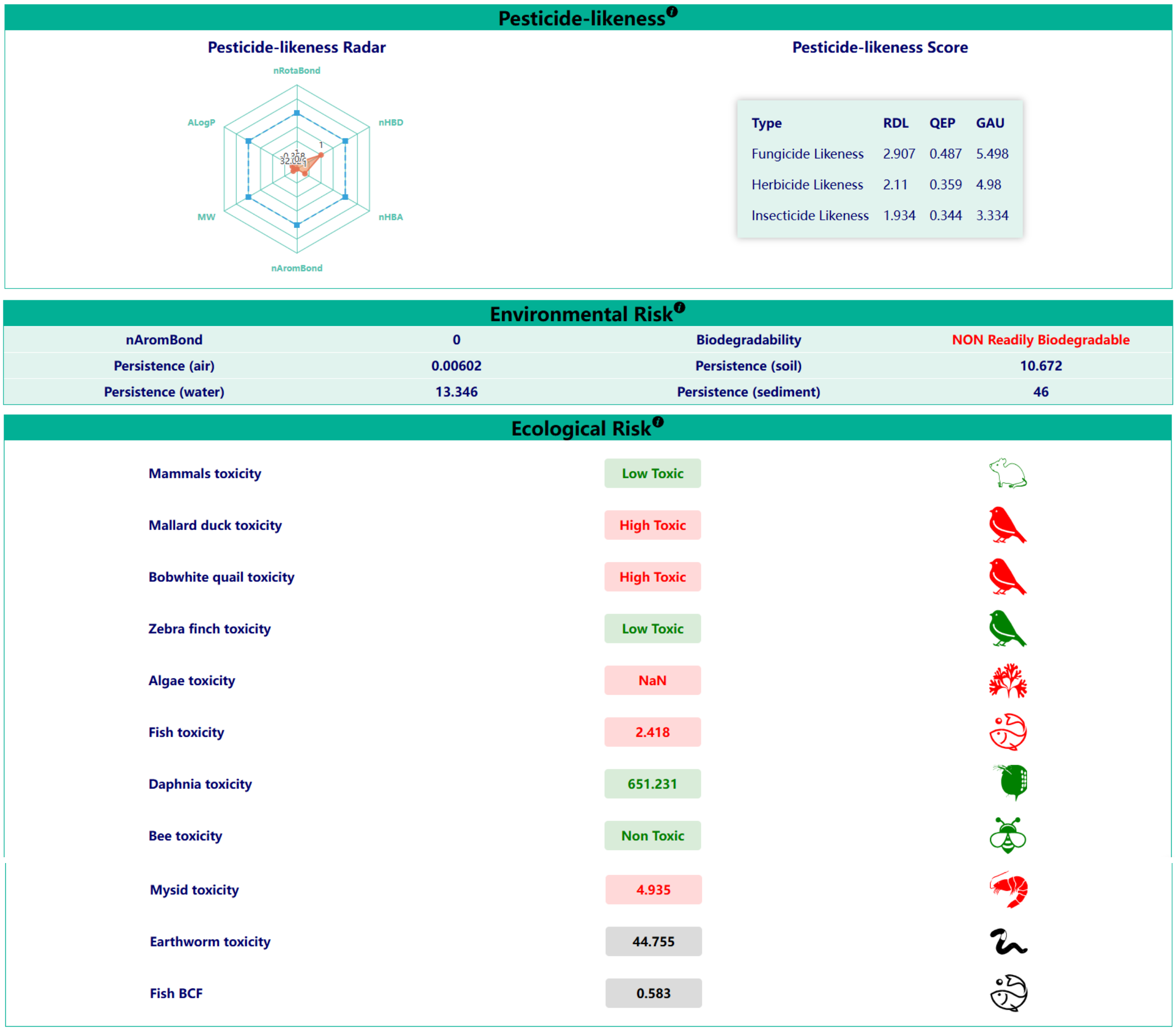

Type	RDL	QEP	GAU
Fungicide Likeness	2.907	0.487	5.498
Herbicide Likeness	2.11	0.359	4.98
Insecticide Likeness	1.934	0.344	3.334

Environmental Risk

nAromBond	0	Biodegradability	NON Readily Biodegradable
Persistence (air)	0.00602	Persistence (soil)	10.672
Persistence (water)	13.346	Persistence (sediment)	46

Ecological Risk

Mammals toxicity	Low Toxic
Mallard duck toxicity	High Toxic
Bobwhite quail toxicity	High Toxic
Zebra finch toxicity	Low Toxic
Algae toxicity	NaN
Fish toxicity	2.418
Daphnia toxicity	651.231
Bee toxicity	Non Toxic
Mysid toxicity	4.935
Earthworm toxicity	44.755
Fish BCF	0.583

Figure S1: j) Results for the MeOH calculated by *ChemFREE*

Pesticide-likeness

Pesticide-likeness Radar

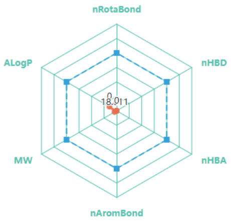

Pesticide-likeness Score

Type	RDL	QEP	GAU
Fungicide Likeness	0.0	0.436	5.626
Herbicide Likeness	0.0	0.317	4.314
Insecticide Likeness	0.0	0.385	3.254

Environmental Risk

nAromBond	0	Biodegradability	NON Readily Biodegradable
Persistence (air)	0.00676	Persistence (soil)	Not classifiable
Persistence (water)	15.794	Persistence (sediment)	Not classifiable

Ecological Risk

Mammals toxicity	Low Toxic
Mallard duck toxicity	High Toxic
Bobwhite quail toxicity	High Toxic
Zebra finch toxicity	High Toxic
Algae toxicity	NaN
Fish toxicity	3.412
Daphnia toxicity	1119.788
Bee toxicity	Low Toxic
Mysid toxicity	5.326
Earthworm toxicity	Not classifiable
Fish BCF	Not classifiable

Figure S1: k) Results for the water calculated by *ChemFREE*

Pesticide-likeness

Pesticide-likeness Radar

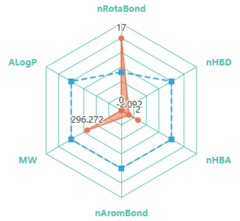

Pesticide-likeness Score

Type	RDL	QEP	GAU
Fungicide Likeness	0.0	0.0	4.74
Herbicide Likeness	0.0	0.386	4.764
Insecticide Likeness	0.0	0.323	4.84

Environmental Risk

nAromBond	0	Biodegradability	Readily Biodegradable
Persistence (air)	1.019	Persistence (soil)	22.918
Persistence (water)	7.075	Persistence (sediment)	13

Ecological Risk

Mammals toxicity	Low Toxic
Mallard duck toxicity	Low Toxic
Bobwhite quail toxicity	Low Toxic
Zebra finch toxicity	High Toxic
Algae toxicity	7.003
Fish toxicity	5.843
Daphnia toxicity	0.28840
Bee toxicity	Non Toxic
Mysid toxicity	6.773
Earthworm toxicity	50.969
Fish BCF	1.633

Figure S1: l) Results for the methyl oleate calculated by *ChemFREE*

Pesticide-likeness

Pesticide-likeness Radar

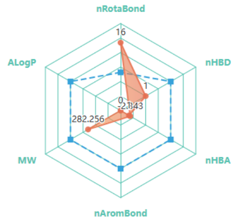

Pesticide-likeness Score

Type	RDL	QEP	GAU
Fungicide Likeness	0.734	0.168	4.832
Herbicide Likeness	0.0	0.36	5.139
Insecticide Likeness	0.0	0.267	4.526

Environmental Risk

nAromBond	0	Biodegradability	Readily Biodegradable
Persistence (air)	0.837	Persistence (soil)	22.918
Persistence (water)	1071.741	Persistence (sediment)	13

Ecological Risk

Mammals toxicity	Low Toxic
Mallard duck toxicity	Low Toxic
Bobwhite quail toxicity	Low Toxic
Zebra finch toxicity	High Toxic
Algae toxicity	7.367
Fish toxicity	5.657
Daphnia toxicity	0.28962
Bee toxicity	Non Toxic
Mysid toxicity	5.8
Earthworm toxicity	83.958
Fish BCF	1.860

Figure S1: m) Results for the oleic acid calculated by *ChemFREE*

Table 1S: Polarizabilities of the solvents.[a]

Entry	Solvent	Displaying	Molecular polarizability	Principal polarizability components		
				α(xx)	α(yy)	α(zz)
1	Benzene	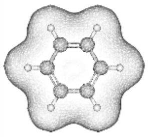	8.89	10.74	10.47	5.19
2	Toluene	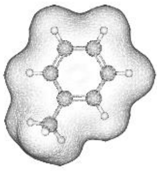	10.97	12.08	14.05	6.79
3	Chloroform	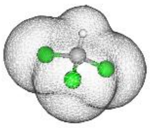	8.52	9.34	9.34	6.89
4	THF	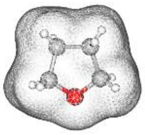	8.14	8.16	9.11	7.15
5	Dioxane	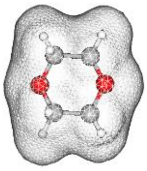	8.97	7.58	8.78	10.55
6	EtOAc	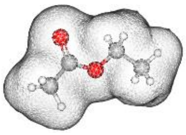	9.38	8.82	7.39	11.91
7	Sulfolane	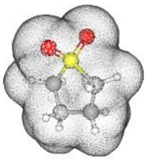	11.48	11.37	10.36	12.70
8	MeCN	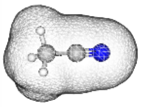	4.27	5.51	3.65	3.65
9	DMF	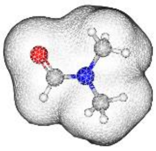	7.69	8.29	8.82	5.95
10	DMSO	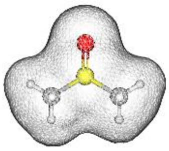	7.91	9.04	8.08	6.60
11	EtOH	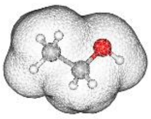	5.30	6.37	4.91	4.63

12	MeOH	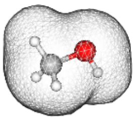	3.38	3.01	3.18	3.96
13	Water	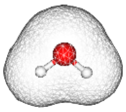	1.51	1.91	1.40	1.21
14	Methyl oleate	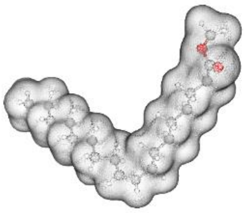	39.08	41.85	46.24	29.16
15	Oleic acid	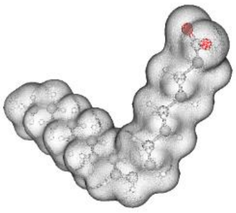	36.60	37.78	44.60	27.44
16	Methyl laurate	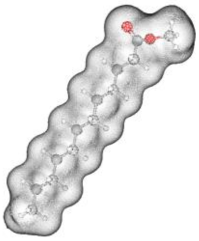	27.87	21.37	22.46	39.79

[a] Calculated with *ChemAxon* software.

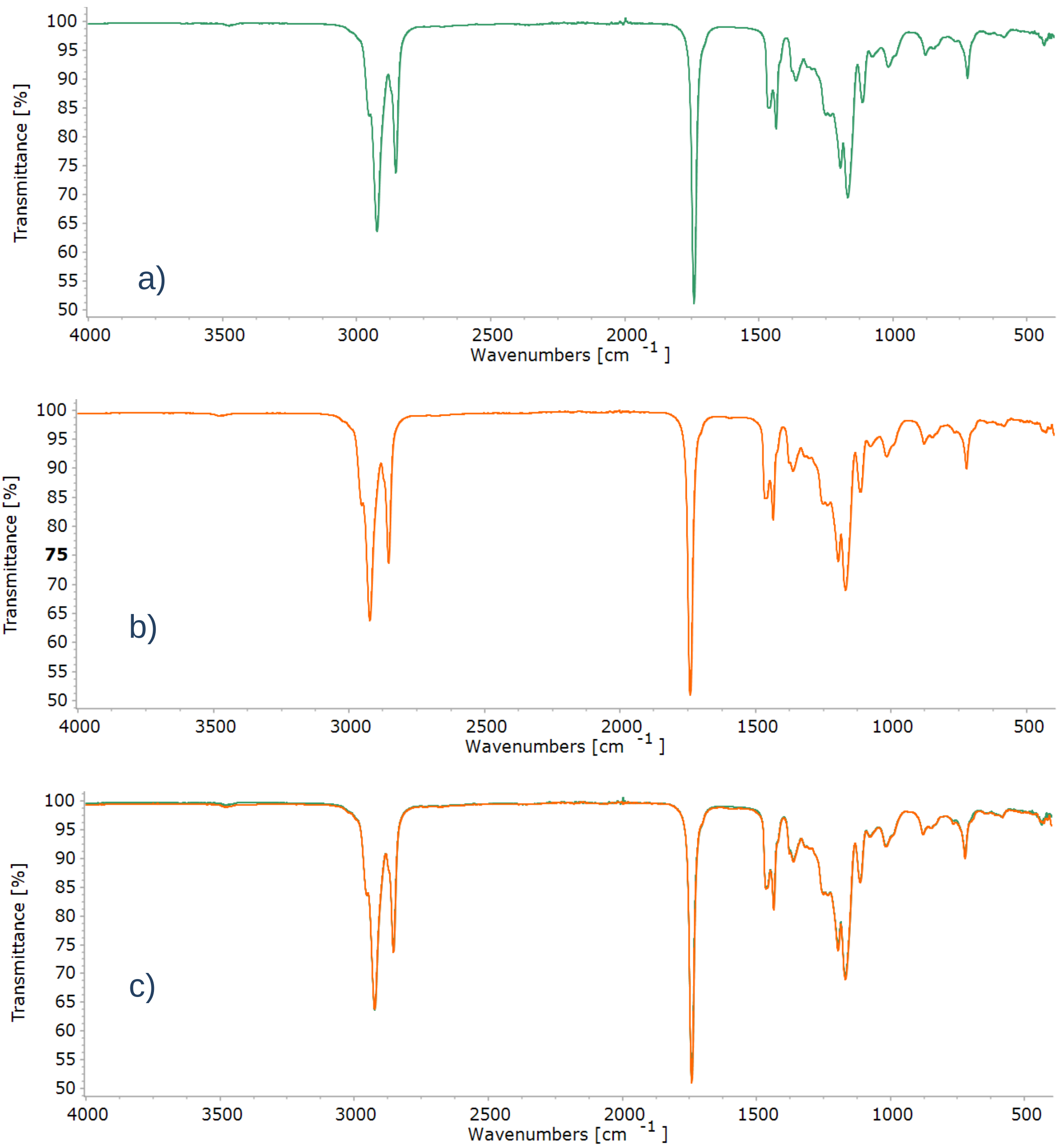

Figure S2: a) FTIR spectrum before reaction, b) Spectrum after reaction, c) Stacked spectra

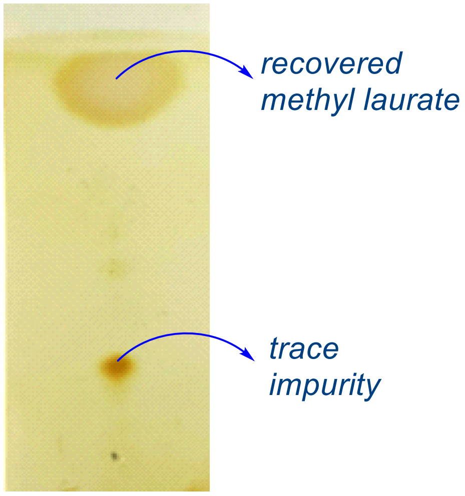

Figure S3: TLC plate of recovered methyl laurate (PE/EtOAc = 8/2, spots were made visible with iodine)

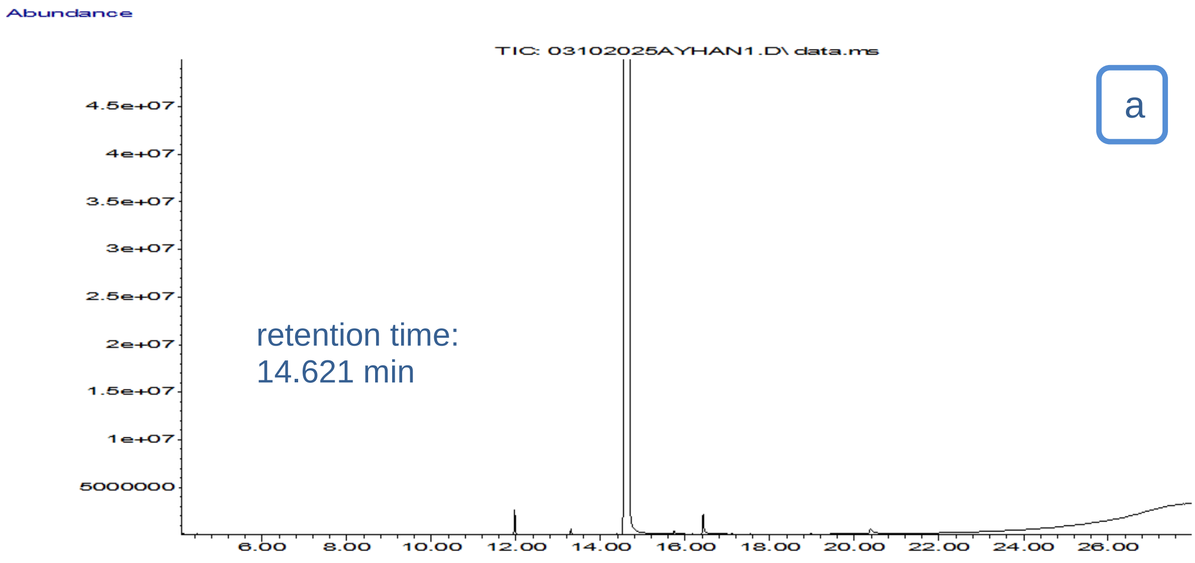

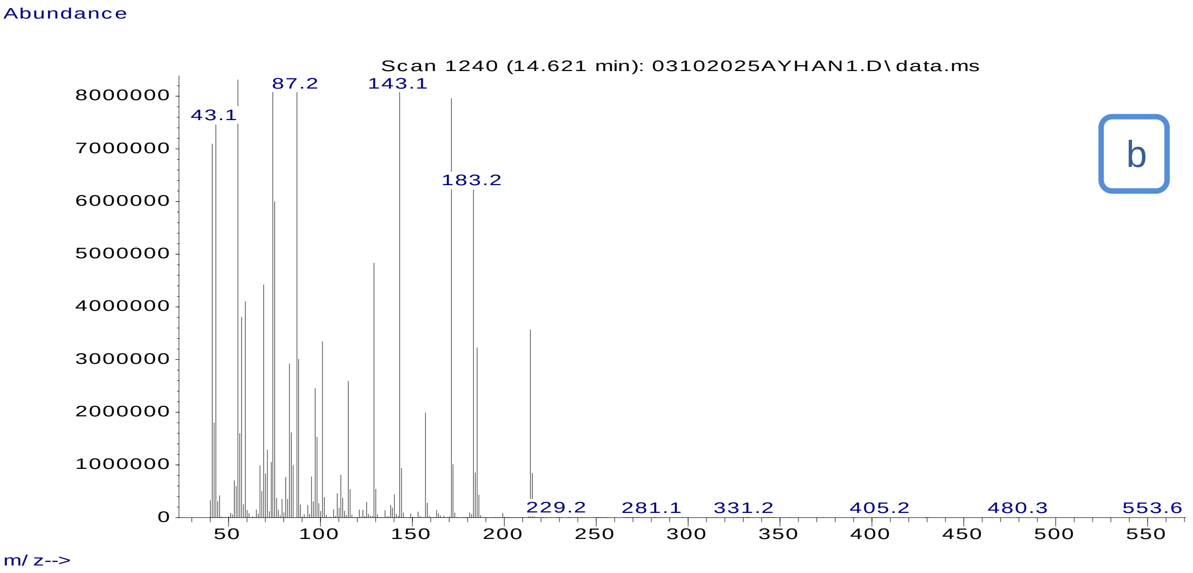

Figure S4: a) GC chromatogram of recovered methyl laurate. b) MS spectrum of recovered methyl laurate
